# STIL Overexpression Is Associated with Chromosomal Numerical Abnormalities in Non-Small-Cell Lung Carcinoma Through Centrosome Amplification

**DOI:** 10.3390/curroncol31120585

**Published:** 2024-12-12

**Authors:** Shunsuke Ohtsuka, Hisami Kato, Rei Ishikawa, Hirofumi Watanabe, Ryosuke Miyazaki, Shin-ya Katsuragi, Katsuhiro Yoshimura, Hidetaka Yamada, Yasuhiro Sakai, Yusuke Inoue, Yusuke Takanashi, Keigo Sekihara, Kazuhito Funai, Haruhiko Sugimura, Kazuya Shinmura

**Affiliations:** 1 Department of Tumor Pathology, Hamamatsu University School of Medicine, Hamamatsu 431-3192, Japan; shunpoi@gmail.com (S.O.); hisami@hama-med.ac.jp (H.K.); reipatho@hama-med.ac.jp (R.I.); d19033@hama-med.ac.jp (H.W.); 07486215@hama-med.ac.jp (R.M.); d20010@hama-med.ac.jp (S.-y.K.); ky@hama-med.ac.jp (K.Y.); h-yamada@hama-med.ac.jp (H.Y.); ya-sakai@hama-med.ac.jp (Y.S.); hsugimur@po.kyoundo.jp (H.S.); 2 Second Division, Department of Internal Medicine, Hamamatsu University School of Medicine, Hamamatsu 431-3192, Japan; yinoue@hama-med.ac.jp; 3 Department of Surgery 1, Hamamatsu University School of Medicine, Hamamatsu 431-3192, Japan; nashimed1@gmail.com (Y.T.); sekihara@hama-med.ac.jp (K.S.); kfunai@hama-med.ac.jp (K.F.); 4 Kyoundo Hospital, Sasaki Foundation, Tokyo 101-0062, Japan

**Keywords:** centrosome amplification, chromosomal instability, chromosomal numerical abnormality, lung adenocarcinoma, lung squamous cell carcinoma, non-small-cell lung carcinoma, overexpression, STIL

## Abstract

STIL is a regulatory protein essential for centriole biogenesis, and its dysregulation has been implicated in various diseases, including malignancies. However, its role in non-small-cell lung carcinoma (NSCLC) remains unclear. In this study, we examined STIL expression and its potential association with chromosomal numerical abnormalities (CNAs) in NSCLC using The Cancer Genome Atlas (TCGA) dataset, immunohistochemical analysis, and in vitro experiments with NSCLC cell lines designed to overexpress STIL. TCGA data revealed upregulated STIL mRNA expression in lung adenocarcinoma (LUAD) and lung squamous cell carcinoma (LUSC), the two major subtypes of NSCLC. Immunohistochemical analysis of cases from our hospital (LUAD, *n* = 268; LUSC, *n* = 98) revealed STIL protein overexpression. To elucidate the functional role of STIL, an inducible STIL-overexpressing H1299 NSCLC cell line was generated. Overexpression of STIL in these cells promoted centrosome amplification, leading to chromosomal instability. Finally, analysis of arm-level chromosomal copy number alterations from the TCGA dataset revealed that elevated STIL mRNA expression was associated with CNAs in both LUAD and LUSC. These findings suggest that STIL overexpression is associated with CNAs in NSCLC, likely through centrosome amplification, which is linked to chromosomal instability and might represent a potential therapeutic target for NSCLC treatment.

## 1. Introduction

According to the Global Cancer Statistics of GLOBOCAN 2022, lung cancer is the most frequently diagnosed cancer, accounting for 12.4% of all cases. It remains the leading cause of cancer-related deaths, contributing to 18.7% of total cancer deaths across both sexes combined [1]. Lung cancer encompasses several histopathological subtypes, with lung adenocarcinoma (LUAD) and lung squamous cell carcinoma (LUSC) widely recognized as the primary subtypes, which together constitute the majority of non-small-cell lung carcinoma (NSCLC) cases [2]. Recent large-scale molecular characterization initiatives, including The Cancer Genome Atlas (TCGA) project, have greatly enhanced our understanding of NSCLC at multiple molecular levels, including mRNA expression, mutations, and gene copy number alterations [3,4,5,6,7]. However, numerous aspects of NSCLC remain poorly understood, highlighting the global need for continued research in this area. As novel insights may emerge through alternative analytical approaches to these omics datasets, further exploration of such data holds considerable promise for advancing knowledge in this field.

STIL, also known as SIL, was initially identified at a genomic rearrangement site in a patient with T-cell acute lymphoblastic leukemia [8] and has been implicated in the regulation of centrosome integrity and mitotic spindle organization [9]. Subsequent studies have demonstrated that STIL is a critical regulator of PLK4 kinase activity, which is essential for initiating the duplication of centrioles—microtubule-based structures within the centrosome [10,11,12,13,14,15,16,17,18,19,20]. Experimental evidence has shown that overexpression of STIL induces centriole amplification (the presence of more than four centrioles within a cell) [11,12,15,16,18,20]. Centriole amplification is commonly referred to as centrosome amplification and is a significant contributor to chromosomal instability (CIN), leading to chromosomal numerical abnormalities (CNAs) and promoting tumor progression in various cancers [21,22,23,24]. Therefore, STIL upregulation may contribute to tumorigenesis through centrosome amplification. However, centrosome amplification resulting from ectopic STIL overexpression has only been observed in cell line analyses, specifically in U2OS osteosarcoma [11,12,15,16,20] and DLD-1 colorectal cancer cells [18], leaving it unclear whether this phenomenon also occurs in other cell types. Interestingly, STIL dysregulation has been reported in several human diseases, including microcephaly [25] and cancers of the lungs, colorectum, urinary bladder, liver, and breast [26,27,28,29,30,31]. In NSCLC, STIL overexpression has been documented and its role in tumor metastasis has been suggested through mechanisms beyond centrosome amplification [26,27]. However, the number of lung cancer cases analyzed by Erez et al. [26] was limited and Wang et al. [27] combined cases of LUAD and LUSC, the two major subtypes of NSCLC, in their protein expression analysis. Moreover, the association between STIL overexpression and clinicopathological factors, as well as its relationship with centrosome-amplification-related events such as CIN and CNAs, has not been thoroughly explored in NSCLC. Further investigations are necessary to elucidate the role of STIL in NSCLC tumorigenesis, particularly in relation to their potential impact on CIN, CNAs, and other clinicopathological factors.

Building on the findings presented in the preceding section and previous research demonstrating that CNAs are common features of NSCLC [3,4], we hypothesized that aberrant STIL expression contributes to the development of NSCLC through CNAs. To test this hypothesis, we examined the STIL mRNA and protein expression levels in primary NSCLC. By observing STIL overexpression in NSCLC, we investigated its effect on NSCLC cell lines and examined the relationship between STIL expression and chromosomal arm-level copy number alterations in primary NSCLC.

## 2. Materials and Methods

### 2.1. Primary Lung Carcinoma Tissues

Formalin-fixed paraffin-embedded (FFPE) tissue blocks from 268 primary LUAD and 98 primary LUSC cases were collected at Hamamatsu University Hospital (HUH) for immunohistochemical (IHC) analysis. The study protocol was approved by the Institutional Review Board of the Hamamatsu University School of Medicine [15-067 (approval data: 17 July 2015)/23-348 (27 February 2024)].

### 2.2. Public Data Collection

mRNA expression and gene copy number data for LUAD and LUSC were obtained from the TCGA data portal (https://tcga-data.nci.nih.gov/tcga/, accessed on 10 January 2017) and cBioPortal (https://www.cbioportal.org). The LUAD dataset comprised 59 non-tumorous and 515 tumor samples, whereas the LUSC dataset contained 51 non-tumorous and 501 tumor samples. The expression data were obtained as processed RNA-seq data in the form of RNA-seq by Expectation Maximization (RSEM) [32]. Gene copy number data generated by the Genomic Identification of Significant Targets in Cancer (GISTIC) algorithm [33] were also obtained.

Microarray-based gene expression data (GSE75037) for 83 LUADs and their corresponding normal lung tissues, previously published by Girard et al. [34], were also downloaded from the Gene Expression Omnibus (GEO) at the National Center for Biotechnology Information (NCBI).

### 2.3. IHC Analysis

The FFPE sections were incubated with a rabbit anti-STIL polyclonal antibody (antigen retrieval: Tris-EDTA buffer, pH 9.0; dilution ratio, 1:150; Bioss, Woburn, MA, USA), followed by an HRP-conjugated polymer (Histofine Simple Stain MAX PO, Nichirei, Tokyo, Japan). Visualization was achieved using 3,3′-diaminobenzidine tetrahydrochloride. To assess the protein expression level, IHC scores were calculated by multiplying the staining intensity value (0, absent; 1, weak; 2, moderate; 3, strong) in the cytoplasm by the percentage of cells with each intensity value (0–100%) to produce a score ranging from 0 to 300.

### 2.4. Cell Cultures

Human NSCLC cell line H1299 was obtained from the American Type Culture Collection (Manassas, VA, USA). H1299 cells and their derivatives were maintained at 37 °C in RPMI1640 medium supplemented with 10% fetal bovine serum and penicillin/streptomycin under a 5% CO_2_ atmosphere.

### 2.5. Establishment of Stable Inducible Cell Lines

FLAG-STIL was amplified using polymerase chain reaction with PrimeSTAR HS DNA polymerase (Takara, Kyoto, Japan) and subsequently cloned into a PiggyBac cumate switch-inducible vector (System Biosciences, Mountain View, CA, USA) at the *Not*I restriction site. To facilitate the use of the co-transfected GFP signal for immunofluorescence analysis, the CopGFP sequence in the PiggyBac cumate switch-inducible vector was destroyed using site-directed mutagenesis in our previous study [35]. Information on the primers used for the construction and confirmation of the FLAG-STIL/PiggyBac cumate switch-inducible vector is summarized in Supplementary Table S1. A single-cell clone of the H1299 cell line was obtained by limiting dilution, and a H1299 cell clone was transfected with the FLAG-STIL expression construct together with the PiggyBac transposase vector (System Biosciences) and selected using puromycin (1.6 μg/mL: Clontech, Palo Alto, CA, USA). Additionally, cells transfected with an empty (parental) PiggyBac cumate switch-inducible vector and a transposase vector were used as controls.

### 2.6. Western Blot Analysis

STIL-inducible or empty vector-transposed cell lines were treated with or without cumate for 72 h and lysed in a buffer containing 50 mM Tris-HCl (pH 7.5), 150 mM NaCl, 0.1% sodium dodecyl sulfate, 1% Triton X-100, 1.0% sodium deoxycholate, 100 mM sodium fluoride, 1 mM sodium orthovanadate, and a protease inhibitor cocktail (Sigma-Aldrich, St. Louis, MO, USA). Western blot analysis was performed using rabbit anti-STIL (Bethyl Laboratories, Montgomery, TX, USA) and rabbit anti-GAPDH polyclonal antibodies (GeneTex, San Antonio, TX, USA). Immunoreactivity was visualized using an ECL Plus chemiluminescence system (GE Healthcare Bio-Science, Piscataway, NJ, USA), as previously described [36]. Protein band intensity was measured using the ImageJ software (version 1.46) (National Institutes of Health, Bethesda, MD, USA).

### 2.7. Indirect Immunofluorescence Analysis

STIL-inducible cell lines or empty vector-transposed cell lines were treated with or without cumate for 72 h. Following methanol fixation, cells were incubated with mouse anti-centrin (CETN2) monoclonal antibody (clone 20H5; Millipore, Bedford, MA, USA) followed by Alexa Fluor 594-conjugated secondary antibody (Molecular Probes, Eugene, OR, USA), and the nuclei were stained with 4′,6-diamidino-2-phenylindole (DAPI). Fluorescence signals were observed using an inverted fluorescence microscope (IX83; Olympus, Tokyo, Japan). For the detection of centrin foci tagged with GFP, the cells were treated with cumate for 72 h, and after re-seeding, the cells were transfected with a GFP-centrin (CETN2) expression vector, which was constructed by inserting CETN2 into pEGFP-C1 (Clontech) [37,38] using Lipofectamine 2000 reagent (Thermo Fisher Scientific, Waltham, MA, USA). At 24 h after transfection, the cells were fixed with methanol, and GFP-centrin was observed using a fluorescence microscope (Olympus IX83).

### 2.8. Metaphase Spread and Fluorescence In Situ Hybridization (FISH)

STIL-inducible cell lines or empty vector-transposed cell lines were treated with or without cumate for 6 days. After nocodazole treatment (Sigma-Aldrich), the cells were trypsinized and collected by centrifugation in a conical tube. The cells were gently suspended with hypotonic solution (75 mM KCl) and incubated at 37 °C for 22 min. A pre-chilled fixative solution consisting of methanol and glacial acetic acid in a 3:1 ratio was carefully applied to the cells. Following application, the cells were subjected to centrifugation, and the resulting cell pellet was collected. This process was repeated for a total of three cycles. The fixed cells were dropped onto low-fluorescence glass slides (Matsunami, Osaka, Japan). Slide glasses were incubated in citrate buffer solution at 95 °C for 20 min, followed by washing with distilled water, and a ready-to-use Cy3-labeled pan-centromeric DNA probe (Cambio, Cambridge, UK) was applied to the slides, which were then covered with coverslips. The sections were denatured on a thermal plate at 85 °C for 5 min, and hybridization was performed overnight at 37 °C. After hybridization, the slides were washed with 2× saline sodium citrate buffer containing 0.3% NP-40 at 75 °C for 2 min. The slides were washed and mounted with glass coverslips using a mounting medium with DAPI (Molecular Probes) for subsequent observation under a fluorescence microscope (Olympus IX83).

### 2.9. CNA Analysis

For each case of LUAD (*n* = 515) and LUSC (*n* = 501) from the TCGA dataset, the chromosomal arm-level copy number status was assessed. A chromosomal arm was categorized as a deletion if two-thirds or more of the corresponding genes had a GISTIC-based copy number of “−2” or “−1”. Similarly, it was classified as amplified/gained if two-thirds or more of the genes exhibited a GISTIC-based copy number of “2” or “1”. Chromosomal arms that did not meet either criteria were classified as diploid. Subsequently, for each case, we counted the number of short and long arms across all chromosomes that displayed deletion, amplification/gain, or diploid status and recorded the number of arms with either deletion or amplification/gain. The median number of arms with deletions or amplifications/gains was then calculated for the LUAD and LUSC cases. Each case was classified as CNA-high if the number of arms with deletion or amplification/gain was equal to or above the median value and as CNA-low if the number of such arms was below the median. The analytical workflow is depicted in Supplementary Figure S1. Owing to incomplete data, 3 cases from each group were excluded, resulting in 512 LUAD and 498 LUSC cases classified as either CNA-high or CNA-low.

### 2.10. Statistical Analysis

Statistical analyses were conducted using the Mann–Whitney *U* test, unpaired *t*-test, Fisher’s exact test, or Wilcoxon signed-rank test. These analyses were performed using JMP software version 9.0 (SAS Institute, Cary, NC, USA) or GraphPad QuickCalcs https://www.graphpad.com/quickcalcs/, accessed on 16 February 2018 (GraphPad Software Inc., San Diego, CA, USA). A *p*-value of less than 0.05 was considered indicative of statistical significance.

## 3. Results

### 3.1. Overexpression of STIL mRNA Transcripts and Proteins in Primary NSCLC

**Figure 1:**
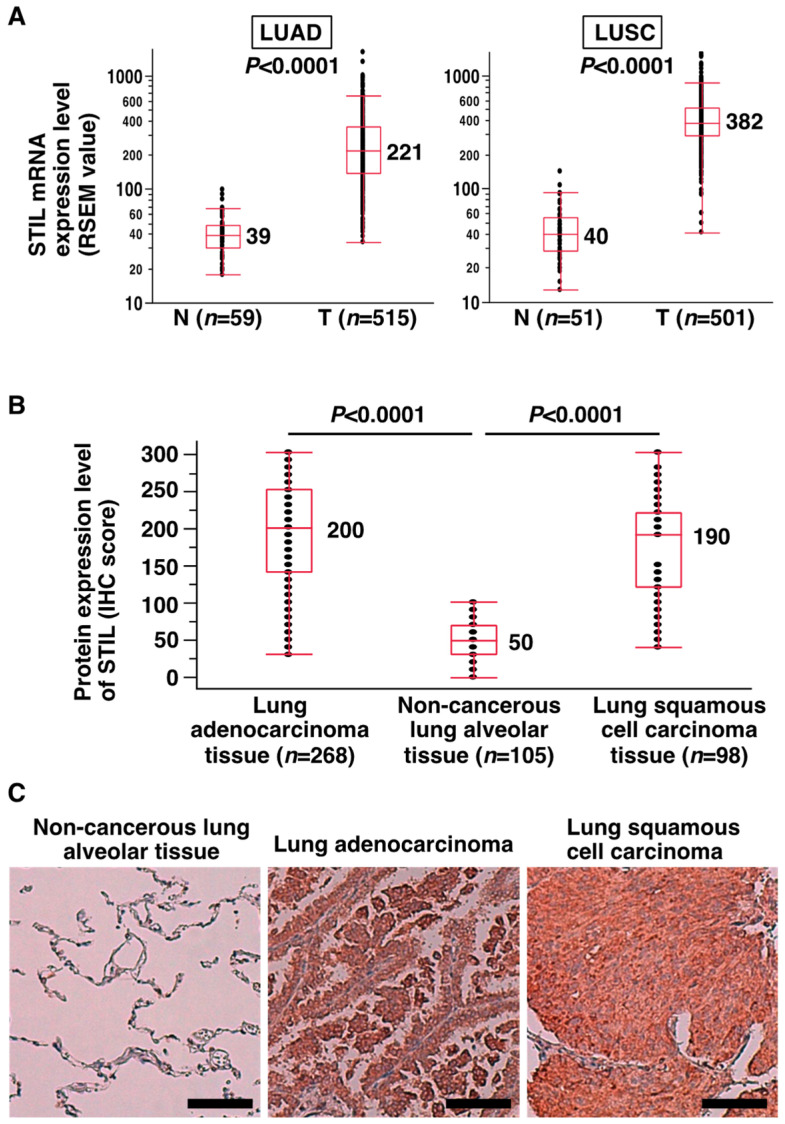
Overexpression of STIL mRNA and protein in primary NSCLC. (**A**) STIL mRNA overexpression in LUAD and LUSC based on the TCGA database analysis. Statistical comparison between non-cancerous tissues (N) and cancerous tissues (T) was performed using the Mann–Whitney *U* test, with *p*-values and median expression levels reported. (**B**) STIL protein overexpression in LUAD and LUSC was assessed by IHC analysis using a rabbit anti-STIL polyclonal antibody on samples obtained from our institution. Statistical comparisons of STIL expression between non-cancerous lung alveolar tissue and LUAD, as well as between non-cancerous lung alveolar tissue and LUSC, were conducted using the Mann–Whitney *U* test, with median expression levels and *p*-values reported. (**C**) Representative IHC images illustrating STIL protein expression in primary LUAD and LUSC. Scale bar = 100 μm.

To determine the status of STIL expression in NSCLC, we examined STIL mRNA expression levels in primary LUAD (59 non-tumorous and 515 tumor samples) and primary LUSC (51 non-tumorous and 501 tumor samples) using the TCGA dataset. The results showed that STIL mRNA expression levels were significantly higher in LUAD compared to non-cancerous lung tissues (median expression value: 221 vs. 39; *p* < 0.0001, Mann–Whitney *U* test) and were also significantly higher in LUSC compared to non-cancerous lung tissues (median expression value: 382 vs. 40; *p* < 0.0001) (Figure 1A). Additionally, since the GEO data (GSE75037) contained a large number of matched pairs (i.e., matched non-tumorous and tumorous tissues) of NSCLC [34], we examined STIL expression and found that STIL mRNA expression levels were significantly higher in cancerous tissues than in matched non-cancerous lung tissues (*p* < 0.0001 by Wilcoxon’s signed-rank test) (Supplementary Figure S2). Next, we investigated whether the STIL protein was overexpressed in NSCLC. An IHC analysis using anti-STIL antibody was performed for 268 primary LUAD cases and 98 primary LUSC cases collected in our hospital (HUH), and the results showed that the STIL protein was predominantly localized in the cytoplasm, which is consistent with previous reports [39,40], and that its expression level was significantly higher in the LUAD tissues (median value of IHC score, 200; *p* < 0.0001 by Mann–Whitney *U* test) and in the LUSC tissues (median value, 190; *p* < 0.0001) than in the non-cancerous lung alveolar tissues (median value, 50) (Figure 1B,C). When the IHC score of 150, which was three times the median value of the IHC score in the non-cancerous lung alveolar tissues, was used as a cutoff value to dichotomize the IHC score of the cancer cases, higher STIL protein expression (>150) was detected in 64.9% (174/268) of LUAD cases and 51.0% (50/98) of LUSC cases. Next, we investigated whether the differences in STIL protein expression levels based on this dichotomization were associated with any clinicopathological factors among patients with NSCLC. The results showed that a high STIL protein expression level was associated with higher pT (*p* = 0.0262) and pN (*p* = 0.0171) factor scores and higher TNM stage (*p* = 0.0076) in LUAD (Table 1) and with a higher pT factor score (*p* = 0.0027) and higher TNM stage (*p* = 0.0051) in LUSC (Table 2). These findings indicate that STIL is overexpressed in a substantial subset of NSCLC cases and that its overexpression is associated with advanced pathological stages of the disease.

### 3.2. STIL Expression Levels Define the Centrosome Amplification Status in NSCLC Cell Lines

**Figure 2:**
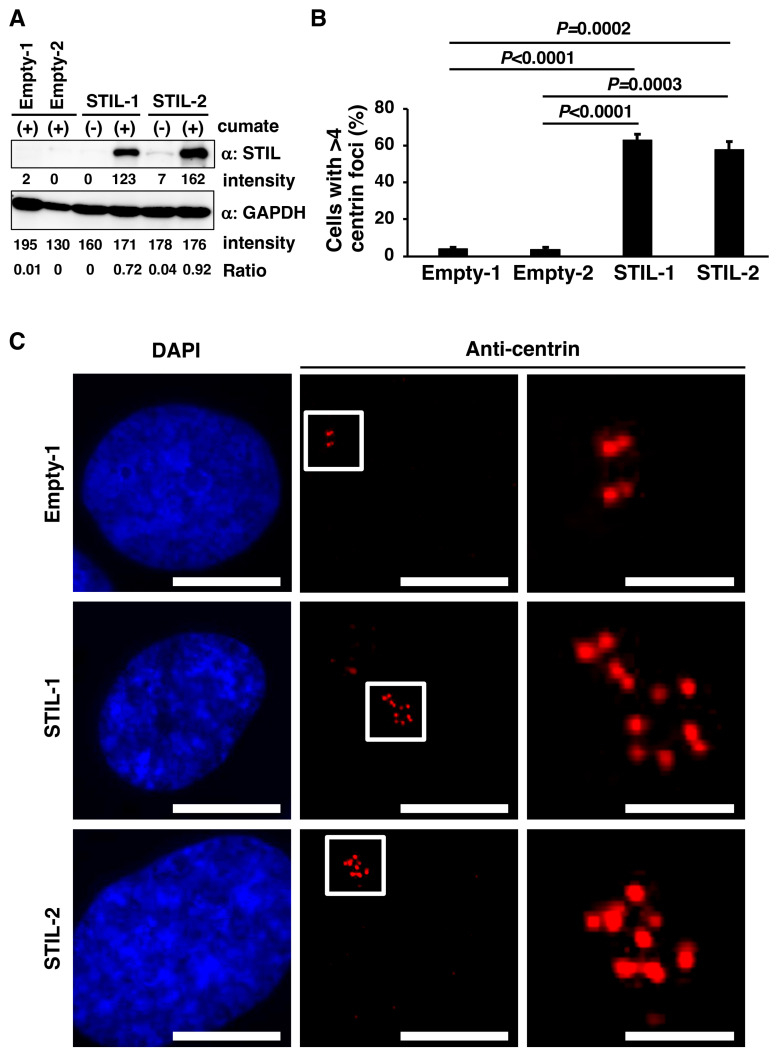
Centrosome amplification induction by STIL overexpression in the H1299 NSCLC cell line. (**A**) Detection of STIL protein levels in two cumate-inducible stable H1299 NSCLC cell lines, termed STIL-1 and STIL-2. Both cell lines were engineered to express FLAG-STIL in the presence of cumate. Increased STIL protein expression was shown via Western blot analysis using anti-STIL antibody. Two empty vector-transposed cell lines, designated Empty-1 and Empty-2, were used as controls. GAPDH expression served as the internal loading control. The protein band intensities and the expression ratio of STIL to GAPDH are presented. (**B**,**C**) Effect of STIL overexpression on centrosome amplification in H1299 cells. Cumate-inducible stable H1299 cell lines overexpressing STIL and corresponding empty vector controls were treated with cumate for 72 h. Following fixation, the cells were immunostained with an anti-centrin antibody (red) to visualize the centrioles, and the nuclei were counterstained with DAPI (blue). The percentage of cells containing more than four centrin foci (centrioles) was quantified and is presented in the bar graph in panel (**B**). Data represent the mean ± standard error from three independent experiments. Statistical significance was assessed using an unpaired *t*-test. Representative immunofluorescence images are shown in panel (**C**). Insets in the middle panels are magnified in the right panels. Scale bar = 10 μm (left and middle panels); 2.5 μm (right panels).

Next, we investigated the effects of STIL overexpression in human NSCLC cell lines. First, we generated H1299 NSCLC cell lines capable of inducing FLAG-STIL protein expression, along with control H1299 cell lines, using the PiggyBac transposon vector system. This included two STIL-transposed clones (designated STIL-1 and STIL-2) and two empty vector-transposed clones (designated Empty-1 and Empty-2) (Figure 2A; Original data are shown in Supplementary Figure S3). Western blot analyses with anti-STIL antibody showed that STIL-transposed clones, but not empty vector-transposed clones, induced the expression of FLAG-STIL after cumate treatment. We compared the number of centrioles corresponding to centrin foci between empty vector-transposed clones and STIL-transposed clones by immunofluorescence analysis using an anti-centrin antibody. The results showed that the percentage of cells with more than four centrioles (centrin foci), which is indicative of centrosome amplification, was significantly higher in the STIL-overexpressing clones than in the empty vector-transposed clones (Figure 2B; representative results are shown in Figure 2C). An increased percentage of cells with more than four centrioles (centrin foci) was also observed in STIL-overexpressing clones transiently transfected with a GFP-centrin expression vector, but not in empty vector-transposed clones transiently transfected with the GFP-centrin expression vector (Supplementary Figure S4). These results suggest that lung cancer cells with higher STIL expression levels show centrosome amplification compared to cells with lower STIL expression levels.

### 3.3. STIL Overexpression Induces CIN in NSCLC Cell Lines

**Figure 3:**
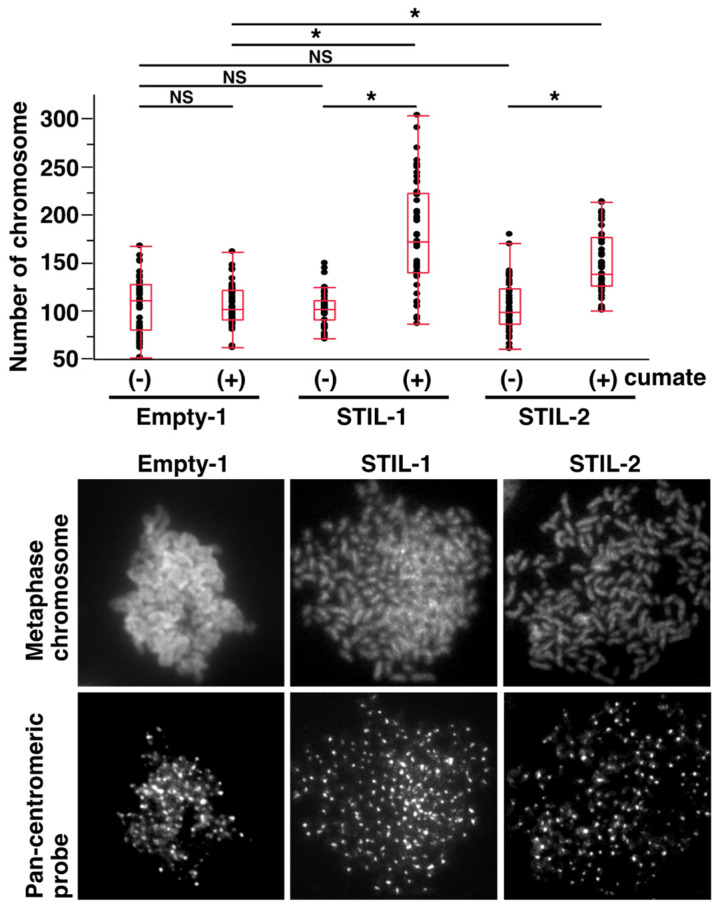
Induction of CIN by STIL overexpression in the H1299 NSCLC cell line. Stable H1299 NSCLC cell lines designed to express STIL by cumate treatment and empty vector-transposed H1299 cell lines were treated with or without cumate for 6 days. Stable H1299 lung cancer cell lines, either transposed by an empty vector or engineered to express STIL upon cumate induction, were treated with or without cumate for 6 days. Metaphase spreads were then prepared, and FISH analysis was performed using a Cy3-labeled pan-centromeric DNA probe to quantify the number of chromosomes. The results are presented as a box plot, and statistical comparison between the groups was conducted using the Mann–Whitney *U* test. * indicates *p* < 0.0001, and NS indicates no significant difference. Representative images are shown in the panels below the graph.

Next, we investigated whether STIL expression levels defined CNA status resulting from CIN in human NSCLC cell lines. H1299 cell lines with inducible expression of the FLAG-STIL protein (STIL-1 and -2) and a corresponding empty vector-transduced control line (Empty-1) were treated with or without cumate for six days. Following treatment, metaphase spreads were prepared from these cells, and FISH analysis was performed using a pan-centromeric probe to determine chromosome numbers. Consistent with previous studies reporting the chromosome number of H1299 cells [41], the median chromosome number of Empty-1, STIL-1, and -2 cells without cumate treatment was ~100 (Figure 3). Upon cumate treatment, the chromosome number was significantly increased in both STIL-1 and -2, but not in Empty-1 (Figure 3). These results suggest that STIL overexpression induces CIN, likely due to centrosome amplification, and potentially contributes to CNA progression in NSCLC.

### 3.4. Association of Increased STIL Expression with Progressive CNAs in Primary NSCLC

**Figure 4:**
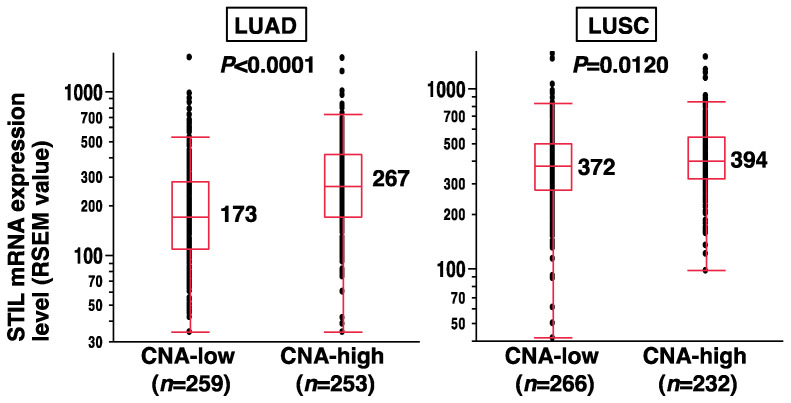
Association between STIL expression levels and CNA status in primary NSCLC. Copy number alteration data for whole chromosomal genes in LUAD and LUSC were retrieved from the TCGA dataset. Using the data, chromosomal arm-level copy number status was constructed for each case, and then, based on the results, each case was categorized as either CNA-low or CNA-high. STIL mRNA expression levels were also obtained from TCGA. A Mann–Whitney *U* test was performed to compare STIL expression levels between the CNA-low and CNA-high groups. The *p*-values and median expression levels are presented.

Finally, we investigated the effect of STIL overexpression on CNAs in primary NSCLC using data from the TCGA database. Based on the copy number data of whole genes, the chromosomal arm-level copy number status was calculated in each case, and the median value of chromosomal arms with deletion or amplification/gain was used as a cut-off value to dichotomize the chromosomal arm-level copy number status of the cases, which were classified as CNA-low and CNA-high [Supplementary Table S2 for LUAD (*n* = 512) and Supplementary Table S3 for LUSC (*n* = 498)]. When STIL mRNA expression levels were compared between CNA-low and CNA-high groups, STIL expression was significantly higher in CNA-high cases than in CNA-low cases in LUAD (*p* < 0.0001) and LUSC (*p* = 0.0120) (Figure 4). These results suggest an association between increased STIL expression and progressive CNAs, likely resulting from CIN, in primary NSCLC.

## 4. Discussion

In this study, we demonstrated that STIL expression is significantly upregulated at both the mRNA and protein levels in primary NSCLC and that its overexpression is associated with more advanced pathological stages. We established H1299 NSCLC cell lines that overexpressed STIL and found that STIL overexpression in H1299 cells induced both centrosome amplification and CIN. Finally, analysis of the arm-level copy numbers of whole chromosomes revealed that elevated STIL expression was associated with CNAs in primary NSCLC. These findings suggest that STIL overexpression contributes to CNA development in NSCLC, likely through centrosome amplification, which drives CIN. Our study provides mechanistic insights into previously observed CNAs in NSCLC [3,4] and establishes a novel link between STIL overexpression and lung cancer carcinogenesis.

Centrosome amplification is a known characteristic of NSCLC [38,42], often associated with mitotic spindle abnormalities, lagging chromosomes, and increased errors in merotelic kinetochore-microtubule attachments, all contributing to CIN via chromosomal mis-segregation [21,23,43,44,45]. In this study, we provided experimental evidence that STIL overexpression induces centrosome amplification and CIN in an NSCLC cell line, which is a novel finding in the context of NSCLC. Moreover, a comprehensive analysis of whole-gene copy number alteration status derived from TCGA datasets revealed that elevated STIL expression was associated with CNAs in primary NSCLC patients. Because CNAs, also referred to as chromosomal aneuploidies, are the cumulative result of CIN observed at a specific clinical time point, it is plausible that the CNAs observed in NSCLC cases with STIL overexpression are attributable to centrosome-amplification-induced CIN. In our CNA analysis, tumor ploidy information was excluded from the calculations due to the absence of corresponding data in the TCGA dataset. Given that centrosome amplification is closely associated with chromosomal mis-segregation, incorporating ploidy data into CNA evaluation could provide a more accurate assessment. We plan to address this limitation in future studies.

Our analysis also demonstrated that STIL overexpression occurred in both LUAD and LUSC at both the mRNA and protein levels. This study analyzed 366 lung cancer cases, a larger cohort than the prior studies by Erez et al. [26] and Wang et al. [27], providing robust evidence that STIL is overexpressed in both LUAD and LUSC. Furthermore, our data demonstrated an association between STIL overexpression and the advanced pathological stages of NSCLC. Given that CIN is known to enhance the malignant potential of tumors [46,47] and considering the findings of Wang et al. that STIL promotes tumor cell proliferation and invasion in NSCLC [27], it is likely that the increased malignant phenotype resulting from STIL overexpression contributes to the worsened pathological stage observed in NSCLC patients with elevated STIL expression.

STIL overexpression has been reported in several types of cancers, including lung, colorectal, urinary bladder, liver, and breast [26,27,28,29,30,31]. In NSCLC, STIL overexpression promotes epithelial–mesenchymal transition (EMT) and stemness through the HIF1α-STIL-FOXM1 axis [27]. Combined with our current findings on CIN/CNAs associated with STIL overexpression via centrosome amplification in NSCLC, we hypothesize that STIL overexpression is strongly linked to malignant tumor progression, tumor heterogeneity, and drug resistance (Supplementary Figure S5). Other studies have highlighted the additional roles of STIL overexpression in cancers other than NSCLC. In urinary bladder cancer, STIL contributes to cell proliferation by inhibiting primary cilia formation through its interaction with AURKA [29], while in triple-negative breast cancer, it promotes malignancy by activating the Fanconi anemia pathway through KLF16 interactions [31]. Thus, it is evident that STIL overexpression plays a multifaceted role in human cancers.

Several studies have suggested that STIL overexpression may serve as a potential therapeutic target for cancer. STIL knockdown has been shown to suppress malignant characteristics such as tumor proliferation, migration, invasion, and metastasis in cancers of the lung, colorectum, bladder, liver, and breast [27,28,29,30,31]. Additionally, in hepatocellular carcinoma, STIL expression has been linked to immune cell infiltration and immune checkpoint expression and has been proposed as a predictive marker for immunotherapy and chemotherapy efficacy [30]. These findings underscore the potential of targeting STIL as a therapeutic strategy for NSCLC, possibly in combination with existing molecular therapies [48]. Future studies should focus on evaluating the feasibility and efficacy of integrating STIL-targeted therapies with established treatments such as EGFR inhibitors or immune checkpoint blockades. The synergistic potential of such combinations could target multiple oncogenic pathways, including CIN and immune evasion, which are critical drivers of NSCLC progression [49]. For example, inhibiting STIL may enhance the therapeutic efficacy of EGFR inhibitors by mitigating resistance mechanisms associated with CIN. Similarly, combining STIL inhibition with immune checkpoint therapies could amplify antitumor immune responses, as CIN has been linked to an increased tumor neoantigen burden, thereby enhancing tumor immunogenicity [50].

This study had two primary limitations. First, although the in vitro cell line experiments and CNA analyses using TCGA data clearly demonstrated the effects of STIL overexpression in NSCLC, these findings have not been confirmed in primary NSCLC tissues of our own or patient-derived models. Such validation would strengthen our conclusions regarding the impact of STIL overexpression on NSCLC. Second, the study focused on the associations between STIL overexpression, centrosome amplification, and CNAs, but did not delve into the precise molecular mechanisms underlying these relationships. Furthermore, although STIL has been proposed as a potential therapeutic target, the study lacks experimental evidence regarding the feasibility and efficacy of targeting STIL. Future research should address these gaps to strengthen the translational impact of these findings.

## 5. Conclusions

These findings suggest that STIL overexpression is associated with CNAs in NSCLC, likely through centrosome-amplification-induced CIN, and may represent a potential therapeutic target for NSCLC.

## Figures and Tables

**Table 1:** Clinicopathological factors of 268 patients with primary LUAD according to the STIL protein expression level.

Factor	No. of Cases	STIL Protein Expression Level ^§^		*p*-Value ^†^
		Low (*n* = 94)	High (*n* = 174)	
Age				
<60	74	29 (39.2%)	45 (60.8%)	0.3936
≥60	194	65 (33.5%)	129 (66.5%)	
Sex				
Female	111	36 (32.4%)	75 (67.6%)	0.5161
Male	157	58 (36.9%)	99 (63.1%)	
Smoking				
Non-smoker	99	31 (34.4%)	59 (65.6%)	1.000
Smoker	123	42 (34.2%)	81 (65.9%)	
pT				
pT1/pT2	237	89 (37.6%)	148 (62.5%)	0.0262
pT3/pT4	31	5 (16.1%)	26 (83.9%)	
pN				
pN0	197	77 (39.1%)	120 (60.9%)	0.0171
pN1-pN3	66	15 (22.7%)	51 (77.3%)	
TNM Stage				
I/II	219	85 (38.8%)	134 (61.2%)	0.0076
III/IV	49	9 (18.4%)	40 (81.6%)	

^§^ Low, IHC score ≤ 150; High, IHC score > 150 ^†^ Fisher’s exact test.

**Table 2:** Clinicopathological factors of 98 patients with primary LUSC according to the STIL protein expression level.

Factor	No. of Cases	STIL Protein Expression Level ^§^		*p*-Value ^†^
		Low (*n* = 48)	High (*n* = 50)	
Age				
<60	16	9 (56.3%)	7 (43.8%)	0.5916
≥60	82	39 (47.6%)	43 (52.4%)	
Sex				
Female	6	3 (50.0%)	3 (50.0%)	1.0000
Male	92	45 (48.9%)	47 (51.1%)	
Smoking				
Non-smoker	4	1 (25.0%)	3 (75.0%)	0.6197
Smoker	74	35 (47.3%)	39 (52.7%)	
pT				
pT1/pT2	72	42 (58.3%)	30 (41.7%)	0.0027
pT3/pT4	26	6 (23.1%)	20 (76.9%)	
pN				
pN0	62	32 (51.6%)	30 (48.4%)	0.8312
pN1-pN3	34	16 (47.1%)	18 (52.9%)	
TNM Stage				
I/II	66	39 (59.1%)	27 (40.9%)	0.0051
III/IV	32	9 (28.1%)	23 (71.2%)	

^§^ Low, IHC score ≤ 150; High, IHC score > 150 ^†^ Fisher’s exact test.

## Data Availability

The data presented in this study are available on request from the corresponding author.

## Supplementary Materials

The following supporting information can be downloaded at: https://www.mdpi.com/article/10.3390/curroncol31120585/s1, Figure S1: Workflow of CNA analysis; Figure S2: Comparison of STIL mRNA expression in NSCLC tissues versus corresponding normal tissues utilizing the GEO dataset (GSE75037); Figure S3: Original data of Figure 2A; Figure S4: Effect of STIL overexpression on centrosome amplification in H1299 NSCLC cells; Figure S5: A schematic representation illustrating the functional role of STIL overexpression; Table S1: Information of the primers used for the construction and validation of the FLAG-STIL/PiggyBac cumate switch-inducible vector; Table S2: Chromosomal arm-level copy number status in 512 LUAD cases; Table S3: Chromosomal arm-level copy number status in 498 LUSC cases.
