# Supplementary material for: STIL Overexpression Is Associated with Chromosomal Numerical Abnormalities in Non-Small-Cell Lung Carcinoma Through Centrosome Amplification
Source: Curr Oncol. 2024 Dec 12;31(12):7936–49. doi: 10.3390/curroncol31120585 (PMC11674966; doi:10.3390/curroncol31120585)
Supplement: Supplementary file 1 [file curroncol-31-00585-s001.zip › curroncol-3316633-supplementary.pdf]

Supplementary Information

STIL Overexpression Is Associated with Chromosomal Numerical Abnormalities in Non-Small-Cell Lung Carcinoma through Centrosome Amplification

Supplementary Figure S1: Workflow of CNA analysis

Supplementary Figure S2: Comparison of STIL mRNA expression in NSCLC tissues versus corresponding normal tissues utilizing GEO dataset (GSE75037)

Supplementary Figure S3: Original data of Figure 2A

Supplementary Figure S4: Effect of STIL overexpression on centrosome amplification in H1299 NSCLC cells

Supplementary Figure S5: A schematic representation illustrating the functional role of STIL overexpression

Supplementary Table S1: Information of the primers used for the construction and validation of the FLAG-STIL/PiggyBac cumate switch inducible vector

Supplementary Table S2: Chromosomal arm-level copy number status in 512 LUAD cases

Supplementary Table S3: Chromosomal arm-level copy number status in 498 LUSC cases

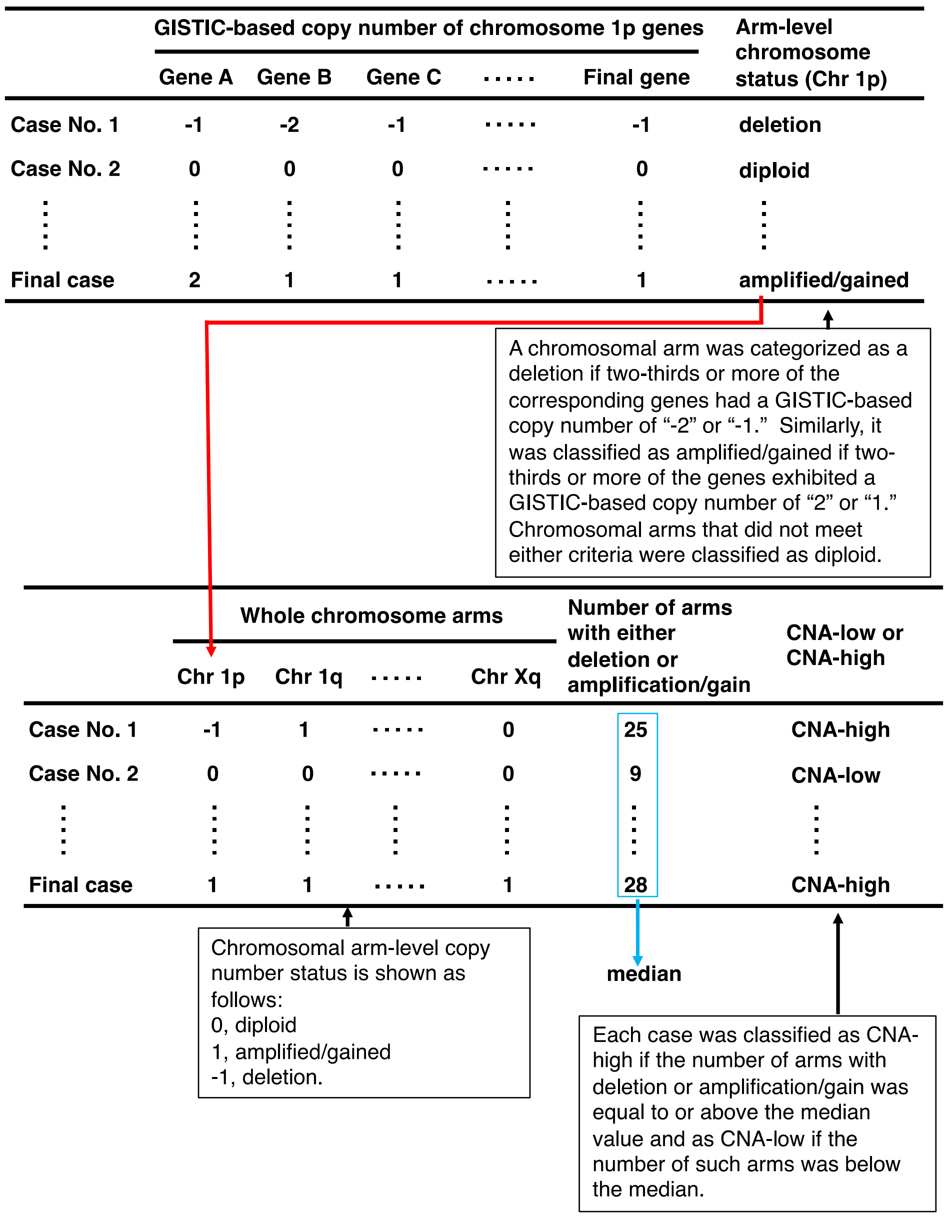

Supplementary Figure S1. Workflow of CNA analysis.

CNA status based on chromosomal arm-level copy number data was assessed as illustrated in this figure.

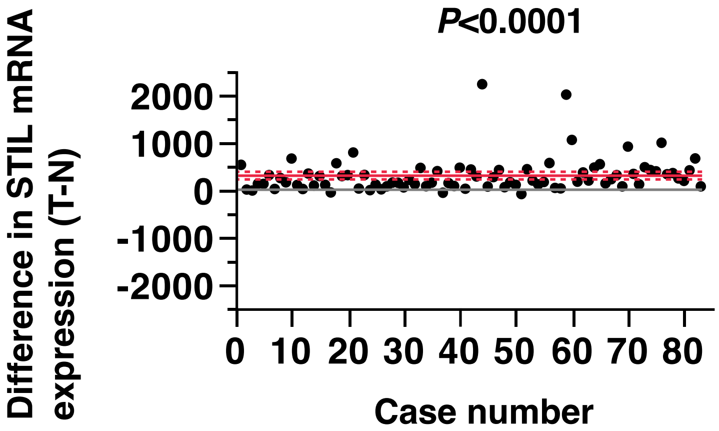

Supplementary Figure S2. Comparison of STIL mRNA expression in NSCLC tissues versus corresponding normal tissues utilizing GEO dataset (GSE75037).

STIL mRNA expression in paired cancerous (T) and non-cancerous (N) lung tissues from 83 NSCLC (lung adenocarcinoma) samples (GSE75037) [34] was analyzed. STIL expression was significantly elevated in cancerous tissues compared to non-cancerous tissues, as assessed by Wilcoxon signed-rank test (P<0.0001).

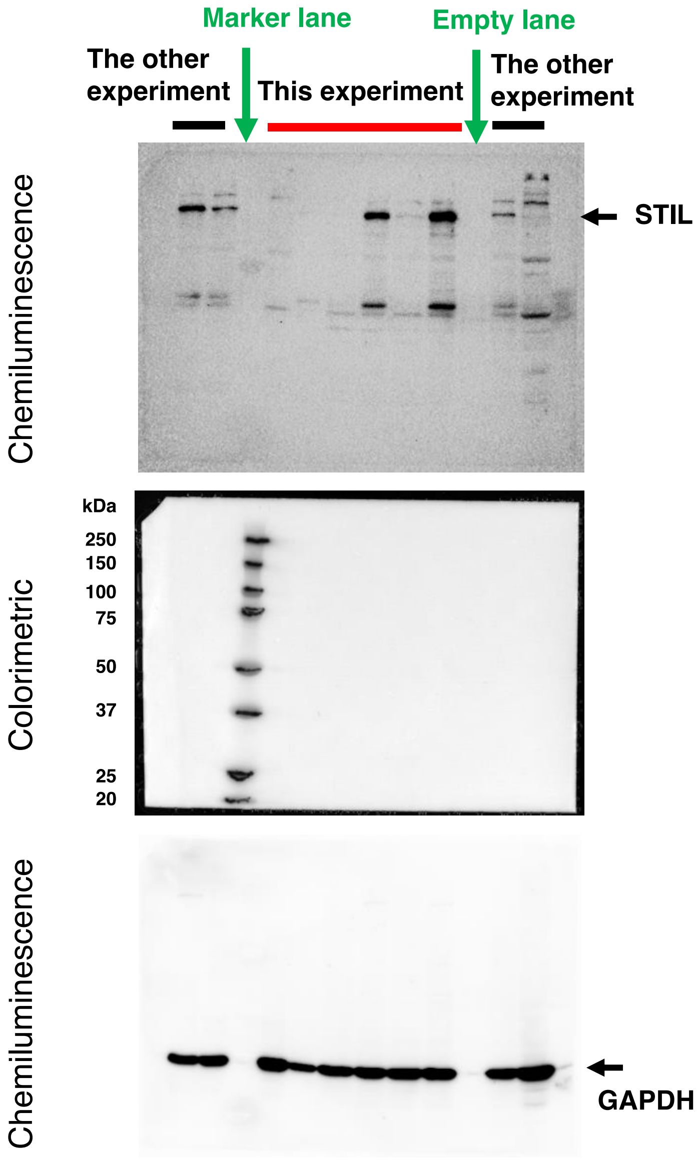

Supplementary Figure S3. Original data of Figure 2A.

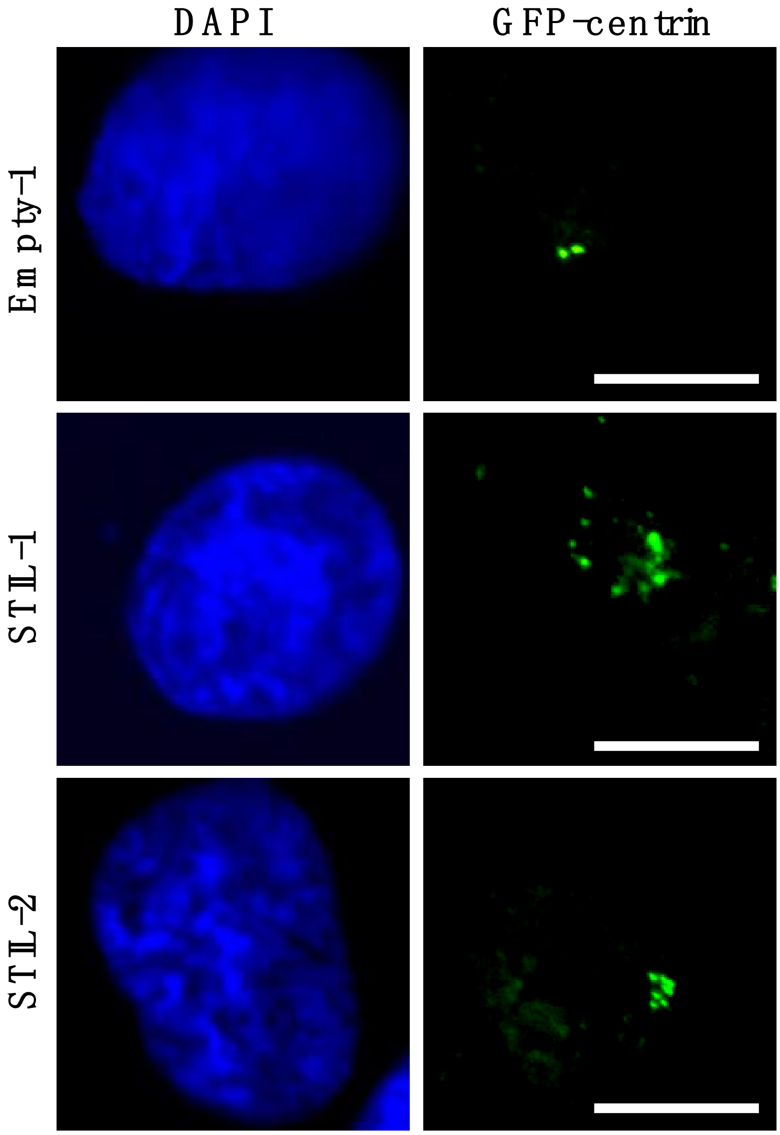

Supplementary Figure S4. Effect of STIL overexpression on centrosome amplification in H1299 NSCLC cells.

Cumate-inducible stable H1299 cell lines engineered to overexpress STIL (designated as STIL-1 and STIL-2), along with corresponding empty vector control (designated as Empty-1), were treated with cumate for 72 hours.

Following re-seeding, the cells were transfected with a GFP-centrin (CETN2) expression vector. At 24 hours post-transfection, cells were fixed with methanol and visualized via fluorescence microscopy to detect GFP-centrin. GFP-centrin was used as a marker for centriole quantification, given that centrin (CETN2) is a well-established centriole marker. A significant increase in the percentage of cells with more than four centrioles (centrin foci) was observed in STIL-overexpressing clones that was transiently transfected with GFP-centrin, while no such increase was detected in empty vector-transpoased clones that was transiently transfected with GFP-centrin. Representative fluorescence images are shown. GFP-Centrin (green) highlights centrioles, while the nucleus is counterstained with DAPI (blue). Scale bar = 10 μm.

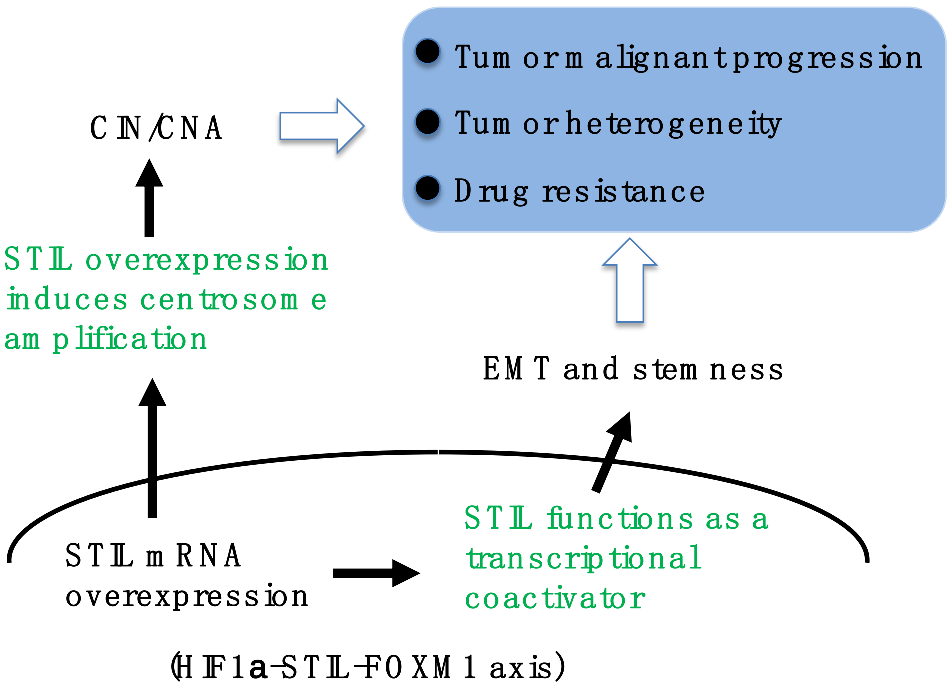

Supplementary Figure S5: A schematic representation illustrating the functional role of STIL overexpression.

The schema summarizes the findings of our current study on the effects of STIL overexpression, incorporating the results reported by Wang et al. [27].

Supplementary Table S1. Information of the primers used for the construction and validation of the FLAG-STIL/PiggyBac cumate switch inducible vector.

Primer name	Sequence (**5'->3'**)
NotI-koz-Flag-STIL-F	AAGGAAAAAAGCGGCCGCACCATGGACTAC AAGGACGACGATGACAAGGAGCCTATATAT CCTTTTGCACGGCC
NotI-STIL-R	AAGGAAAAAAGCGGCCGCTTAAAATAATTTT GGTAACTGTCTGAG
STIL-365-F	ATGGAGCCTATATATCCTTTTGCACG
STIL-700-F	AACCCCTACTGCTTCTCTTCCTG
STIL-995-F	CCAACAGCTCTGGCAAGAAACTT
STIL-1092-R	TTGCGTGTTTCATCCATGGTAAG
STIL-1099-F	ACTTTTGTTGGAATCTGATCCCA
STIL-1494-F	AGAGGTCTTCCCAAAAGTTATCTTCTG
STIL-2231-F	GCAAACACAGTTGGATCCATTC
STIL-2260-R	GACATCTTGAATGGATCCAACTGTG
STIL-2498-R	CTGACTCAGTCTTGGGTGTGTG
STIL-2650-F	AGTGCAAGCTGGAAGACAAATG
STIL-3048-F	GTGTAAGCATGTGTTTACAGACTGGAC
STIL-3457-F	CAGCCCAGAAGCAGTGATCTC
STIL-4228-R	TTAAAATAATTTTGGTAACTGTCTGAGACG

Supplementary Table S2. Chromosomal arm-level copy number status in 512 LUAD cases.

Case No.	1p	1q	2p	2q	3p	3q	4p	4q	5p	5q	6p	6q	7p	7q	8p	8q	9p	9q	10p	10q	11p	11q	12p	12q	13q	14q	15q	16p	16q	17p	17q	18p	18q	19p	19q	20p	20q	21q	22q	Xp	Xq	CNA status
1	0	1	0	0	0	0	0	0	0	0	0	0	1	0	0	0	0	0	0	0	0	0	0	0	0	0	0	0	0	0	0	0	0	-1	0	0	0	0	0	0	0	CNA-Low
2	-1	1	1	-1	-1	-1	0	0	0	0	0	-1	1	0	-1	1	1	-1	1	0	-1	-1	-1	1	-1	1	0	1	-1	0	0	1	1	-1	1	0	1	-1	-1	0	1	CNA-high
3	0	0	0	0	0	0	0	0	0	0	0	-1	0	0	-1	1	0	0	0	0	1	1	-1	0	-1	0	0	0	0	-1	1	-1	-1	0	0	0	0	0	0	0	0	CNA-Low
4	0	0	1	0	0	1	1	0	1	-1	-1	-1	1	1	-1	1	-1	-1	-1	-1	0	0	0	1	1	0	-1	1	-1	-1	1	1	-1	-1	1	1	1	0	0	0	0	CNA-high
5	0	1	1	1	0	0	0	0	1	0	0	-1	1	1	0	1	-1	-1	1	1	-1	-1	1	-1	-1	-1	0	0	0	-1	1	-1	-1	-1	0	0	1	0	0	0	0	CNA-high
6	1	1	1	1	-1	0	0	-1	1	1	1	-1	1	1	-1	1	-1	0	-1	0	0	0	-1	-1	-1	0	-1	1	1	-1	-1	0	-1	-1	-1	-1	1	1	-1	0	0	CNA-high
7	0	0	0	0	-1	1	-1	-1	-1	-1	0	0	-1	1	-1	-1	0	0	1	1	0	0	0	0	0	-1	0	0	0	0	0	-1	-1	0	0	0	0	-1	-1	0	0	CNA-Low
8	0	0	0	0	0	0	-1	0	0	-1	0	0	0	0	0	0	0	0	0	0	0	0	0	0	0	-1	0	0	0	0	0	1	-1	-1	0	0	0	0	0	-1	-1	CNA-Low
9	0	1	0	0	-1	0	-1	-1	1	0	1	-1	1	1	-1	1	1	-1	0	0	0	0	-1	0	-1	1	-1	1	1	-1	0	0	0	0	1	1	1	1	-1	0	0	CNA-high
10	0	0	0	0	0	0	0	0	0	0	0	0	0	0	0	0	0	0	0	0	0	0	0	0	0	0	0	0	0	1	1	0	0	0	0	0	0	0	0	0	0	CNA-Low
11	0	1	1	0	1	0	1	0	1	-1	1	-1	1	1	-1	1	0	1	-1	0	1	0	-1	0	0	1	-1	-1	1	0	-1	-1	-1	0	0	0	0	1	0	0	0	CNA-high
12	0	1	0	0	0	0	0	0	1	0	0	0	0	1	-1	0	-1	0	0	0	0	0	0	0	0	0	0	0	0	1	1	0	0	-1	0	0	0	0	-1	0	0	CNA-Low
13	1	1	1	0	-1	1	0	0	1	0	1	0	-1	-1	-1	1	-1	-1	-1	-1	-1	-1	1	1	-1	-1	0	0	0	-1	1	1	1	-1	0	1	1	1	1	0	0	CNA-high
14	0	1	0	0	-1	-1	0	0	0	0	1	-1	0	0	-1	0	-1	-1	0	0	0	0	0	0	0	0	0	0	0	0	0	0	0	-1	0	0	0	-1	-1	-1	-1	CNA-Low
15	0	1	0	0	0	0	0	0	1	0	1	-1	1	0	-1	1	-1	-1	1	1	-1	1	0	0	-1	0	-1	1	0	0	1	-1	-1	-1	0	-1	1	-1	-1	-1	1	CNA-high
16	-1	1	0	0	-1	-1	0	0	1	1	1	-1	0	1	0	0	-1	-1	0	0	0	0	-1	0	-1	0	-1	1	1	1	1	0	0	0	-1	1	1	-1	-1	0	0	CNA-high
17	1	1	0	0	-1	1	0	0	1	-1	1	-1	1	1	-1	1	0	0	0	0	0	0	1	0	-1	1	-1	0	0	-1	1	-1	-1	-1	-1	1	1	1	-1	-1	1	CNA-high
18	0	0	0	0	0	0	0	0	0	0	0	-1	0	0	0	0	0	0	0	0	0	0	-1	0	0	0	0	0	0	0	0	0	0	0	0	0	0	0	0	0	0	CNA-Low
19	1	1	0	0	-1	0	0	0	1	0	1	0	1	1	1	1	-1	-1	-1	-1	0	0	1	1	-1	0	0	0	0	-1	1	0	0	-1	-1	-1	0	0	1	0	0	CNA-high
20	1	1	1	-1	1	1	-1	-1	1	-1	1	-1	1	1	1	1	0	0	1	1	0	0	-1	0	-1	1	-1	-1	-1	0	1	-1	-1	0	-1	1	1	-1	0	-1	1	CNA-high
21	0	1	-1	-1	-1	0	1	1	1	1	1	1	1	0	-1	1	0	0	-1	-1	0	0	-1	-1	1	1	-1	-1	-1	-1	1	1	0	-1	-1	1	1	0	-1	1	1	CNA-high
22	0	0	1	1	0	0	1	0	1	0	0	0	0	0	1	1	-1	0	1	-1	-1	0	1	1	0	0	-1	0	0	1	1	0	0	0	1	1	1	-1	-1	0	0	CNA-Low
23	0	1	1	1	-1	-1	0	0	1	-1	0	0	1	0	-1	1	-1	-1	-1	1	-1	1	-1	-1	-1	-1	-1	0	-1	-1	1	1	-1	-1	-1	-1	-1	0	0	0	1	CNA-high
24	-1	0	0	0	-1	0	1	1	1	0	0	-1	1	0	-1	0	0	-1	-1	0	-1	-1	-1	0	0	0	-1	0	0	-1	1	0	-1	-1	-1	-1	0	1	1	0	0	CNA-high
25	0	0	0	0	0	0	0	0	1	1	0	-1	1	1	0	1	0	0	0	0	1	1	0	0	0	1	0	0	0	0	0	0	0	0	0	1	1	0	0	-1	1	CNA-Low
26	0	1	0	0	0	0	0	0	0	0	1	-1	0	0	0	0	0	1	0	0	0	0	0	0	0	0	0	0	0	0	0	0	0	0	0	0	0	0	0	-1	1	CNA-Low
27	0	1	0	0	0	0	0	0	1	1	1	0	0	0	0	0	0	0	0	0	1	1	0	0	1	0	0	1	1	-1	1	0	0	0	0	0	0	0	0	1	1	CNA-Low
28	0	0	0	0	0	0	0	0	1	1	0	-1	0	0	0	0	-1	0	0	0	0	0	0	0	0	0	0	0	0	0	0	-1	-1	0	0	-1	0	0	0	0	0	CNA-Low
29	1	1	0	0	-1	-1	0	0	1	1	0	0	1	0	-1	0	-1	-1	0	0	0	0	-1	-1	-1	0	0	1	1	-1	0	-1	-1	-1	0	0	0	1	-1	-1	0	CNA-high
30	0	1	0	0	0	0	0	0	1	0	0	-1	1	1	1	1	0	0	1	1	0	1	0	0	-1	1	0	1	-1	-1	1	0	0	0	0	1	1	-1	0	0	0	CNA-Low
31	0	1	1	0	0	1	0	0	1	0	0	0	1	1	1	1	0	0	0	0	0	1	0	0	0	0	0	0	0	0	1	1	1	0	0	0	0	0	0	1	1	CNA-Low
32	0	0	0	0	0	0	1	1	1	1	1	1	1	0	-1	0	-1	0	-1	-1	0	0	0	0	0	0	-1	1	0	0	0	0	0	0	1	1	1	0	0	0	0	CNA-Low
33	0	0	0	-1	1	1	0	1	0	-1	0	0	1	1	-1	1	0	0	1	-1	-1	1	1	1	-1	-1	-1	1	-1	-1	1	-1	-1	-1	0	-1	1	0	0	-1	1	CNA-high
34	0	0	1	1	0	0	0	0	1	0	0	0	1	1	0	1	1	0	0	0	0	0	1	1	0	0	-1	1	1	0	0	0	0	0	0	-1	1	0	-1	0	0	CNA-Low
35	0	0	0	0	0	0	0	0	1	1	0	0	1	1	0	0	0	0	0	0	1	1	1	0	0	1	0	1	1	0	0	0	0	0	0	0	0	0	1	0	0	CNA-Low
36	0	0	0	0	0	0	-1	1	1	0	-1	-1	1	0	-1	0	-1	-1	0	0	0	0	0	0	-1	0	-1	-1	-1	-1	0	-1	-1	0	1	-1	-1	-1	-1	0	0	CNA-high
37	0	1	0	1	0	0	0	0	0	0	1	-1	0	0	1	1	0	0	1	1	0	0	0	0	-1	-1	0	1	1	-1	0	0	0	-1	0	1	1	0	0	1	1	CNA-Low
38	0	1	0	0	-1	0	0	0	0	0	0	-1	1	1	-1	0	0	0	0	0	0	0	0	0	0	0	-1	1	-1	-1	1	-1	-1	-1	-1	-1	0	-1	0	-1	1	CNA-Low
39	0	1	0	1	-1	-1	-1	-1	1	-1	-1	0	0	1	0	0	-1	0	0	0	0	0	0	1	-1	0	0	-1	1	0	1	1	1	-1	1	0	1	0	1	0	0	CNA-high
40	0	0	0	0	0	0	0	0	0	0	0	0	0	0	-1	-1	-1	-1	-1	-1	0	0	0	0	0	1	-1	0	0	-1	-1	-1	-1	-1	-1	-1	-1	1	1	0	0	CNA-Low

(Continued)

41	-1	1	1	0	-1	-1	0	0	1	-1	0	-1	1	1	0	0	0	0	0	0	0	0	1	0	0	1	-1	0	0	1	1	0	0	0	0	0	1	1	1	0	1	CNA-Low
42	0	1	0	0	0	0	0	0	1	-1	0	-1	0	0	0	0	0	0	0	0	0	0	0	0	0	0	-1	0	-1	0	-1	-1	0	0	-1	-1	0	0	-1	-1	-1	-1	CNA-Low
43	0	1	0	1	0	0	-1	-1	1	-1	0	-1	1	1	1	1	-1	0	1	0	0	0	-1	0	-1	-1	0	0	-1	0	0	-1	-1	0	0	0	0	1	0	-1	1	CNA-high
44	0	1	-1	1	1	1	-1	0	1	-1	1	0	1	1	-1	0	1	0	1	-1	-1	1	0	0	0	0	-1	0	0	-1	1	1	0	-1	0	1	0	0	1	0	0	CNA-high
45	-1	1	1	1	-1	-1	-1	-1	1	0	-1	-1	1	-1	0	-1	-1	-1	1	1	1	1	1	1	0	1	1	-1	-1	-1	1	1	1	-1	1	-1	-1	1	-1	0	0	CNA-high
46	-1	1	1	1	0	0	-1	0	0	-1	0	0	1	-1	1	1	0	0	1	1	0	0	1	1	-1	1	-1	0	0	-1	1	-1	-1	0	0	1	-1	-1	0	0	0	CNA-high
47	-1	1	1	1	1	1	-1	-1	1	-1	0	0	0	0	1	1	-1	-1	1	-1	0	0	0	0	-1	1	-1	0	-1	0	1	1	-1	-1	1	1	0	-1	-1	0	0	CNA-high
48	1	0	0	0	1	1	0	0	0	-1	0	0	-1	1	-1	0	1	1	1	0	-1	-1	-1	0	0	-1	0	-1	-1	-1	1	1	-1	-1	0	0	0	-1	-1	-1	0	CNA-high
49	0	0	0	0	0	0	0	0	0	0	0	0	0	0	-1	0	0	0	0	0	0	-1	0	0	0	0	0	0	0	-1	0	0	-1	-1	0	0	0	0	-1	0	0	CNA-Low
50	0	0	0	0	-1	-1	0	0	1	-1	1	-1	0	0	-1	0	1	-1	0	0	0	1	0	0	0	0	-1	0	0	0	0	0	0	-1	0	1	1	1	0	0	0	CNA-Low
51	1	1	0	0	0	0	1	1	1	0	-1	-1	1	0	-1	0	0	0	-1	-1	1	1	-1	-1	0	-1	-1	1	-1	-1	1	1	-1	0	0	1	1	1	0	0	0	CNA-high
52	0	1	0	0	-1	-1	0	0	0	0	1	0	0	0	0	0	0	0	0	0	0	0	0	0	0	0	0	0	0	0	0	0	0	0	-1	1	0	0	0	0	0	CNA-Low
53	0	1	0	0	0	0	0	0	1	1	0	-1	1	1	-1	1	-1	-1	0	0	0	0	0	0	-1	0	-1	1	-1	-1	1	-1	-1	0	0	0	0	1	1	0	0	CNA-Low
54	1	1	0	1	-1	0	-1	-1	1	-1	0	-1	0	1	1	1	0	0	0	0	1	1	0	0	-1	1	-1	0	1	-1	0	-1	1	-1	-1	1	1	-1	-1	0	1	CNA-high
55	1	1	1	0	-1	0	0	0	0	-1	0	-1	1	0	0	1	-1	0	-1	1	1	1	1	0	-1	1	0	1	1	-1	0	-1	-1	-1	-1	-1	0	-1	1	0	0	CNA-high
56	0	1	1	0	0	1	-1	-1	1	0	1	0	0	0	-1	-1	-1	0	1	-1	-1	-1	0	0	-1	0	-1	0	-1	0	0	1	-1	0	0	0	1	1	0	0	0	CNA-high
57	1	1	1	1	0	0	0	0	1	0	1	1	1	1	-1	0	-1	-1	-1	-1	-1	0	-1	-1	0	1	-1	1	0	-1	1	-1	-1	-1	-1	0	0	1	0	0	0	CNA-high
58	1	0	0	0	0	0	0	0	1	1	1	-1	1	1	-1	1	0	0	0	0	1	1	0	0	-1	1	0	1	1	-1	0	-1	-1	0	0	-1	1	1	0	0	1	CNA-high
59	0	0	0	0	-1	0	0	0	1	-1	0	-1	0	0	0	0	-1	-1	0	0	0	0	0	0	0	-1	-1	0	0	0	0	0	0	-1	0	0	0	0	0	0	-1	CNA-Low
60	1	1	1	1	-1	-1	-1	0	-1	-1	1	1	1	-1	1	0	-1	-1	-1	-1	0	0	1	1	1	-1	1	-1	-1	1	1	-1	-1	-1	0	0	0	1	1	0	0	CNA-high
61	0	1	1	0	-1	1	-1	-1	1	-1	0	0	0	0	-1	-1	-1	-1	0	0	-1	0	-1	0	0	0	0	0	-1	-1	1	-1	-1	-1	1	-1	-1	1	1	0	0	CNA-high
62	1	1	0	0	-1	0	0	0	1	0	0	-1	0	0	0	-1	0	0	0	0	0	0	0	0	-1	0	-1	1	1	-1	1	0	0	-1	0	0	0	0	-1	-1	0	CNA-Low
63	-1	-1	0	0	-1	0	0	0	0	0	-1	-1	1	1	-1	0	0	0	0	0	0	0	-1	-1	0	0	0	-1	-1	-1	1	0	0	-1	-1	-1	-1	-1	0	0	0	CNA-Low
64	0	1	0	0	0	1	1	0	0	-1	1	0	1	1	-1	1	-1	1	-1	0	-1	1	1	0	0	1	-1	-1	0	-1	0	-1	-1	-1	-1	0	1	0	0	-1	1	CNA-high
65	1	1	0	0	0	0	0	0	0	0	0	0	0	0	0	0	0	0	0	0	0	0	0	0	0	0	0	0	0	0	0	0	0	0	0	0	0	0	0	0	0	CNA-Low
66	-1	1	0	0	0	0	-1	-1	1	1	-1	-1	0	0	1	1	1	1	1	1	0	0	-1	1	-1	0	-1	0	0	-1	-1	0	-1	-1	-1	-1	-1	-1	-1	0	0	CNA-high
67	1	1	1	1	-1	1	0	0	0	0	0	0	1	-1	0	1	-1	-1	0	0	-1	-1	1	1	0	0	1	0	0	0	0	-1	-1	-1	0	-1	-1	-1	0	0	0	CNA-high
68	0	0	0	0	0	0	-1	0	0	-1	1	-1	1	1	0	1	0	1	0	0	0	0	1	0	0	1	0	0	0	0	0	0	0	0	0	1	1	0	-1	1	-1	CNA-Low
69	-1	0	0	0	-1	-1	0	-1	0	0	0	0	1	0	0	0	0	0	0	0	0	0	0	0	0	1	-1	0	0	0	0	0	0	0	0	1	1	-1	0	0	0	CNA-Low
70	1	1	1	0	-1	-1	0	0	1	1	0	1	1	-1	-1	0	-1	0	-1	-1	1	1	1	0	-1	-1	-1	1	1	-1	1	-1	-1	-1	0	-1	0	1	-1	0	-1	CNA-high
71	0	1	0	0	0	0	0	0	0	1	1	-1	0	0	0	1	-1	-1	0	1	-1	1	0	0	-1	0	-1	0	0	1	1	0	0	0	0	1	1	0	-1	0	0	CNA-Low
72	1	1	0	0	0	-1	1	1	0	0	0	0	1	1	0	1	-1	-1	0	0	1	1	1	1	-1	0	0	1	-1	-1	1	0	-1	0	0	-1	1	0	1	1	1	CNA-high
73	1	1	-1	-1	-1	0	-1	-1	1	-1	1	0	1	1	-1	1	0	0	1	-1	1	1	-1	-1	-1	1	1	1	0	-1	1	-1	1	-1	-1	0	1	1	-1	-1	1	CNA-high
74	-1	1	0	0	1	1	0	0	1	0	0	0	-1	-1	0	1	1	0	-1	-1	0	0	-1	1	-1	1	-1	0	0	-1	1	0	0	-1	-1	-1	0	-1	-1	0	0	CNA-high
75	0	1	1	-1	-1	0	1	-1	1	-1	0	0	0	0	0	0	0	-1	1	-1	1	1	1	-1	-1	0	0	0	-1	0	0	1	-1	-1	-1	1	1	0	-1	0	0	CNA-high
76	1	1	1	0	-1	0	0	-1	0	-1	1	-1	1	0	0	1	1	-1	0	0	0	0	0	0	-1	0	0	-1	0	-1	0	1	1	-1	-1	1	0	0	-1	0	0	CNA-high
77	0	1	1	1	-1	1	0	0	0	0	1	0	1	1	0	0	-1	-1	-1	-1	0	0	-1	0	-1	1	-1	0	0	-1	1	0	0	0	0	-1	1	0	0	0	0	CNA-Low
78	0	0	1	1	0	0	1	0	1	0	0	0	0	1	-1	1	0	1	1	0	-1	0	-1	1	-1	0	-1	0	0	-1	1	-1	-1	-1	0	1	1	0	-1	0	0	CNA-high
79	-1	1	0	0	0	0	0	0	1	1	1	-1	1	0	-1	1	0	0	0	0	0	0	-1	0	-1	0	-1	-1	-1	-1	0	1	-1	0	0	0	0	-1	-1	0	1	CNA-Low
80	0	0	1	0	-1	-1	-1	-1	0	0	1	1	1	-1	-1	1	-1	-1	0	-1	-1	-1	1	0	1	0	-1	1	1	-1	0	0	0	0	0	1	1	1	1	0	0	CNA-high

(Continued)

81	0	0	0	0	-1	0	-1	-1	0	-1	-1	-1	0	0	-1	0	0	0	0	0	-1	0	0	0	0	0	0	0	0	-1	0	0	-1	-1	0	0	0	-1	-1	-1	-1	CNA-Low
82	1	1	0	0	0	0	0	0	1	-1	0	0	0	0	-1	1	0	0	0	0	0	0	0	0	0	0	0	-1	0	0	0	0	0	0	0	1	1	1	0	0	0	0	CNA-Low
83	0	1	1	1	0	0	0	0	1	0	1	0	0	0	1	1	0	0	1	1	0	0	0	0	0	1	0	0	0	0	0	1	1	0	0	1	1	0	0	1	1	CNA-Low
84	0	0	0	0	1	0	-1	-1	0	0	-1	-1	1	1	1	1	-1	0	0	-1	1	0	0	1	-1	1	-1	-1	-1	-1	0	1	-1	-1	0	0	0	-1	-1	0	0	CNA-high
85	1	1	0	0	1	1	0	0	1	0	1	-1	1	1	0	1	1	1	1	1	-1	-1	0	0	0	0	0	0	0	-1	0	-1	-1	-1	0	1	1	-1	-1	0	0	CNA-high
86	0	0	0	1	-1	-1	0	0	0	-1	0	0	1	1	-1	0	-1	-1	0	0	0	0	0	0	-1	1	0	0	1	-1	0	-1	-1	-1	-1	-1	0	0	-1	0	0	CNA-Low
87	0	0	0	0	0	0	0	0	0	0	0	0	1	1	1	1	0	0	1	1	0	0	1	1	-1	0	0	-1	-1	-1	1	0	0	-1	0	-1	1	-1	0	0	0	CNA-Low
88	1	1	0	0	0	0	0	0	1	-1	0	0	1	1	-1	1	0	0	0	-1	1	1	-1	-1	0	0	0	-1	-1	-1	1	-1	-1	0	0	1	1	0	0	0	0	CNA-high
89	1	1	0	0	0	0	0	0	1	1	0	0	1	1	-1	0	-1	-1	-1	-1	0	0	1	0	-1	0	-1	1	1	-1	0	1	-1	0	0	-1	0	1	-1	0	0	CNA-high
90	1	1	1	0	0	0	0	0	1	0	0	-1	1	1	-1	1	-1	-1	-1	-1	1	1	1	-1	-1	1	-1	0	0	-1	1	0	0	-1	0	0	0	0	1	0	0	CNA-high
91	1	1	0	0	-1	-1	0	0	0	0	0	0	1	-1	-1	1	-1	-1	-1	-1	0	0	0	-1	-1	0	-1	0	0	-1	1	0	-1	-1	-1	0	0	0	1	0	0	CNA-high
92	0	1	1	1	-1	-1	0	0	1	1	-1	-1	0	0	0	0	0	0	0	0	0	0	0	0	0	1	-1	0	0	0	0	0	0	0	0	0	0	-1	0	0	0	CNA-Low
93	0	1	0	0	0	1	0	0	0	0	0	0	0	0	0	0	0	0	0	0	0	0	1	-1	0	0	0	0	0	-1	0	0	0	0	0	-1	0	0	0	0	0	CNA-Low
94	0	0	0	0	-1	0	-1	0	1	-1	1	-1	1	1	-1	1	-1	-1	0	0	0	0	1	0	-1	0	0	0	0	-1	0	-1	-1	0	0	0	0	-1	-1	-1	1	CNA-high
95	-1	-1	-1	-1	0	0	0	0	0	0	0	0	0	0	0	0	-1	-1	-1	-1	0	0	-1	0	-1	0	0	-1	-1	0	0	0	0	0	0	0	0	-1	0	0	0	CNA-Low
96	0	1	0	0	0	0	0	0	1	0	0	0	1	1	0	0	0	1	0	0	0	0	1	1	-1	0	0	0	0	0	0	0	0	0	0	0	0	0	0	-1	1	CNA-Low
97	0	0	1	1	-1	0	1	-1	1	0	1	-1	1	-1	-1	0	1	1	1	1	0	0	0	-1	1	-1	-1	1	-1	-1	0	-1	-1	-1	0	-1	1	1	-1	0	0	CNA-high
98	0	1	1	1	0	0	0	0	0	0	-1	-1	1	-1	-1	-1	-1	-1	0	0	-1	-1	0	0	0	1	-1	1	1	-1	-1	-1	-1	-1	0	0	0	0	0	-1	1	CNA-high
99	-1	1	1	1	-1	-1	1	-1	0	-1	0	-1	0	0	1	1	-1	-1	1	1	0	0	1	1	1	-1	0	0	1	0	0	-1	-1	-1	0	1	0	-1	-1	0	0	CNA-high
100	0	0	0	0	0	0	1	1	0	0	0	0	0	0	0	0	0	0	0	0	0	0	0	0	0	0	0	0	0	0	0	0	0	0	0	0	0	0	0	0	0	CNA-Low
101	-1	1	-1	-1	-1	-1	0	-1	1	0	0	0	0	-1	0	1	0	1	0	-1	0	0	0	0	-1	1	-1	0	-1	-1	1	0	-1	-1	-1	1	-1	-1	0	-1	1	CNA-high
102	0	0	0	0	0	0	0	0	0	0	0	-1	0	0	1	1	0	0	0	0	0	0	0	0	0	0	0	0	0	0	0	0	0	0	0	0	0	0	0	0	0	CNA-Low
103	-1	1	0	1	-1	-1	0	0	1	-1	1	0	0	0	0	1	1	-1	1	0	0	1	1	0	0	1	-1	0	0	-1	0	0	0	-1	0	-1	0	-1	-1	0	0	CNA-high
104	-1	0	0	1	-1	1	0	0	1	-1	1	1	0	0	-1	0	0	0	-1	-1	1	1	1	0	0	0	0	0	-1	-1	1	-1	-1	0	0	-1	1	0	-1	0	0	CNA-high
105	0	0	0	0	-1	1	-1	-1	1	0	0	0	0	0	0	0	-1	-1	0	0	0	0	0	0	0	-1	0	0	0	0	0	-1	-1	-1	-1	0	0	0	-1	0	0	CNA-Low
106	1	1	0	1	-1	1	-1	-1	1	-1	0	0	-1	-1	1	1	-1	0	-1	0	0	1	-1	1	-1	1	-1	1	1	-1	0	1	-1	0	1	1	1	-1	-1	0	0	CNA-high
107	1	1	1	1	-1	-1	-1	-1	0	0	1	1	1	1	-1	1	-1	-1	0	-1	-1	-1	-1	-1	0	1	-1	-1	-1	-1	1	-1	-1	0	0	1	1	0	1	0	0	CNA-high
108	0	0	1	0	-1	0	0	1	1	0	1	0	1	1	0	0	-1	-1	0	0	1	1	1	0	0	-1	0	0	0	0	1	0	0	0	-1	-1	0	0	0	0	0	CNA-Low
109	0	1	0	0	0	0	0	0	0	0	0	-1	0	0	0	0	-1	0	0	0	0	0	0	0	0	0	0	0	0	0	0	0	0	-1	0	0	0	0	0	0	0	CNA-Low
110	0	1	0	0	-1	-1	0	0	1	-1	0	0	-1	1	-1	0	1	-1	0	0	-1	1	0	-1	-1	-1	-1	0	0	-1	1	-1	0	-1	-1	-1	1	0	-1	0	0	CNA-high
111	-1	0	0	1	-1	1	-1	-1	1	-1	-1	-1	0	1	-1	0	0	0	-1	-1	1	0	1	-1	0	0	0	0	0	0	1	0	-1	-1	1	-1	1	-1	0	-1	1	CNA-high
112	0	1	0	0	0	0	0	0	1	-1	0	0	0	0	0	0	0	0	0	0	0	0	0	0	0	0	0	0	0	0	0	0	0	0	0	0	0	0	0	0	0	CNA-Low
113	0	0	0	0	-1	0	0	-1	1	-1	0	-1	1	0	-1	1	0	-1	0	0	-1	0	1	1	-1	-1	0	0	-1	0	0	0	1	-1	-1	1	0	-1	-1	0	0	CNA-high
114	-1	0	-1	0	0	0	-1	-1	1	-1	1	0	0	0	1	1	-1	-1	1	0	0	0	0	0	-1	1	-1	0	0	-1	0	1	0	-1	0	0	0	-1	-1	-1	1	CNA-high
115	1	1	1	1	0	0	0	0	1	-1	0	0	0	0	0	0	-1	0	-1	-1	0	0	0	0	1	-1	-1	1	-1	-1	1	-1	-1	0	0	0	0	0	-1	-1	1	CNA-high
116	-1	1	1	1	0	-1	0	0	0	0	1	-1	1	1	-1	1	-1	-1	-1	0	0	0	-1	-1	0	-1	-1	0	0	0	0	-1	-1	0	-1	-1	0	0	-1	0	0	CNA-high
117	0	0	0	0	-1	0	0	0	1	-1	0	0	0	0	-1	0	-1	-1	0	0	-1	0	0	0	-1	0	0	0	0	0	0	-1	0	-1	-1	0	0	0	-1	-1	-1	CNA-Low
118	1	1	1	1	-1	0	1	-1	1	0	1	0	0	0	-1	-1	-1	-1	0	0	-1	0	-1	0	-1	0	0	1	0	-1	0	-1	-1	0	0	0	0	0	0	0	0	CNA-Low
119	0	1	0	0	0	0	-1	-1	1	0	0	0	0	0	-1	0	0	0	0	0	0	0	0	0	0	0	-1	0	0	-1	0	-1	-1	0	0	1	0	0	-1	0	0	CNA-Low
120	0	1	0	0	1	1	0	1	1	1	0	0	1	1	1	1	-1	-1	0	0	0	0	0	0	0	0	-1	0	-1	-1	1	1	-1	0	0	-1	0	0	-1	0	0	CNA-Low

(Continued)

121	1	1	1	0	1	1	1	0	0	0	0	0	1	1	0	0	0	-1	0	0	-1	-1	0	1	-1	1	-1	0	1	-1	1	0	-1	-1	0	-1	0	0	-1	0	0	CNA-high
122	-1	0	0	0	-1	1	0	0	0	-1	0	-1	0	0	0	1	0	-1	0	0	0	0	1	1	-1	-1	-1	1	-1	-1	1	-1	-1	-1	-1	0	0	-1	-1	-1	1	CNA-high
123	1	1	0	0	-1	0	0	-1	1	1	1	0	1	1	0	0	-1	-1	0	-1	-1	1	-1	0	-1	0	0	0	0	-1	1	1	-1	-1	0	0	0	1	0	0	0	CNA-high
124	-1	1	0	0	-1	-1	0	-1	0	0	0	0	0	-1	1	1	-1	-1	0	0	0	-1	1	1	0	1	1	0	0	0	0	1	1	-1	1	-1	0	-1	0	0	0	CNA-high
125	1	1	1	1	0	0	0	0	1	-1	1	-1	0	0	1	1	-1	-1	1	1	0	0	0	0	1	1	1	0	0	1	1	0	0	-1	-1	0	0	0	0	0	1	CNA-high
126	0	1	0	1	-1	0	1	0	0	-1	0	0	1	0	-1	-1	0	-1	-1	0	0	0	1	0	0	-1	0	0	-1	-1	0	1	-1	-1	1	0	0	0	1	0	0	CNA-Low
127	0	1	0	0	0	0	0	0	0	0	0	-1	0	0	-1	1	0	0	0	0	0	0	0	0	0	0	0	1	-1	0	0	0	0	0	0	1	0	0	-1	0	0	CNA-Low
128	-1	1	-1	-1	0	0	1	0	1	1	-1	-1	0	0	-1	1	0	0	0	0	0	0	-1	-1	0	0	-1	0	0	0	0	0	0	-1	-1	0	0	-1	0	0	0	CNA-Low
129	-1	1	0	1	0	1	1	0	1	1	-1	0	1	0	0	0	0	0	1	0	-1	1	1	0	-1	0	1	1	1	-1	1	0	0	1	1	1	1	-1	1	-1	1	CNA-high
130	1	0	0	0	0	1	1	0	1	0	1	0	1	0	-1	0	0	0	1	0	0	1	0	0	0	0	0	0	-1	0	0	1	-1	0	0	1	1	1	0	1	1	CNA-Low
131	0	1	0	0	0	0	0	0	0	0	0	-1	0	0	0	0	-1	0	0	0	0	0	0	0	0	0	0	1	0	0	0	0	0	-1	0	0	0	0	0	0	0	CNA-Low
132	0	1	0	0	0	0	0	0	0	0	1	-1	0	0	0	1	0	0	0	0	0	0	0	0	0	0	-1	0	0	0	0	0	0	0	0	-1	-1	0	0	0	0	CNA-Low
133	-1	1	1	0	-1	0	0	0	1	-1	1	-1	0	0	-1	1	0	-1	1	1	0	0	1	0	0	1	-1	0	-1	-1	1	0	0	-1	0	-1	1	0	-1	0	0	CNA-high
134	-1	1	1	1	-1	-1	0	0	1	-1	0	0	1	1	0	0	-1	1	1	1	-1	-1	0	0	-1	0	0	0	0	0	0	0	0	0	0	0	-1	0	0	0	0	CNA-Low
135	0	1	1	0	0	-1	0	0	0	-1	1	0	1	0	0	1	0	0	1	1	0	0	0	0	-1	1	0	0	0	0	1	0	-1	0	0	0	0	0	0	0	0	CNA-Low
136	0	1	-1	1	-1	0	1	0	1	-1	1	1	-1	1	0	1	0	0	0	0	0	0	1	1	1	0	1	0	-1	1	1	0	0	-1	0	1	1	-1	0	1	-1	CNA-high
137	0	1	0	0	0	0	0	0	1	-1	-1	-1	1	1	0	0	-1	-1	-1	-1	1	1	0	0	0	0	-1	1	-1	0	1	-1	-1	0	0	0	0	0	0	0	0	CNA-Low
138	1	1	0	0	-1	-1	0	0	1	1	1	1	0	0	-1	-1	0	0	-1	-1	0	0	-1	-1	-1	-1	-1	1	-1	0	0	1	0	0	0	1	1	1	-1	0	0	CNA-high
139	0	1	1	1	0	0	0	0	1	1	-1	-1	0	0	1	1	0	0	0	0	0	0	-1	-1	1	-1	0	1	1	0	0	1	0	0	0	0	0	-1	0	0	0	CNA-Low
140	0	0	0	0	0	0	0	0	0	0	0	0	0	0	0	0	0	0	0	0	0	0	0	0	0	0	0	0	0	0	0	-1	-1	0	0	0	0	0	-1	0	0	CNA-Low
141	1	1	0	0	0	0	0	0	1	0	1	0	1	1	1	1	1	1	1	1	-1	1	0	0	0	0	1	0	1	0	1	0	0	0	0	1	1	1	0	0	0	CNA-Low
142	-1	1	0	-1	-1	-1	0	0	1	0	1	-1	1	1	-1	1	1	0	1	1	1	0	-1	-1	-1	0	0	1	-1	-1	1	1	0	1	1	0	0	0	-1	-1	1	CNA-high
143	1	1	0	0	0	0	0	0	1	0	-1	0	0	0	-1	0	0	0	0	0	0	0	-1	-1	-1	0	-1	0	0	-1	0	0	0	-1	-1	-1	-1	0	-1	0	0	CNA-Low
144	0	1	0	0	0	0	0	0	1	1	0	-1	0	0	0	0	0	0	0	0	0	0	0	0	0	0	0	0	0	0	0	0	0	0	0	0	0	0	0	0	0	CNA-Low
145	0	1	0	0	-1	1	-1	-1	1	-1	0	0	1	1	-1	-1	1	-1	0	0	-1	1	1	1	1	1	-1	-1	-1	-1	1	0	1	-1	1	1	1	-1	0	0	0	CNA-high
146	0	0	0	0	0	0	1	1	0	0	0	0	1	1	0	0	0	0	0	0	1	1	0	0	0	0	-1	-1	-1	-1	1	0	0	0	0	0	0	-1	0	0	0	CNA-Low
147	0	1	0	1	0	0	0	0	1	-1	1	0	1	0	-1	1	0	0	1	-1	-1	1	-1	-1	-1	1	-1	1	0	-1	1	1	-1	0	0	1	1	-1	1	0	0	CNA-high
148	0	0	1	0	0	0	-1	-1	1	-1	1	0	1	0	0	1	-1	-1	0	0	-1	0	1	1	-1	0	-1	0	0	-1	1	0	-1	-1	1	1	1	-1	-1	0	0	CNA-high
149	1	1	0	0	0	0	0	0	1	0	0	0	1	1	1	1	-1	0	-1	-1	1	1	1	1	-1	-1	0	1	-1	0	0	-1	0	-1	1	1	1	0	0	0	0	CNA-high
150	0	1	1	1	-1	1	0	0	1	-1	1	-1	-1	-1	-1	1	0	-1	-1	-1	1	0	1	1	-1	0	-1	0	0	-1	1	0	-1	-1	-1	0	1	-1	1	1	0	CNA-high
151	0	1	0	0	-1	-1	0	0	0	0	1	0	0	0	1	1	-1	1	1	1	-1	-1	-1	-1	1	-1	0	0	0	0	0	0	0	-1	-1	1	1	0	-1	0	0	CNA-high
152	0	1	0	0	-1	-1	1	0	1	1	0	0	0	0	-1	1	0	0	1	0	0	0	1	0	0	0	-1	0	0	0	0	1	-1	0	0	1	1	0	0	0	0	CNA-Low
153	0	1	1	0	-1	0	1	0	1	-1	1	0	1	-1	-1	0	0	0	-1	1	-1	1	0	0	1	0	0	-1	0	-1	0	-1	-1	-1	1	-1	-1	0	-1	-1	0	CNA-high
154	0	1	0	0	-1	0	-1	0	1	-1	-1	-1	0	0	-1	0	0	0	0	0	-1	-1	0	0	1	0	-1	0	0	-1	0	-1	-1	0	0	0	0	1	0	-1	0	CNA-Low
155	1	1	1	1	-1	0	0	0	0	-1	0	-1	1	1	-1	0	-1	1	0	0	-1	0	1	0	-1	0	-1	-1	-1	-1	1	-1	-1	-1	0	0	0	-1	-1	0	-1	CNA-high
156	0	1	0	0	-1	1	0	-1	1	-1	0	0	1	1	-1	0	-1	-1	-1	-1	-1	0	0	0	-1	0	-1	1	-1	-1	0	1	-1	0	-1	0	0	-1	0	0	0	CNA-high
157	1	1	0	0	0	0	1	-1	0	-1	0	0	1	1	-1	0	-1	-1	-1	-1	0	0	-1	1	-1	0	-1	1	0	-1	0	0	-1	-1	-1	-1	1	0	-1	0	0	CNA-high
158	1	1	0	1	0	0	0	0	0	0	1	-1	0	0	0	0	0	0	0	0	0	0	0	0	0	0	0	0	0	0	0	0	0	-1	0	0	0	0	0	0	0	CNA-Low
159	0	0	1	1	0	0	1	1	1	1	0	0	1	1	0	0	1	0	0	0	1	1	0	0	0	0	0	1	1	0	0	0	0	0	0	1	1	1	-1	1	1	CNA-Low
160	0	1	0	0	0	0	0	0	1	0	0	0	0	0	0	0	0	0	0	0	0	0	0	0	0	0	0	0	0	0	0	0	0	-1	0	0	0	0	0	-1	-1	CNA-Low

(Continued)

161	1	1	0	1	-1	-1	1	-1	0	-1	0	0	-1	0	-1	1	-1	-1	0	0	0	0	1	0	1	0	-1	-1	-1	1	1	-1	1	-1	-1	1	0	-1	-1	0	0	CNA-high
162	1	1	0	0	-1	-1	1	-1	1	-1	-1	-1	1	0	1	1	0	0	0	-1	-1	1	-1	-1	0	-1	-1	1	-1	-1	-1	-1	-1	-1	-1	-1	1	1	-1	-1	1	CNA-high
163	0	1	1	0	-1	0	-1	-1	1	0	0	-1	0	0	-1	1	0	0	0	0	0	0	0	0	0	0	-1	-1	-1	-1	0	1	-1	-1	-1	1	1	-1	0	1	-1	CNA-high
164	0	1	0	0	0	0	0	0	0	0	0	-1	0	0	0	0	0	0	0	0	0	0	0	0	0	0	0	0	0	0	0	0	0	0	0	1	0	0	0	0	0	CNA-Low
165	0	1	0	1	0	-1	1	0	1	1	1	1	1	0	-1	1	0	0	1	0	0	0	0	0	0	0	0	0	0	-1	1	1	-1	-1	0	0	0	1	0	1	-1	CNA-Low
166	0	0	0	0	0	0	0	0	0	0	0	-1	1	1	0	0	-1	0	-1	-1	0	0	0	0	0	0	0	0	0	-1	0	0	0	0	0	0	0	0	0	0	0	CNA-Low
167	1	0	0	0	-1	0	1	1	0	0	1	-1	1	0	-1	1	0	1	1	-1	1	0	0	0	0	0	-1	1	1	0	1	1	1	0	-1	1	1	1	0	-1	1	CNA-high
168	0	1	1	1	0	0	0	0	1	1	1	-1	0	0	0	0	-1	0	1	0	0	0	0	0	0	0	0	0	0	0	1	0	0	-1	0	0	0	0	0	0	0	CNA-Low
169	0	1	1	0	-1	1	0	0	1	-1	0	0	0	0	0	0	-1	-1	1	0	-1	0	0	0	-1	-1	0	0	-1	-1	1	0	-1	-1	0	0	0	0	-1	0	0	CNA-Low
170	0	1	1	1	0	-1	-1	-1	1	-1	1	-1	1	1	-1	1	-1	0	1	1	-1	-1	1	1	-1	-1	0	1	-1	0	1	1	0	-1	1	1	0	-1	1	0	0	CNA-high
171	-1	1	1	1	-1	-1	-1	-1	0	-1	1	-1	1	0	-1	1	0	0	1	1	0	0	1	1	-1	0	-1	-1	-1	-1	1	-1	-1	0	-1	-1	1	0	1	-1	0	CNA-high
172	0	1	0	1	-1	0	1	0	1	1	1	-1	-1	-1	0	1	-1	-1	1	0	1	0	0	1	-1	0	-1	-1	-1	-1	1	1	1	-1	1	1	1	-1	-1	0	0	CNA-high
173	0	0	0	0	-1	1	-1	-1	1	-1	-1	-1	1	1	1	1	-1	-1	0	0	0	0	0	0	-1	1	0	0	0	0	0	0	0	0	0	1	1	-1	1	0	0	CNA-Low
174	0	1	0	0	0	0	0	0	1	0	0	-1	0	0	0	0	0	0	0	0	0	0	0	0	0	0	0	0	0	0	0	0	-1	0	0	-1	0	0	0	0	0	CNA-Low
175	0	0	-1	1	-1	1	-1	-1	1	-1	0	0	1	1	-1	0	-1	1	0	0	0	0	1	1	1	0	0	1	-1	-1	0	-1	-1	0	0	1	1	0	1	-1	1	CNA-high
176	0	1	0	0	-1	-1	0	0	1	-1	0	0	1	-1	0	0	0	0	0	0	-1	-1	-1	-1	0	0	-1	-1	-1	-1	1	0	-1	-1	-1	-1	0	1	0	0	0	CNA-high
177	0	1	0	0	0	0	-1	-1	0	0	1	-1	0	0	0	0	0	0	0	0	-1	-1	0	1	-1	1	-1	0	0	1	1	0	0	0	0	0	0	-1	0	0	0	CNA-Low
178	0	1	0	0	1	0	-1	0	1	0	1	-1	0	0	-1	1	0	0	0	0	-1	-1	0	0	-1	0	-1	-1	-1	-1	1	0	0	0	0	-1	0	0	-1	-1	0	CNA-Low
179	0	1	1	1	-1	1	0	0	0	-1	0	0	1	1	1	1	-1	-1	0	1	0	0	1	0	1	1	0	1	0	1	1	1	0	-1	1	-1	1	0	1	1	0	CNA-high
180	0	1	1	1	-1	1	-1	-1	1	-1	0	1	1	1	0	0	0	0	1	0	0	1	0	1	1	-1	0	1	1	-1	0	-1	-1	-1	0	0	1	-1	0	1	-1	CNA-high
181	-1	1	1	1	0	0	0	0	1	1	0	-1	0	0	0	1	0	-1	0	0	0	0	1	-1	-1	0	0	0	0	-1	1	0	0	0	0	0	0	-1	-1	0	0	CNA-Low
182	0	1	0	0	-1	0	0	0	-1	-1	0	-1	0	0	-1	1	1	-1	0	0	0	0	-1	-1	0	0	-1	-1	0	-1	0	0	0	-1	0	0	0	1	0	0	0	CNA-Low
183	0	1	0	-1	1	1	0	0	1	1	0	-1	1	0	-1	1	-1	-1	0	0	0	0	0	0	-1	0	0	1	0	-1	1	0	-1	0	-1	0	0	-1	-1	0	0	CNA-Low
184	-1	1	0	0	0	0	0	0	0	0	0	0	0	0	0	0	-1	-1	0	0	0	0	0	0	-1	-1	0	0	0	-1	0	0	0	-1	0	-1	1	-1	-1	0	0	CNA-Low
185	0	1	0	0	-1	-1	-1	-1	1	1	1	-1	0	1	1	1	-1	-1	1	1	-1	-1	-1	-1	0	1	0	-1	-1	0	0	0	0	-1	1	1	0	0	-1	0	0	CNA-high
186	0	1	1	1	-1	-1	0	-1	1	0	1	-1	1	1	1	1	-1	0	0	0	1	-1	0	0	-1	0	0	1	1	-1	1	1	1	-1	0	1	1	0	-1	-1	1	CNA-high
187	0	1	0	0	-1	-1	0	0	1	-1	1	0	1	1	0	0	0	0	0	0	0	0	-1	-1	-1	-1	-1	0	0	-1	0	-1	-1	0	0	0	0	0	0	0	0	CNA-Low
188	-1	1	0	0	-1	0	0	0	0	0	-1	-1	1	1	1	1	0	0	1	1	-1	0	0	1	-1	1	-1	1	1	-1	-1	0	-1	-1	-1	0	0	-1	0	0	0	CNA-high
189	0	0	0	0	0	0	0	0	0	0	0	-1	0	0	0	0	0	0	0	0	0	0	0	0	0	0	0	0	0	0	0	-1	-1	0	0	0	0	0	0	0	0	CNA-Low
190	-1	1	0	0	0	0	0	0	0	0	0	-1	0	-1	1	1	0	-1	1	1	0	0	1	0	0	0	0	0	-1	-1	-1	-1	-1	-1	-1	0	1	-1	-1	0	0	CNA-Low
191	1	1	-1	1	-1	-1	1	-1	1	-1	0	-1	1	1	-1	0	-1	-1	1	0	1	1	0	0	0	0	-1	1	0	-1	0	0	-1	0	0	1	1	0	-1	-1	1	CNA-high
192	0	1	1	1	0	0	0	0	1	0	1	-1	0	0	-1	1	0	0	0	0	0	0	-1	0	0	0	0	0	0	-1	0	0	0	0	0	0	0	0	-1	0	0	CNA-Low
193	0	0	0	0	0	0	0	0	0	0	0	0	0	0	0	0	0	0	0	0	0	0	-1	0	0	0	0	0	0	0	0	0	-1	0	0	0	0	-1	0	0	0	CNA-Low
194	0	0	0	0	0	1	0	0	1	0	1	-1	1	1	-1	1	0	-1	-1	-1	-1	1	0	0	-1	0	-1	0	0	-1	1	0	-1	-1	-1	-1	0	-1	0	-1	1	CNA-high
195	0	1	1	1	-1	1	-1	0	0	0	1	1	0	0	-1	0	1	1	0	0	1	1	1	0	0	0	0	1	0	0	1	0	-1	-1	0	1	1	0	0	1	0	CNA-high
196	-1	1	1	0	-1	0	-1	-1	1	0	0	-1	0	0	1	1	1	-1	-1	0	1	1	1	1	0	1	-1	0	0	1	1	1	1	-1	1	0	0	0	-1	1	-1	CNA-high
197	0	0	0	1	1	-1	0	0	0	0	1	-1	1	1	1	1	-1	-1	0	0	-1	1	-1	0	0	0	-1	-1	-1	-1	1	0	0	0	0	-1	0	-1	0	0	0	CNA-high
198	0	0	1	1	-1	1	0	0	0	0	0	-1	0	1	-1	0	0	0	0	-1	0	0	0	0	-1	0	-1	1	-1	-1	1	0	0	-1	0	1	1	0	-1	0	0	CNA-Low
199	0	1	0	0	-1	1	0	-1	1	-1	1	-1	1	1	-1	1	1	0	1	-1	0	1	-1	-1	0	1	-1	0	-1	-1	0	0	-1	0	-1	1	1	-1	0	0	1	CNA-high
200	1	1	1	1	0	1	1	1	0	-1	1	0	0	-1	-1	-1	0	-1	0	0	0	0	1	1	0	0	0	1	1	0	1	1	1	-1	1	1	1	-1	-1	1	0	CNA-high

(Continued)

201	0	1	0	0	-1	-1	0	0	0	0	1	-1	0	0	-1	1	0	0	0	0	0	0	-1	-1	0	0	0	0	0	0	0	0	0	-1	-1	0	0	0	0	0	0	CNA-Low
202	0	1	0	-1	-1	1	0	0	1	0	1	-1	1	1	-1	1	-1	1	0	0	-1	-1	-1	0	-1	1	-1	0	0	-1	1	-1	-1	-1	-1	-1	1	1	0	-1	0	CNA-high
203	1	1	0	0	-1	1	1	1	1	0	-1	-1	1	1	-1	0	1	1	0	0	-1	0	0	1	-1	0	0	1	-1	-1	1	0	0	-1	0	-1	-1	-1	-1	0	0	CNA-high
204	0	1	0	0	-1	-1	0	-1	1	1	1	1	0	0	-1	1	-1	-1	-1	-1	0	0	0	1	-1	-1	-1	0	0	-1	0	-1	-1	-1	-1	0	0	0	-1	-1	-1	CNA-high
205	0	0	1	0	0	0	1	1	1	-1	-1	-1	1	1	1	0	-1	-1	1	-1	-1	1	0	0	-1	0	0	1	0	-1	1	1	1	-1	-1	-1	1	0	0	-1	0	CNA-high
206	0	0	0	0	0	0	0	0	1	0	-1	-1	0	0	0	1	0	0	0	0	0	0	0	0	0	-1	-1	0	0	-1	0	0	0	0	0	-1	-1	0	-1	0	0	CNA-Low
207	0	1	1	0	0	0	-1	-1	0	-1	1	-1	1	1	-1	-1	0	0	1	-1	0	0	1	1	-1	-1	1	0	0	-1	1	1	0	1	1	0	0	1	0	0	0	CNA-high
208	0	1	0	0	-1	1	0	0	1	0	0	0	0	1	-1	0	1	1	-1	-1	0	0	0	0	0	0	0	1	0	-1	0	1	-1	0	0	0	1	0	0	0	0	CNA-Low
209	0	0	0	0	-1	-1	1	1	1	-1	1	-1	0	0	-1	1	0	-1	0	-1	0	0	-1	-1	-1	-1	0	0	0	-1	-1	-1	0	-1	-1	0	0	0	0	0	0	CNA-high
210	0	0	0	0	0	0	0	0	0	0	0	0	0	0	0	0	0	0	0	0	0	0	0	0	0	0	0	0	0	0	0	-1	-1	0	0	0	0	0	0	0	0	CNA-Low
211	0	1	0	0	-1	-1	-1	0	1	0	0	-1	1	1	-1	0	-1	-1	1	0	-1	1	-1	0	-1	0	-1	-1	-1	-1	0	1	0	-1	-1	0	1	0	1	0	0	CNA-high
212	1	1	0	0	-1	-1	0	0	1	0	0	0	0	0	-1	1	1	1	0	0	1	1	1	1	-1	0	0	-1	0	-1	1	1	1	-1	-1	1	1	0	0	0	0	CNA-high
213	0	0	0	0	-1	-1	0	0	0	-1	0	-1	1	0	-1	1	-1	-1	1	0	0	0	-1	-1	-1	-1	-1	0	0	-1	1	0	0	-1	-1	0	0	0	-1	-1	-1	CNA-high
214	0	1	0	0	0	0	0	0	0	0	0	0	1	1	0	0	0	0	0	1	0	0	1	1	0	0	1	1	1	0	0	0	0	0	0	1	1	0	0	0	0	CNA-Low
215	-1	1	0	0	-1	1	-1	-1	0	-1	0	-1	1	0	-1	1	0	0	0	1	0	0	0	0	-1	0	0	-1	-1	0	0	-1	0	-1	1	1	1	-1	0	1	-1	CNA-high
216	0	1	0	0	0	1	0	0	0	0	1	-1	1	0	0	1	0	0	0	0	0	0	0	0	0	0	0	0	0	-1	0	0	0	-1	-1	1	1	0	0	0	0	CNA-Low
217	0	1	0	0	0	0	0	0	0	0	0	0	0	0	0	0	0	0	0	0	0	0	-1	0	0	0	0	0	0	0	0	-1	-1	0	0	1	-1	0	0	0	0	CNA-Low
218	0	1	0	0	0	0	1	1	0	0	-1	-1	0	0	0	0	-1	-1	0	0	-1	0	1	1	0	0	0	0	1	-1	1	1	-1	-1	0	0	0	-1	0	0	0	CNA-Low
219	-1	-1	0	0	-1	-1	0	0	0	0	-1	-1	1	0	0	0	-1	-1	0	0	0	0	0	0	-1	0	0	0	0	0	0	-1	-1	0	0	-1	-1	-1	0	-1	0	CNA-Low
220	1	0	0	0	0	0	1	1	1	1	-1	-1	1	1	0	0	-1	1	-1	-1	0	0	0	0	-1	1	-1	1	0	-1	-1	-1	-1	0	0	0	0	0	1	0	0	CNA-high
221	0	1	0	0	0	0	0	-1	0	0	0	0	1	1	-1	1	-1	0	1	0	0	1	-1	0	0	-1	0	1	1	0	0	0	0	-1	1	-1	1	-1	-1	0	1	CNA-Low
222	0	1	0	0	-1	-1	1	-1	1	-1	0	-1	0	0	0	1	0	-1	-1	-1	0	0	1	0	-1	-1	-1	-1	0	0	1	0	-1	-1	-1	-1	0	0	-1	0	0	CNA-high
223	1	1	1	1	0	0	0	0	1	0	0	0	1	0	-1	1	0	0	0	0	1	1	1	0	0	1	0	1	0	0	0	1	0	0	0	1	1	0	1	1	1	CNA-Low
224	0	0	-1	0	0	0	0	0	0	0	1	-1	0	0	-1	1	-1	-1	0	0	0	0	0	0	0	-1	0	1	1	-1	0	-1	-1	0	0	1	0	0	0	0	0	CNA-Low
225	-1	1	1	0	0	-1	-1	-1	-1	-1	1	-1	0	0	1	1	-1	-1	0	0	1	0	0	0	-1	1	-1	0	0	0	0	0	0	-1	-1	0	0	-1	-1	-1	0	CNA-high
226	0	0	0	0	-1	0	-1	-1	1	-1	0	0	0	0	-1	1	0	-1	0	0	0	0	0	-1	-1	1	0	-1	0	0	0	0	0	0	0	0	0	0	1	0	0	CNA-Low
227	-1	-1	1	1	0	1	0	0	0	0	1	0	1	1	-1	1	0	0	0	1	1	0	0	0	1	0	-1	-1	-1	-1	1	0	0	0	0	1	1	-1	-1	0	0	CNA-high
228	1	1	0	0	-1	-1	0	0	1	1	1	1	1	1	1	1	-1	-1	0	-1	0	0	0	0	0	1	0	1	1	0	0	0	0	0	0	1	0	1	0	0	0	CNA-Low
229	0	1	0	1	0	0	0	0	1	1	1	-1	0	0	-1	0	-1	-1	0	-1	0	0	0	0	-1	0	0	0	0	0	1	-1	-1	0	0	-1	1	1	-1	0	0	CNA-Low
230	0	0	1	0	0	1	0	0	1	0	1	0	0	0	0	0	0	0	0	0	0	0	1	1	0	0	0	0	0	1	1	0	0	0	0	1	1	1	1	0	0	CNA-Low
231	-1	1	1	-1	-1	-1	0	0	1	0	0	-1	0	0	-1	1	-1	0	-1	0	0	-1	0	0	-1	-1	0	-1	-1	-1	0	1	0	0	0	1	1	-1	-1	-1	1	CNA-high
232	1	1	1	1	-1	1	-1	-1	1	0	0	0	1	1	-1	0	-1	-1	-1	-1	0	0	0	0	-1	0	0	1	1	-1	1	1	-1	-1	1	1	1	0	1	-1	1	CNA-high
233	0	0	0	0	0	0	0	0	0	0	0	0	0	0	0	0	0	0	0	0	0	0	0	0	0	-1	0	0	0	-1	0	0	0	0	0	0	0	0	-1	0	0	CNA-Low
234	0	0	0	0	-1	0	0	0	1	-1	0	0	1	1	0	1	0	0	0	0	0	0	-1	0	0	0	-1	-1	-1	-1	0	-1	0	-1	0	1	1	0	1	0	0	CNA-Low
235	1	1	-1	-1	-1	1	0	0	1	-1	1	0	1	0	-1	0	-1	0	-1	-1	1	0	-1	-1	1	1	-1	0	0	1	1	1	-1	-1	-1	0	0	0	-1	0	0	CNA-high
236	1	1	0	0	-1	-1	-1	-1	1	0	1	0	1	1	-1	0	-1	0	-1	-1	0	0	1	-1	0	0	-1	1	-1	-1	1	1	-1	-1	1	1	1	1	-1	-1	1	CNA-high
237	0	1	0	0	0	0	0	0	0	0	0	0	0	0	0	0	0	0	0	0	0	0	0	0	0	0	0	0	0	0	0	0	0	0	0	0	0	0	0	0	0	CNA-Low
238	1	1	0	0	0	1	0	0	0	0	1	1	1	1	-1	1	0	1	-1	-1	1	1	0	-1	-1	1	-1	1	-1	-1	-1	1	1	0	0	-1	1	-1	-1	0	0	CNA-high
239	0	1	0	0	-1	0	0	0	1	1	0	0	0	0	-1	0	-1	-1	0	0	-1	-1	0	-1	-1	0	0	0	0	-1	1	-1	-1	0	0	0	0	0	0	-1	1	CNA-Low
240	0	1	1	1	0	0	-1	-1	1	-1	0	-1	1	1	-1	1	1	0	1	1	0	0	-1	-1	-1	-1	-1	1	0	-1	1	0	0	-1	0	0	0	-1	0	0	0	CNA-high

(Continued)

241	0	0	0	0	0	0	0	-1	0	0	0	0	0	0	0	1	0	0	0	0	0	0	0	0	-1	0	-1	0	0	0	0	0	0	0	0	0	0	-1	0	0	0	CNA-Low
242	0	1	1	0	0	0	0	0	0	0	0	0	0	-1	-1	1	-1	0	0	1	0	1	1	1	-1	-1	0	0	0	1	1	0	0	0	0	0	0	-1	-1	0	0	CNA-Low
243	0	1	1	1	-1	0	-1	-1	1	0	1	-1	1	1	0	1	-1	-1	0	0	-1	1	1	1	-1	-1	0	1	1	-1	0	0	0	-1	0	-1	-1	-1	0	0	0	CNA-high
244	1	0	0	0	-1	-1	0	0	1	0	-1	-1	1	1	-1	0	-1	-1	-1	-1	0	0	0	0	-1	0	-1	0	0	-1	0	-1	-1	-1	-1	0	0	0	0	0	0	CNA-Low
245	0	1	1	1	1	-1	0	0	0	0	1	-1	1	0	-1	1	0	0	1	1	0	0	0	0	-1	0	0	0	0	0	0	-1	-1	0	0	0	0	0	0	0	0	CNA-Low
246	0	0	0	0	0	0	0	0	0	0	0	0	0	0	0	0	0	0	0	0	0	0	0	0	0	0	0	0	0	0	0	0	0	0	0	0	0	0	0	0	0	CNA-Low
247	0	1	0	0	0	0	0	-1	1	0	0	0	1	1	-1	1	1	-1	1	0	0	0	0	0	-1	0	-1	0	0	0	0	0	-1	0	0	0	0	0	0	0	0	CNA-Low
248	0	1	0	0	-1	-1	1	1	1	1	-1	-1	0	0	0	0	1	1	0	0	0	0	-1	-1	1	0	0	0	0	0	0	1	1	0	1	1	0	1	-1	0	0	CNA-Low
249	1	1	0	0	-1	0	0	0	0	0	-1	-1	0	0	0	0	0	0	0	0	0	0	0	0	0	0	0	0	0	0	0	0	-1	0	0	0	0	0	0	0	0	CNA-Low
250	0	1	1	1	0	0	0	-1	1	-1	1	0	1	0	-1	1	-1	-1	0	-1	0	0	0	1	-1	0	1	-1	-1	-1	0	0	0	-1	-1	0	1	-1	0	0	-1	CNA-high
251	0	1	0	0	-1	1	1	1	0	0	0	0	1	-1	0	0	0	0	-1	-1	1	1	1	0	0	1	0	1	-1	-1	0	-1	-1	0	0	-1	0	0	0	0	0	CNA-Low
252	1	1	0	0	0	0	1	-1	1	-1	1	-1	1	0	-1	1	-1	-1	0	1	0	1	-1	0	-1	-1	-1	1	1	-1	1	-1	-1	-1	-1	0	1	1	-1	0	0	CNA-high
253	1	1	0	0	-1	-1	0	0	1	1	0	-1	1	1	-1	1	-1	-1	-1	-1	1	0	1	-1	-1	1	0	1	1	-1	-1	-1	-1	-1	0	-1	1	0	1	0	0	CNA-high
254	0	1	0	0	0	0	0	0	0	0	1	-1	0	0	0	0	0	0	0	0	0	0	0	0	0	0	0	0	0	0	0	0	0	-1	0	0	0	0	0	-1	1	CNA-Low
255	0	1	0	1	0	1	0	0	0	-1	0	0	0	-1	-1	1	0	1	1	-1	0	0	1	1	-1	1	-1	0	0	-1	1	0	1	0	0	1	1	0	0	0	0	CNA-Low
256	0	1	0	0	0	0	0	0	0	0	1	0	1	1	0	1	0	0	0	0	0	0	0	0	0	1	0	0	-1	0	0	0	0	-1	-1	0	0	0	0	0	0	CNA-Low
257	-1	1	-1	-1	0	-1	0	0	1	-1	0	0	0	0	1	1	0	0	0	0	0	0	0	0	0	0	-1	0	0	0	0	0	0	-1	1	0	0	-1	0	-1	1	CNA-Low
258	0	1	-1	0	-1	-1	0	0	1	1	1	-1	-1	-1	0	0	-1	-1	0	0	-1	-1	0	0	-1	-1	1	0	0	0	0	-1	-1	-1	0	1	-1	-1	1	0	0	CNA-high
259	0	0	0	0	0	0	0	0	-1	-1	0	0	1	1	0	0	0	0	0	-1	0	0	0	0	0	0	0	0	0	-1	1	1	1	1	0	1	1	-1	-1	0	0	CNA-Low
260	0	0	0	0	-1	-1	0	0	0	0	1	0	1	0	0	0	-1	-1	0	0	1	1	0	0	-1	-1	-1	1	1	0	1	0	-1	0	0	0	1	1	0	0	0	CNA-Low
261	0	1	1	1	0	0	1	0	1	0	1	0	1	1	0	1	0	0	1	1	0	0	0	0	0	1	0	1	1	1	1	1	0	0	1	1	1	1	0	0	1	CNA-high
262	0	1	0	0	0	0	0	0	1	1	0	0	0	0	1	1	0	0	0	0	0	0	-1	-1	0	-1	0	1	-1	0	0	-1	-1	0	0	0	0	1	-1	0	0	CNA-Low
263	0	0	0	0	0	0	0	0	0	0	0	0	0	0	0	0	-1	-1	0	0	0	0	0	0	0	0	0	0	0	0	0	-1	-1	0	0	0	0	0	0	0	0	CNA-Low
264	0	1	0	0	0	0	0	0	0	0	0	0	0	0	0	0	-1	-1	0	0	0	0	0	0	-1	0	-1	1	1	0	0	0	0	0	0	-1	0	0	0	0	0	CNA-Low
265	0	0	0	1	0	0	1	0	1	0	-1	-1	1	1	-1	1	0	0	1	0	-1	-1	1	1	-1	1	0	0	0	-1	1	0	0	-1	1	1	1	-1	0	0	0	CNA-high
266	0	0	0	0	-1	0	-1	-1	1	-1	1	1	1	1	-1	-1	0	0	-1	-1	0	0	0	0	0	1	-1	0	-1	0	1	-1	-1	-1	0	1	1	1	0	1	-1	CNA-high
267	0	0	0	0	0	0	0	0	1	0	0	-1	0	0	-1	1	-1	0	0	0	0	0	0	0	-1	0	0	0	0	0	0	0	0	-1	-1	0	0	0	0	0	0	CNA-Low
268	0	0	-1	-1	-1	-1	0	0	0	0	0	0	1	1	0	0	-1	-1	0	0	-1	-1	0	0	-1	0	0	-1	-1	-1	0	-1	-1	-1	-1	-1	0	0	1	-1	-1	CNA-high
269	-1	1	0	0	-1	-1	-1	-1	-1	-1	1	-1	1	1	1	1	-1	-1	1	1	0	0	1	1	0	-1	1	1	1	1	1	1	1	-1	0	0	0	1	1	0	0	CNA-high
270	1	1	0	0	-1	-1	0	0	1	-1	1	0	1	1	0	0	-1	0	0	0	0	1	0	0	-1	0	0	0	0	0	0	1	1	-1	1	1	1	1	0	0	1	CNA-Low
271	0	-1	1	1	0	0	1	1	1	-1	1	-1	0	-1	-1	1	-1	-1	-1	-1	1	1	1	1	1	0	1	-1	1	0	1	1	1	-1	-1	1	1	-1	1	0	0	CNA-high
272	0	0	0	0	0	0	-1	-1	0	0	-1	-1	0	0	-1	0	-1	0	0	0	0	-1	0	0	0	0	0	0	0	-1	0	0	-1	-1	1	-1	-1	-1	-1	0	0	CNA-Low
273	0	0	0	0	-1	1	0	0	1	0	1	-1	1	0	0	1	-1	-1	1	0	0	1	-1	1	-1	0	0	0	0	-1	1	0	0	0	1	0	-1	1	-1	-1	1	CNA-high
274	0	1	1	1	0	0	0	0	1	-1	0	-1	1	1	-1	1	0	0	-1	-1	0	0	0	0	-1	-1	-1	1	-1	0	0	1	0	1	0	0	0	-1	-1	-1	1	CNA-high
275	0	1	0	0	-1	1	1	1	1	1	-1	-1	1	1	-1	1	-1	-1	-1	1	-1	0	-1	1	-1	0	-1	0	-1	-1	1	-1	-1	1	-1	1	1	-1	1	-1	0	CNA-high
276	0	0	0	0	0	0	-1	-1	-1	-1	0	0	1	1	0	0	-1	-1	0	-1	0	0	1	1	0	-1	1	-1	-1	0	0	0	0	0	0	0	0	0	-1	0	0	CNA-Low
277	0	1	0	0	-1	0	0	0	1	-1	-1	-1	0	0	-1	-1	-1	0	0	0	0	1	0	0	0	1	0	1	-1	-1	0	0	0	0	0	-1	1	0	1	0	0	CNA-Low
278	0	1	1	1	-1	0	-1	-1	1	-1	1	1	-1	1	0	1	-1	-1	-1	0	-1	1	-1	1	0	1	-1	-1	-1	1	1	-1	-1	-1	-1	1	1	-1	-1	0	0	CNA-high
279	0	1	0	0	-1	0	0	-1	1	1	-1	-1	0	0	0	0	0	-1	0	-1	0	0	1	0	0	-1	-1	0	0	0	0	1	1	-1	0	1	1	-1	-1	0	0	CNA-Low
280	0	1	1	1	0	0	0	0	1	-1	0	0	1	1	-1	0	-1	-1	0	0	-1	1	-1	-1	-1	1	-1	0	0	-1	1	0	0	0	0	0	0	-1	-1	0	0	CNA-high

(Continued)

281	0	1	1	0	0	0	0	0	1	-1	0	-1	1	1	0	1	0	0	1	0	-1	1	-1	-1	0	0	-1	0	0	-1	1	-1	-1	0	-1	0	0	0	-1	0	0	CNA-Low
282	0	0	0	0	0	0	0	0	0	0	1	-1	0	0	0	0	0	-1	0	0	0	0	0	0	0	0	0	1	1	0	0	0	0	-1	0	1	0	0	-1	0	0	CNA-Low
283	0	0	0	0	0	0	0	0	0	0	0	0	0	0	0	0	0	0	0	0	0	0	0	0	0	0	0	0	0	0	0	0	0	0	0	0	0	0	0	0	0	CNA-Low
284	1	1	0	0	-1	-1	-1	-1	1	-1	1	1	1	1	0	1	1	-1	-1	-1	1	1	-1	-1	-1	0	0	0	0	-1	1	0	0	0	0	1	1	0	1	0	0	CNA-high
285	0	0	0	0	-1	1	-1	1	1	-1	1	0	0	0	-1	1	-1	-1	-1	-1	0	0	-1	-1	-1	1	-1	0	0	-1	-1	0	0	-1	1	1	0	0	1	0	0	CNA-high
286	-1	1	1	1	-1	-1	-1	1	1	-1	1	-1	1	0	0	1	-1	-1	1	1	1	-1	1	1	-1	0	-1	-1	1	0	0	1	0	-1	1	1	0	0	-1	1	0	CNA-high
287	0	1	1	0	-1	-1	-1	-1	1	-1	1	0	1	1	-1	1	0	0	0	0	0	1	-1	-1	-1	0	-1	-1	-1	0	1	-1	-1	-1	0	-1	0	0	-1	0	0	CNA-high
288	0	0	0	0	0	0	0	0	0	0	0	0	0	0	0	0	0	0	0	0	0	0	-1	-1	0	0	0	0	0	0	0	0	0	0	0	0	0	0	0	0	0	CNA-Low
289	0	0	-1	1	-1	1	-1	0	1	-1	1	1	0	1	1	1	1	1	1	0	1	0	0	0	0	0	-1	1	-1	-1	1	-1	0	1	1	0	1	0	-1	0	0	CNA-high
290	0	0	0	0	0	0	0	0	0	0	0	0	0	0	0	0	0	0	0	0	0	0	0	0	0	0	0	0	0	0	0	0	0	0	0	0	0	0	0	0	0	CNA-Low
291	0	1	1	1	-1	0	-1	-1	1	-1	0	0	0	0	-1	-1	-1	-1	-1	-1	-1	-1	0	0	-1	-1	-1	1	1	-1	1	0	0	0	0	1	1	-1	-1	0	1	CNA-high
292	0	0	0	0	0	0	0	0	0	0	0	0	0	0	0	0	0	0	0	0	0	0	0	0	0	0	0	0	0	0	0	0	0	0	0	0	0	0	0	0	0	CNA-Low
293	0	1	1	1	0	0	0	0	1	0	1	-1	1	1	0	0	0	0	-1	-1	1	1	1	1	0	0	0	0	0	-1	0	0	0	0	0	0	0	0	0	-1	1	CNA-Low
294	0	0	0	0	-1	-1	0	0	0	0	0	0	0	0	-1	1	-1	-1	0	-1	0	0	0	0	-1	0	0	0	0	0	1	-1	-1	0	0	-1	1	-1	-1	-1	0	CNA-Low
295	0	1	1	0	0	0	0	0	1	0	1	0	1	0	-1	1	0	0	1	1	0	1	0	0	0	0	1	0	0	1	1	1	0	0	0	-1	0	1	1	0	0	CNA-Low
296	0	1	1	0	0	0	0	0	1	-1	0	1	0	0	-1	1	-1	-1	0	0	0	0	0	1	0	1	0	1	1	1	1	1	-1	0	1	1	1	-1	-1	0	0	CNA-high
297	1	0	0	0	0	0	0	0	0	0	0	0	1	-1	-1	0	-1	-1	0	0	0	0	0	0	-1	0	0	1	-1	-1	1	0	0	0	0	0	0	0	0	0	0	CNA-Low
298	-1	1	1	1	-1	1	1	-1	0	0	0	0	0	0	-1	1	1	-1	-1	-1	-1	-1	1	1	-1	-1	0	0	0	0	1	0	0	-1	-1	1	1	-1	-1	0	0	CNA-high
299	0	0	0	0	0	0	0	0	0	0	0	0	0	0	0	0	0	0	0	0	0	0	0	0	-1	0	-1	0	0	0	0	0	0	0	0	0	0	0	0	0	0	CNA-Low
300	0	0	0	0	0	0	0	-1	1	-1	1	-1	-1	1	0	1	-1	-1	1	1	0	0	-1	0	1	-1	0	0	-1	0	0	-1	-1	-1	-1	0	0	0	-1	0	0	CNA-high
301	0	1	0	1	0	1	0	0	1	0	0	0	1	1	0	1	0	0	1	0	-1	0	0	0	0	0	0	0	0	0	0	0	0	0	1	-1	0	0	0	0	1	CNA-Low
302	0	1	0	0	0	0	0	0	0	0	1	-1	0	0	1	1	0	0	0	0	0	0	0	0	0	0	0	0	0	0	0	0	0	-1	0	0	0	0	0	0	0	CNA-Low
303	1	1	0	0	0	0	0	0	0	0	0	0	1	1	0	0	0	0	0	0	0	0	0	0	0	1	0	1	0	0	0	0	0	0	0	0	0	1	0	-1	1	CNA-Low
304	0	0	0	0	0	-1	0	0	0	0	0	0	0	0	0	0	0	0	0	0	0	0	0	0	0	0	0	0	0	0	0	0	0	0	0	0	0	0	0	0	0	CNA-Low
305	0	0	0	0	-1	0	0	0	1	-1	1	0	-1	0	-1	1	0	0	0	-1	0	0	1	0	-1	1	-1	0	-1	0	0	1	1	0	-1	1	1	1	-1	0	1	CNA-high
306	0	1	0	0	1	1	0	0	1	1	1	-1	1	1	1	1	-1	0	0	0	0	0	1	1	0	-1	0	0	0	0	0	-1	-1	-1	-1	0	0	0	0	0	0	CNA-Low
307	0	0	0	0	0	0	-1	0	1	0	-1	-1	1	0	-1	0	-1	-1	-1	-1	0	0	0	0	-1	0	0	0	0	-1	1	0	-1	0	-1	0	0	0	-1	-1	0	CNA-Low
308	1	1	0	0	0	0	1	1	1	1	0	0	1	1	1	1	-1	-1	0	0	0	0	1	0	-1	0	1	1	1	1	1	1	0	0	0	1	1	1	1	0	0	CNA-high
309	0	0	0	0	0	0	0	0	0	0	0	0	0	0	0	0	-1	-1	0	0	0	0	0	0	0	0	0	0	0	0	0	0	0	0	0	0	0	0	1	0	0	CNA-Low
310	0	0	0	0	-1	0	-1	-1	0	0	0	0	1	0	-1	0	0	-1	0	0	1	1	0	1	-1	1	0	0	0	0	0	0	0	0	0	1	1	-1	0	0	0	CNA-Low
311	0	1	1	1	0	0	0	0	1	1	0	0	0	0	0	0	0	0	0	0	1	1	1	1	0	1	0	1	1	0	0	1	1	1	1	-1	1	1	1	1	1	CNA-high
312	0	1	0	0	1	1	0	0	0	0	0	0	-1	-1	1	1	0	0	0	0	1	1	-1	1	-1	1	0	1	-1	-1	1	0	0	0	1	1	0	-1	0	0	0	CNA-Low
313	0	0	1	1	0	0	0	0	0	0	0	0	1	0	0	0	0	0	0	0	0	0	0	0	0	0	0	0	0	0	0	0	0	0	0	-1	-1	0	0	0	0	CNA-Low
314	1	1	0	0	0	0	0	0	1	0	1	-1	0	0	0	0	-1	0	-1	-1	0	0	-1	-1	0	0	-1	0	0	-1	0	0	0	-1	-1	0	0	0	0	0	0	CNA-Low
315	-1	1	0	0	0	0	0	-1	1	-1	1	-1	0	0	0	1	0	0	0	0	0	0	0	0	0	0	0	0	0	-1	0	0	0	0	0	0	0	0	0	0	0	CNA-Low
316	0	0	0	0	0	1	1	0	1	0	1	0	1	1	-1	1	-1	0	0	0	-1	0	1	1	-1	1	-1	1	-1	0	1	1	-1	-1	0	0	0	-1	-1	0	0	CNA-high
317	0	0	0	0	-1	1	-1	-1	1	-1	0	0	1	-1	-1	1	0	-1	1	1	0	0	-1	0	-1	-1	-1	-1	-1	-1	1	-1	-1	-1	-1	0	1	0	1	1	-1	CNA-high
318	-1	1	0	0	-1	-1	1	-1	-1	-1	0	0	1	1	0	0	-1	-1	0	0	0	0	0	0	1	-1	0	0	0	0	0	-1	-1	0	0	0	0	0	0	-1	0	CNA-Low
319	0	0	1	1	0	0	1	1	1	1	0	0	1	1	-1	1	-1	-1	0	0	0	0	1	1	-1	0	-1	1	-1	0	0	-1	-1	-1	0	0	1	0	-1	0	0	CNA-high
320	0	1	0	0	-1	0	-1	-1	1	0	-1	0	0	0	0	0	1	0	-1	-1	1	0	1	1	0	1	-1	0	0	0	0	1	1	-1	0	1	1	-1	-1	0	0	CNA-high

(Continued)

321	1	1	1	0	-1	-1	-1	0	1	-1	0	0	1	-1	-1	1	-1	-1	1	-1	-1	1	-1	-1	-1	1	-1	1	1	1	1	1	-1	-1	-1	1	1	0	-1	-1	0	CNA-high
322	1	0	1	0	-1	1	1	-1	1	0	0	0	0	0	-1	1	-1	-1	0	0	0	0	1	0	-1	-1	0	0	-1	-1	0	-1	-1	-1	0	-1	0	0	0	0	-1	CNA-high
323	0	1	0	0	0	-1	0	0	0	0	1	0	1	0	1	1	0	0	0	0	0	0	0	0	0	0	0	0	0	0	0	0	0	0	0	1	1	0	0	0	0	CNA-Low
324	0	1	1	1	-1	-1	0	0	0	0	-1	-1	1	1	-1	1	1	-1	0	0	0	0	-1	-1	-1	0	-1	0	0	-1	1	0	-1	-1	-1	0	0	1	0	0	0	CNA-high
325	-1	1	0	0	0	0	0	0	1	1	1	-1	0	0	0	0	0	0	1	1	-1	-1	-1	-1	-1	-1	-1	1	1	-1	0	-1	-1	-1	1	0	-1	-1	-1	0	0	CNA-high
326	-1	0	0	0	0	0	1	-1	1	1	0	-1	0	0	-1	0	0	0	0	0	0	0	0	-1	0	-1	0	-1	-1	-1	0	0	-1	-1	0	-1	-1	0	-1	-1	0	CNA-Low
327	0	1	0	0	0	0	0	0	0	0	1	-1	0	0	0	0	0	-1	0	0	0	0	-1	0	-1	0	0	-1	0	0	0	0	0	0	0	-1	0	0	0	0	0	CNA-Low
328	0	0	0	0	-1	-1	0	-1	1	1	0	0	1	1	0	0	-1	-1	-1	-1	0	0	-1	-1	-1	0	-1	0	0	1	1	0	0	0	0	-1	0	0	-1	0	0	CNA-Low
329	0	1	0	0	-1	-1	0	0	1	-1	1	-1	0	0	1	1	1	-1	1	1	1	0	1	1	0	0	0	0	-1	0	1	-1	-1	-1	-1	0	0	-1	0	0	0	CNA-high
330	0	0	-1	1	-1	0	1	1	1	-1	1	1	-1	0	1	1	-1	0	0	0	-1	0	1	1	-1	-1	0	-1	0	1	1	0	-1	-1	-1	0	0	-1	0	0	0	CNA-high
331	-1	0	0	0	0	0	-1	-1	0	0	-1	-1	1	1	-1	1	-1	0	0	0	-1	-1	0	0	-1	0	-1	-1	-1	-1	0	-1	-1	0	0	-1	-1	0	-1	-1	-1	CNA-high
332	1	1	0	0	0	0	1	0	0	0	0	-1	1	0	-1	1	-1	-1	1	1	0	0	0	0	-1	0	-1	1	1	0	0	0	0	-1	-1	0	0	0	0	0	0	CNA-Low
333	0	1	0	0	-1	0	0	0	1	0	0	0	-1	0	-1	1	0	0	1	1	-1	1	1	1	-1	0	-1	1	0	1	1	1	1	-1	-1	0	0	0	-1	0	0	CNA-high
334	0	0	0	0	-1	0	0	-1	1	0	0	0	1	1	0	0	0	0	0	0	0	1	0	0	0	0	-1	0	0	0	0	0	-1	-1	-1	1	1	0	-1	0	0	CNA-Low
335	0	0	0	0	0	0	0	0	1	1	0	-1	0	0	-1	0	0	0	0	0	0	0	0	0	0	0	0	0	0	0	0	-1	-1	0	0	0	0	0	0	0	0	CNA-Low
336	0	1	0	0	1	1	0	-1	1	-1	1	0	1	0	0	1	-1	-1	-1	-1	-1	0	-1	-1	-1	-1	-1	1	1	-1	1	-1	-1	-1	-1	0	0	1	0	0	0	CNA-high
337	0	0	0	0	0	0	0	0	1	1	1	1	1	0	-1	0	0	0	0	0	0	0	0	0	0	0	0	0	0	-1	0	-1	-1	0	0	0	0	0	0	0	0	CNA-Low
338	1	1	0	0	-1	-1	0	0	0	0	0	0	0	0	0	0	-1	-1	-1	-1	0	0	0	0	-1	-1	-1	1	-1	0	0	0	0	0	0	0	0	0	-1	0	0	CNA-Low
339	0	0	0	0	0	0	0	0	0	0	0	0	0	0	0	0	0	0	0	0	0	0	0	0	0	0	0	0	0	0	0	0	0	0	0	0	0	0	0	0	0	CNA-Low
340	0	1	1	0	1	1	0	0	0	0	0	-1	1	0	0	1	0	0	1	-1	1	1	1	1	-1	1	-1	1	-1	-1	1	1	0	-1	1	0	0	0	0	0	0	CNA-high
341	0	1	1	-1	1	1	0	0	1	-1	1	-1	1	1	-1	1	1	0	1	0	0	0	0	0	-1	0	-1	-1	-1	0	1	1	0	0	0	-1	1	1	-1	0	1	CNA-high
342	0	1	0	1	0	0	0	0	1	-1	0	0	1	1	0	0	-1	-1	0	0	-1	-1	0	0	0	-1	0	0	0	0	0	0	0	-1	1	1	0	-1	-1	0	0	CNA-Low
343	1	1	0	0	0	0	1	0	0	0	1	-1	1	1	-1	1	0	-1	-1	-1	0	0	-1	-1	-1	1	-1	1	0	0	0	-1	-1	-1	-1	1	1	0	0	0	0	CNA-high
344	0	1	1	1	-1	1	-1	-1	0	-1	1	-1	0	1	-1	1	0	0	0	0	-1	-1	0	0	-1	1	-1	-1	-1	0	0	0	0	-1	1	1	-1	0	-1	0	0	CNA-high
345	0	1	0	0	-1	-1	-1	-1	0	1	0	0	1	0	-1	1	1	1	0	0	0	0	-1	-1	-1	0	-1	1	-1	-1	1	-1	-1	0	0	1	1	1	-1	-1	1	CNA-high
346	0	1	1	1	0	0	0	0	1	0	1	-1	1	1	1	1	0	-1	0	0	1	1	1	1	0	0	0	0	0	0	1	1	1	-1	0	1	0	0	0	1	1	CNA-high
347	-1	1	0	0	-1	0	-1	-1	0	0	0	0	0	0	-1	-1	0	0	1	-1	-1	-1	0	0	-1	0	-1	0	0	-1	-1	0	0	0	0	0	0	-1	-1	0	0	CNA-Low
348	1	1	0	0	0	0	0	0	0	0	0	0	1	1	-1	1	0	0	0	0	1	0	0	0	0	0	0	0	0	0	1	0	0	0	0	0	0	0	0	0	1	CNA-Low
349	0	0	0	0	0	0	0	0	0	0	0	0	0	0	0	0	0	0	0	0	0	0	0	0	0	0	0	0	0	0	0	0	0	0	0	0	0	0	0	0	0	CNA-Low
350	1	1	1	1	0	1	0	0	0	0	1	0	1	1	0	0	0	0	0	0	0	0	0	0	0	1	-1	1	1	0	0	1	1	1	1	1	1	1	-1	1	1	CNA-high
351	0	1	0	0	0	0	-1	-1	1	-1	0	0	1	1	1	1	0	0	0	0	0	1	0	0	-1	-1	-1	-1	-1	-1	1	0	0	0	0	1	1	0	0	-1	1	CNA-high
352	0	1	0	0	0	0	1	1	1	1	1	0	1	0	0	1	0	0	0	0	1	0	0	0	0	1	0	1	0	1	1	-1	0	0	0	1	1	1	0	0	0	CNA-Low
353	0	0	0	0	0	1	0	0	1	0	0	0	1	0	-1	1	-1	0	-1	-1	0	0	0	0	0	1	-1	0	-1	-1	1	0	-1	-1	0	0	0	0	0	0	0	CNA-Low
354	0	1	0	0	-1	1	0	0	0	0	0	0	0	0	0	1	0	0	0	0	0	0	0	0	-1	1	-1	0	0	0	0	-1	-1	-1	-1	0	0	1	0	0	0	CNA-Low
355	0	1	0	0	-1	0	-1	-1	1	1	-1	-1	0	0	-1	0	0	0	0	0	0	0	0	0	-1	-1	-1	0	0	-1	0	-1	-1	0	0	-1	-1	1	-1	-1	-1	CNA-high
356	1	1	0	0	-1	-1	-1	-1	0	1	1	1	1	-1	0	0	-1	1	-1	-1	-1	0	-1	-1	-1	0	-1	1	1	0	0	0	0	-1	-1	1	1	1	0	0	0	CNA-high
357	0	0	0	0	0	0	0	0	0	0	1	-1	0	0	0	1	0	0	0	0	0	0	0	0	-1	0	0	0	0	0	0	0	0	0	0	0	0	0	0	0	0	CNA-Low
358	0	1	0	1	0	0	0	0	0	0	0	-1	0	0	-1	0	0	0	0	0	0	0	0	0	-1	0	0	0	-1	0	0	0	0	0	0	0	0	0	-1	0	0	CNA-Low
359	-1	1	0	0	-1	-1	0	0	0	0	0	-1	-1	-1	0	0	0	0	0	0	0	0	0	0	0	0	0	0	0	0	0	0	0	0	0	0	0	0	0	0	0	CNA-Low
360	0	1	0	0	-1	-1	0	0	0	0	0	0	0	-1	0	0	-1	-1	0	0	0	0	-1	0	0	0	0	0	0	-1	1	0	-1	-1	-1	0	0	0	0	-1	1	CNA-Low

(Continued)

361	0	1	0	0	-1	0	0	0	1	1	1	-1	1	0	-1	-1	-1	-1	-1	-1	0	0	1	1	0	0	0	1	1	0	0	0	0	0	0	0	0	0	0	-1	0	CNA-Low
362	0	0	0	0	-1	0	-1	-1	1	-1	0	-1	1	1	-1	0	0	0	0	0	-1	0	0	0	-1	0	-1	-1	-1	-1	0	-1	0	-1	-1	-1	0	0	-1	-1	0	CNA-high
363	1	1	1	1	0	0	0	0	1	-1	0	0	1	1	0	0	-1	-1	1	0	-1	0	1	0	0	-1	-1	-1	-1	-1	1	1	-1	0	-1	-1	1	0	0	0	1	CNA-high
364	0	1	0	0	-1	0	-1	-1	1	-1	1	-1	1	1	-1	1	-1	-1	1	1	-1	1	1	0	0	0	-1	1	1	-1	1	1	-1	-1	1	0	1	-1	0	0	1	CNA-high
365	0	1	1	1	0	0	1	0	1	0	1	-1	1	0	-1	1	-1	1	1	-1	0	-1	-1	-1	0	0	0	0	0	-1	1	1	-1	0	0	1	0	1	-1	0	1	CNA-high
366	0	1	0	0	0	1	0	0	0	0	1	-1	0	1	-1	1	-1	-1	0	0	0	0	0	1	-1	0	-1	0	0	-1	1	0	0	-1	-1	-1	1	-1	-1	0	0	CNA-Low
367	1	1	0	0	-1	0	1	0	1	0	0	-1	0	0	-1	1	-1	-1	0	0	0	0	0	0	1	-1	-1	1	0	-1	0	-1	-1	0	0	-1	-1	0	1	0	0	CNA-Low
368	0	0	0	0	0	0	1	1	1	0	-1	-1	1	1	1	1	-1	-1	-1	-1	0	0	1	1	0	1	0	0	0	-1	1	0	0	-1	-1	1	1	-1	-1	0	0	CNA-high
369	-1	1	0	0	0	0	0	0	1	0	0	0	0	0	-1	0	-1	-1	1	1	0	0	1	1	1	-1	1	0	0	0	0	-1	-1	-1	1	1	1	1	1	0	0	CNA-high
370	0	1	0	0	-1	-1	0	0	1	-1	0	0	1	1	0	1	-1	0	1	1	-1	-1	0	0	0	-1	-1	-1	-1	-1	1	0	0	-1	-1	-1	0	0	-1	0	0	CNA-high
371	0	0	1	1	0	0	0	0	0	0	0	0	1	1	-1	0	-1	-1	-1	-1	-1	0	0	0	-1	1	0	0	0	1	1	0	0	0	0	0	0	0	0	0	0	CNA-Low
372	0	0	0	0	0	0	0	-1	1	0	0	0	1	1	-1	1	-1	-1	0	0	1	1	0	0	0	0	0	0	0	0	0	0	0	-1	-1	0	0	1	-1	0	0	CNA-Low
373	0	1	1	1	-1	-1	0	0	1	-1	0	-1	1	1	-1	1	1	1	0	0	0	1	-1	-1	-1	-1	-1	0	-1	0	0	0	0	-1	1	-1	-1	-1	0	0	0	CNA-high
374	0	0	0	0	-1	-1	0	0	0	0	0	-1	0	0	0	1	0	0	0	0	0	0	1	1	-1	0	-1	-1	-1	0	0	-1	-1	-1	-1	-1	-1	0	0	-1	1	CNA-Low
375	0	1	1	0	0	0	0	0	1	-1	0	-1	1	1	-1	1	-1	0	0	-1	0	0	0	-1	-1	0	-1	0	1	0	1	0	-1	-1	-1	-1	1	0	0	-1	0	CNA-high
376	1	1	0	0	1	1	-1	0	1	0	-1	-1	1	1	-1	1	1	-1	-1	-1	-1	1	1	1	-1	-1	-1	0	-1	-1	0	-1	-1	-1	-1	-1	0	1	-1	0	0	CNA-high
377	0	1	1	0	0	-1	0	0	0	0	0	0	0	0	-1	0	-1	0	0	0	0	0	0	0	0	0	0	1	0	-1	0	0	0	-1	0	0	0	0	0	0	0	CNA-Low
378	-1	1	0	0	0	0	0	-1	0	0	0	-1	1	0	-1	1	1	1	1	0	0	0	0	0	-1	-1	-1	-1	-1	-1	1	-1	-1	-1	-1	0	0	0	0	0	0	CNA-high
379	-1	1	0	0	-1	-1	1	-1	0	0	0	-1	-1	-1	1	1	-1	-1	1	1	0	0	1	1	-1	-1	-1	0	0	-1	-1	0	0	0	1	0	0	-1	-1	0	0	CNA-high
380	0	0	0	0	0	0	-1	1	1	-1	0	0	0	1	-1	0	0	0	1	0	-1	0	0	0	-1	0	-1	0	0	-1	1	0	-1	-1	0	0	0	0	0	-1	1	CNA-Low
381	1	1	0	0	-1	-1	1	1	1	-1	1	-1	1	1	0	0	-1	-1	-1	-1	1	1	1	0	-1	1	0	1	1	-1	1	-1	-1	-1	-1	1	1	1	-1	0	-1	CNA-high
382	-1	1	0	0	-1	-1	-1	-1	1	1	1	0	0	0	0	1	-1	-1	1	1	0	0	1	1	-1	0	-1	0	0	-1	1	1	1	0	0	0	0	0	0	0	0	CNA-high
383	-1	1	0	0	0	0	0	0	0	-1	1	-1	0	0	1	1	0	-1	1	0	-1	-1	1	1	0	1	-1	0	0	-1	1	0	0	0	0	1	1	-1	-1	0	0	CNA-high
384	0	0	0	0	-1	1	1	-1	1	1	1	-1	0	0	-1	1	-1	-1	0	-1	-1	-1	0	0	-1	1	-1	-1	0	-1	0	-1	-1	-1	0	1	1	-1	-1	0	0	CNA-high
385	0	1	0	0	0	0	0	0	1	1	0	0	0	0	1	1	0	0	0	0	0	0	0	0	0	0	0	0	0	0	0	0	0	-1	1	0	0	0	0	0	0	CNA-Low
386	-1	1	0	0	-1	-1	-1	-1	0	0	0	-1	0	0	-1	1	1	-1	-1	-1	1	0	0	0	0	0	-1	0	0	0	0	-1	-1	-1	1	0	0	-1	-1	0	0	CNA-high
387	0	1	0	0	0	0	0	0	0	-1	1	-1	0	0	0	0	-1	-1	0	1	0	0	0	0	0	0	0	0	0	0	0	0	0	-1	-1	0	0	0	0	0	0	CNA-Low
388	0	1	0	0	-1	1	1	-1	1	0	0	-1	1	1	-1	1	0	0	1	-1	0	0	-1	0	-1	0	0	-1	0	-1	0	0	-1	-1	0	-1	1	0	0	0	0	CNA-high
389	0	1	0	0	0	0	0	0	1	1	0	0	0	0	1	1	0	0	0	0	0	0	0	0	1	0	0	1	-1	-1	1	0	0	0	0	0	0	1	0	0	0	CNA-Low
390	0	1	0	0	0	0	-1	-1	0	0	-1	-1	0	0	-1	-1	-1	-1	-1	-1	0	0	-1	1	-1	0	-1	1	-1	-1	0	0	0	0	0	-1	0	0	-1	-1	-1	CNA-high
391	0	1	0	0	0	0	0	0	0	0	1	-1	0	0	0	0	0	0	0	0	0	0	0	0	0	0	1	1	-1	0	0	0	0	0	0	1	1	0	0	0	0	CNA-Low
392	-1	1	1	1	-1	0	0	-1	0	0	0	0	0	0	-1	0	-1	-1	1	-1	-1	0	-1	0	-1	0	0	0	0	-1	0	0	0	-1	-1	0	0	-1	-1	0	0	CNA-Low
393	0	0	0	0	0	0	0	0	0	0	1	-1	0	0	0	0	0	0	-1	0	0	0	0	0	-1	0	0	0	0	0	0	0	0	0	0	0	0	0	0	-1	1	CNA-Low
394	1	1	0	0	-1	-1	0	0	0	0	0	0	1	0	0	0	0	0	0	0	0	0	0	-1	-1	0	-1	0	0	0	0	-1	-1	-1	1	0	0	-1	0	0	0	CNA-Low
395	0	1	0	0	0	0	0	0	1	1	0	0	0	0	0	0	0	0	0	0	0	0	1	1	-1	1	-1	1	1	0	0	1	1	-1	-1	1	1	-1	-1	0	0	CNA-Low
396	-1	1	1	1	-1	0	-1	-1	1	1	0	1	1	1	-1	-1	-1	-1	-1	-1	1	1	-1	0	-1	1	-1	1	1	-1	1	0	0	-1	-1	-1	1	1	-1	0	0	CNA-high
397	0	1	0	0	0	0	0	0	1	1	1	-1	0	0	0	0	0	0	0	0	0	0	0	0	0	0	0	0	0	0	0	0	0	0	0	0	0	0	0	0	0	CNA-Low
398	0	1	0	0	0	0	0	0	0	0	0	0	0	0	0	0	0	0	0	0	0	0	0	0	1	0	0	0	0	0	0	0	0	-1	-1	0	0	0	0	1	-1	CNA-Low
399	0	1	0	-1	-1	0	1	1	1	0	1	-1	1	1	-1	1	0	0	-1	0	0	0	-1	0	-1	0	-1	0	0	-1	0	-1	-1	-1	-1	-1	-1	0	-1	0	0	CNA-high
400	1	1	0	-1	0	0	-1	1	1	-1	1	-1	1	1	0	0	-1	0	0	0	1	1	0	0	1	-1	-1	1	1	-1	0	1	1	0	0	0	0	-1	-1	-1	1	CNA-high

(Continued)

401	0	1	0	0	0	0	1	1	1	1	0	0	1	1	-1	0	0	-1	-1	-1	-1	1	0	0	0	-1	-1	1	0	-1	1	1	-1	-1	0	-1	1	1	-1	0	0	CNA-high
402	0	1	-1	0	0	0	0	0	0	1	0	0	0	0	0	0	0	0	0	0	0	0	0	0	0	0	0	1	1	-1	0	-1	-1	1	0	-1	0	0	0	0	0	CNA-Low
403	0	0	0	0	0	0	0	0	0	0	0	-1	0	0	0	0	0	0	0	0	0	0	-1	0	0	0	0	0	0	0	0	0	0	0	0	0	0	0	0	0	0	CNA-Low
404	-1	1	0	1	-1	1	0	0	1	0	0	-1	0	0	-1	1	0	0	0	0	0	0	1	0	0	1	-1	0	1	-1	0	0	0	0	0	0	0	0	0	0	0	CNA-Low
405	1	1	1	1	0	0	0	-1	1	-1	1	-1	0	1	0	1	0	-1	0	-1	1	1	-1	-1	-1	-1	-1	1	1	-1	0	0	0	-1	-1	1	1	-1	1	-1	1	CNA-high
406	0	0	1	1	-1	1	1	0	1	-1	0	0	0	-1	-1	0	-1	-1	0	0	1	1	1	1	0	0	1	0	1	-1	0	-1	-1	-1	1	-1	1	-1	0	0	0	CNA-high
407	0	1	1	0	1	-1	0	-1	1	1	1	-1	0	-1	-1	1	0	1	0	0	-1	-1	1	1	-1	-1	-1	0	-1	-1	1	0	0	0	0	-1	-1	1	0	1	0	CNA-high
408	-1	1	1	1	-1	1	1	-1	1	-1	1	-1	1	1	-1	1	-1	-1	1	1	-1	0	1	1	-1	-1	0	0	0	-1	-1	-1	-1	-1	-1	1	1	-1	1	-1	-1	CNA-high
409	1	1	0	-1	-1	1	1	1	0	-1	0	0	1	1	-1	1	-1	-1	0	-1	-1	1	-1	0	-1	0	-1	1	1	-1	1	1	0	-1	-1	-1	0	0	-1	-1	1	CNA-high
410	0	0	0	0	0	1	-1	-1	0	0	1	-1	0	0	-1	1	0	0	0	0	-1	1	0	0	-1	1	0	-1	-1	1	1	1	1	0	0	1	1	-1	-1	0	0	CNA-high
411	0	0	1	0	-1	-1	0	0	0	0	0	-1	0	0	-1	1	-1	0	1	0	0	0	1	1	-1	0	0	0	0	-1	0	0	1	0	0	0	1	1	-1	0	0	CNA-Low
412	0	1	1	1	0	0	1	1	1	1	1	0	1	1	1	1	0	0	0	0	0	0	0	0	1	-1	0	1	0	0	1	0	0	0	0	0	0	1	0	1	1	CNA-Low
413	0	0	1	0	-1	-1	0	0	1	0	0	0	1	-1	-1	1	-1	-1	-1	-1	1	1	-1	0	-1	1	-1	1	-1	-1	1	1	-1	-1	-1	1	-1	-1	1	-1	0	CNA-high
414	0	1	0	1	-1	0	-1	-1	-1	-1	0	-1	1	-1	-1	1	-1	-1	0	0	-1	1	-1	0	1	1	0	-1	-1	-1	0	-1	-1	0	0	1	1	-1	1	0	0	CNA-high
415	0	1	-1	1	-1	-1	0	0	-1	-1	0	0	1	1	-1	0	-1	-1	-1	-1	0	0	0	0	1	0	-1	1	1	-1	1	-1	-1	-1	-1	1	1	0	0	-1	0	CNA-high
416	0	0	0	0	0	0	0	0	1	0	1	-1	0	0	0	0	0	0	0	0	0	0	0	0	0	0	0	0	0	0	-1	0	0	0	0	0	-1	-1	0	0	-1	-1	CNA-Low
417	0	1	0	0	0	0	0	0	0	0	0	0	-1	-1	0	0	-1	-1	0	0	0	0	-1	0	0	-1	0	0	0	0	0	0	0	-1	0	0	0	0	0	0	0	CNA-Low
418	0	1	0	0	-1	-1	0	0	0	1	0	0	1	1	-1	1	-1	-1	0	0	-1	-1	-1	0	-1	1	0	0	0	0	0	0	-1	0	-1	0	0	1	0	0	0	CNA-Low
419	1	1	0	1	-1	1	-1	-1	1	1	1	-1	1	0	0	0	-1	-1	-1	-1	0	0	-1	-1	-1	0	-1	1	1	-1	0	0	0	-1	-1	1	1	0	0	0	0	CNA-high
420	0	0	0	0	-1	0	0	0	0	-1	0	0	0	0	0	1	-1	0	1	0	0	0	0	0	0	0	0	0	0	0	0	-1	1	-1	0	0	0	-1	-1	-1	-1	CNA-Low
421	0	1	0	1	0	0	0	0	1	0	0	0	1	1	1	1	0	1	1	0	0	0	0	0	-1	0	1	1	1	0	0	0	1	0	0	1	1	-1	0	1	1	CNA-Low
422	1	1	1	1	0	0	1	1	-1	-1	0	0	1	0	-1	1	-1	-1	0	0	-1	1	0	0	0	1	0	1	0	-1	1	-1	-1	0	0	0	0	1	1	0	1	CNA-high
423	0	0	0	0	0	0	0	0	0	0	0	0	0	0	0	0	0	0	0	0	0	0	0	0	0	0	0	0	0	0	0	0	0	0	0	0	0	0	0	0	0	CNA-Low
424	0	0	0	0	-1	0	0	0	0	0	0	-1	1	1	-1	1	-1	0	0	0	0	0	0	0	0	0	0	0	0	-1	0	-1	-1	0	0	0	0	0	0	-1	-1	CNA-Low
425	0	1	1	0	0	0	1	0	1	0	0	0	1	0	-1	1	0	1	0	0	0	0	0	1	0	0	1	1	1	0	0	0	0	0	1	1	0	0	0	1	1	CNA-Low
426	-1	1	0	0	0	0	0	0	1	0	0	0	0	0	0	1	0	0	0	0	0	0	0	0	-1	-1	0	0	0	-1	1	0	-1	0	0	0	0	0	0	0	0	CNA-Low
427	1	1	0	0	-1	0	-1	-1	1	-1	0	-1	1	0	-1	1	-1	0	1	1	-1	0	-1	0	-1	0	-1	-1	-1	0	1	-1	1	-1	-1	0	0	0	-1	0	1	CNA-high
428	0	0	0	0	0	1	-1	-1	1	0	0	0	0	0	-1	1	-1	-1	0	0	-1	0	-1	-1	-1	0	-1	0	1	-1	-1	-1	-1	-1	1	1	1	-1	-1	0	0	CNA-high
429	-1	1	0	0	-1	-1	0	0	1	1	1	0	0	0	0	0	-1	-1	0	0	0	0	0	-1	-1	-1	-1	1	1	1	1	1	1	-1	-1	0	0	-1	-1	-1	1	CNA-high
430	0	1	0	0	0	1	0	0	1	0	0	0	1	1	0	0	1	0	0	0	1	1	1	0	0	0	1	1	1	0	0	1	1	1	1	1	1	1	0	0	1	CNA-Low
431	0	1	0	0	0	0	0	0	0	-1	0	0	0	0	0	1	-1	-1	0	0	-1	1	0	0	-1	0	0	0	0	0	0	-1	-1	0	0	0	0	0	0	-1	0	CNA-Low
432	0	1	0	0	0	0	0	0	1	-1	0	0	1	1	0	0	-1	0	0	0	0	0	0	0	0	1	0	0	0	0	1	-1	-1	0	1	0	0	-1	0	0	0	CNA-Low
433	0	1	1	0	-1	0	0	0	1	0	0	-1	0	0	-1	0	-1	0	0	0	-1	0	0	1	-1	0	0	0	0	-1	0	0	0	-1	1	0	1	-1	-1	1	-1	CNA-Low
434	-1	1	0	0	-1	0	-1	-1	0	-1	-1	-1	1	0	1	1	0	0	1	-1	0	0	-1	1	0	-1	0	0	1	-1	0	-1	-1	-1	0	1	-1	-1	-1	0	0	CNA-high
435	0	1	0	0	-1	1	0	0	0	0	0	-1	0	0	-1	0	0	0	0	0	0	0	-1	-1	0	0	-1	0	0	-1	0	-1	-1	0	0	1	1	1	-1	0	0	CNA-Low
436	0	1	1	-1	0	0	0	0	1	-1	0	-1	0	1	-1	1	0	-1	0	0	0	0	1	0	-1	1	-1	1	0	-1	1	-1	-1	-1	-1	0	1	0	0	0	1	CNA-high
437	1	1	1	1	1	1	1	0	1	0	0	0	1	1	0	0	1	0	-1	-1	0	0	1	-1	0	1	-1	0	0	-1	0	0	0	0	0	-1	1	0	0	-1	1	CNA-high
438	0	0	0	0	0	0	0	0	0	0	0	0	0	0	0	0	0	0	0	0	0	0	0	0	0	0	0	0	0	0	0	0	0	0	0	0	0	0	0	0	0	CNA-Low
439	-1	1	0	0	-1	0	1	0	0	-1	0	-1	1	0	-1	0	-1	-1	1	-1	-1	0	0	0	0	0	1	-1	-1	-1	-1	-1	-1	0	0	1	1	-1	0	0	-1	CNA-high
440	0	1	1	0	0	0	0	-1	1	1	0	-1	1	0	-1	1	0	-1	0	-1	0	1	0	0	-1	0	-1	1	0	-1	1	0	0	0	0	0	0	1	-1	-1	1	CNA-high

(Continued)

441	-1	1	0	0	-1	-1	-1	-1	-1	-1	1	1	-1	-1	-1	1	-1	-1	0	0	0	0	-1	0	1	0	-1	1	1	-1	1	-1	-1	0	0	1	1	-1	0	0	0	CNA-high
442	-1	1	1	1	0	1	0	0	0	0	-1	-1	1	1	1	1	-1	-1	0	0	0	0	-1	0	-1	0	0	0	0	0	1	-1	-1	-1	0	1	1	0	0	0	0	CNA-high
443	0	0	1	0	-1	1	0	0	-1	-1	1	0	0	1	0	1	-1	-1	1	1	0	-1	1	1	-1	0	-1	-1	-1	-1	1	1	-1	-1	0	0	0	-1	-1	0	0	CNA-high
444	0	0	1	0	0	0	-1	-1	1	-1	0	-1	1	1	-1	0	0	0	0	0	0	0	0	0	-1	1	-1	0	-1	-1	0	-1	-1	-1	0	0	0	-1	-1	0	1	CNA-Low
445	1	0	0	1	-1	0	0	1	1	0	0	0	1	1	-1	0	-1	-1	-1	-1	0	0	-1	-1	0	0	-1	0	0	-1	0	1	-1	-1	0	1	1	1	0	0	0	CNA-high
446	0	0	0	0	0	0	0	0	0	0	0	0	1	0	0	0	-1	-1	0	0	0	0	-1	-1	0	0	0	0	-1	-1	0	0	0	0	0	0	0	0	0	0	0	CNA-Low
447	0	0	0	0	0	0	0	0	1	1	0	0	0	0	0	0	0	0	-1	-1	0	0	0	0	0	0	0	0	0	0	0	0	0	0	0	0	0	0	1	0	0	CNA-Low
448	-1	1	1	0	-1	1	0	0	1	0	1	-1	0	1	-1	1	1	0	1	-1	1	-1	-1	0	0	0	-1	0	-1	-1	0	0	-1	0	0	0	0	-1	-1	0	0	CNA-high
449	0	0	0	0	0	0	0	0	1	0	0	0	1	1	-1	1	0	0	-1	-1	0	0	0	0	0	0	-1	1	-1	-1	1	0	0	0	0	0	0	0	0	0	0	CNA-Low
450	0	1	0	0	0	0	1	1	1	1	0	0	1	1	0	0	0	0	0	0	0	0	0	0	0	0	0	1	-1	0	0	0	0	0	0	0	0	0	-1	0	0	CNA-Low
451	0	1	1	1	-1	1	0	-1	0	-1	0	-1	1	0	0	-1	0	-1	0	0	-1	0	1	0	1	0	-1	0	-1	0	1	0	0	-1	0	0	0	-1	0	0	0	CNA-Low
452	-1	1	1	1	-1	1	0	0	1	-1	-1	-1	1	-1	-1	1	0	-1	1	1	-1	-1	1	1	-1	1	0	-1	-1	-1	1	0	0	-1	-1	1	1	-1	-1	0	0	CNA-high
453	0	1	1	1	0	1	1	-1	1	0	-1	-1	1	0	-1	1	-1	0	0	0	0	0	1	1	-1	0	0	1	1	-1	1	-1	-1	-1	-1	-1	0	-1	-1	0	0	CNA-high
454	0	1	0	0	-1	1	-1	-1	0	0	0	0	0	0	0	0	0	0	0	-1	0	0	-1	0	0	0	0	0	0	-1	0	0	0	0	-1	0	1	0	0	-1	1	CNA-Low
455	-1	0	0	0	0	0	-1	-1	1	-1	0	0	1	1	-1	1	0	0	0	0	0	0	0	0	-1	-1	-1	0	-1	-1	1	-1	-1	-1	-1	-1	0	-1	0	0	0	CNA-high
456	1	1	0	0	-1	1	-1	-1	-1	-1	0	0	1	1	-1	1	-1	-1	-1	-1	1	1	1	1	-1	1	-1	0	-1	-1	1	1	-1	-1	-1	1	-1	-1	0	0	0	CNA-high
457	0	0	0	0	0	0	0	0	0	0	0	0	0	0	0	0	0	0	0	0	0	0	0	0	0	0	1	0	0	0	0	0	0	0	0	0	0	0	0	0	0	CNA-Low
458	0	0	1	1	0	0	0	0	0	0	0	0	1	1	1	1	-1	-1	0	0	0	0	1	1	0	-1	1	-1	-1	1	1	-1	-1	-1	1	0	0	1	-1	0	0	CNA-high
459	1	-1	1	0	-1	1	-1	0	1	-1	1	-1	1	1	-1	1	-1	-1	1	-1	1	1	1	1	-1	0	1	-1	-1	-1	0	1	1	0	-1	0	1	-1	0	0	0	CNA-high
460	-1	0	0	0	0	0	0	0	1	0	0	0	0	0	0	0	0	0	1	1	0	0	0	0	0	0	0	0	0	0	0	0	-1	-1	0	-1	-1	-1	0	0	0	CNA-Low
461	0	0	0	0	0	0	0	0	1	0	0	0	0	0	0	0	0	0	0	0	0	0	-1	-1	0	0	0	0	0	-1	0	-1	-1	0	0	0	0	0	-1	-1	1	CNA-Low
462	0	1	0	1	0	0	0	0	1	-1	1	-1	0	0	0	0	0	0	1	1	0	0	0	1	-1	0	0	0	0	-1	1	1	0	0	0	1	0	-1	0	0	0	CNA-Low
463	1	1	0	1	-1	0	1	0	0	-1	0	1	0	0	1	1	0	-1	1	1	0	0	-1	1	-1	0	-1	0	-1	1	1	0	0	-1	1	0	1	0	-1	0	0	CNA-high
464	0	0	0	0	-1	1	0	-1	0	0	0	0	0	0	0	0	0	0	0	0	-1	0	0	0	0	0	0	0	0	0	0	-1	-1	0	-1	0	0	0	0	-1	0	CNA-Low
465	0	1	0	-1	0	0	-1	0	0	-1	1	1	0	1	-1	1	0	-1	1	0	1	-1	0	0	-1	0	-1	0	-1	-1	0	-1	0	1	-1	0	1	0	1	0	0	CNA-high
466	0	0	0	0	0	1	0	0	1	0	0	0	0	0	0	0	0	0	0	0	0	0	0	0	0	0	0	0	0	0	0	0	0	0	0	0	0	0	0	0	0	CNA-Low
467	0	0	0	0	-1	0	0	0	1	0	0	0	1	1	0	0	-1	-1	-1	0	0	0	0	0	0	1	-1	1	1	0	0	-1	-1	0	0	1	1	-1	0	0	0	CNA-Low
468	0	1	0	0	-1	1	1	-1	1	-1	1	1	1	1	-1	1	-1	-1	-1	-1	0	0	0	0	-1	-1	0	1	-1	1	1	-1	-1	-1	0	0	1	1	-1	0	0	CNA-high
469	0	1	0	0	0	0	1	1	1	0	0	0	1	1	-1	1	0	0	1	0	0	1	0	0	-1	1	0	1	1	0	1	0	0	0	0	-1	1	1	0	0	1	CNA-Low
470	0	0	1	0	0	1	0	0	0	-1	0	0	-1	-1	0	0	0	-1	-1	0	0	0	0	0	0	0	-1	0	-1	-1	0	0	0	0	0	1	0	0	0	0	0	CNA-Low
471	0	0	0	0	0	0	0	0	0	0	0	0	1	1	0	1	0	0	1	-1	0	0	-1	0	0	0	0	0	0	-1	1	0	0	0	0	0	0	0	0	0	0	CNA-Low
472	0	1	0	0	0	0	0	0	0	0	0	-1	0	0	0	1	0	0	0	0	0	0	0	0	0	0	1	0	0	0	0	0	0	0	0	0	0	0	0	0	0	CNA-Low
473	-1	1	0	0	0	0	0	0	0	0	0	-1	1	1	0	0	-1	-1	0	-1	0	0	0	0	0	0	0	-1	-1	0	0	0	0	0	0	1	1	0	0	0	0	CNA-Low
474	1	1	0	0	-1	0	-1	-1	1	1	0	0	1	0	-1	1	-1	-1	-1	0	0	0	-1	-1	-1	0	-1	0	0	-1	1	-1	-1	-1	0	0	0	1	-1	0	0	CNA-high
475	1	1	0	1	-1	0	0	0	1	-1	0	-1	1	1	-1	0	-1	-1	-1	-1	-1	0	0	0	-1	0	-1	1	1	-1	1	-1	-1	-1	-1	-1	0	1	-1	0	0	CNA-high
476	-1	1	0	0	-1	-1	-1	-1	0	0	0	0	1	-1	0	0	-1	-1	0	0	-1	-1	0	0	-1	0	1	0	0	0	0	-1	-1	0	0	1	-1	-1	-1	0	0	CNA-Low
477	-1	0	0	1	-1	1	0	-1	0	-1	0	-1	0	0	-1	0	0	0	0	0	-1	0	1	0	-1	-1	0	1	0	-1	1	0	0	-1	-1	0	-1	-1	-1	0	0	CNA-Low
478	1	1	0	1	1	-1	0	0	1	0	1	-1	1	0	-1	1	-1	-1	0	0	-1	-1	1	0	-1	-1	-1	-1	0	-1	0	0	1	-1	1	1	1	-1	-1	0	0	CNA-high
479	-1	1	0	0	-1	-1	-1	-1	0	0	-1	-1	-1	1	-1	1	1	-1	-1	0	-1	0	0	0	-1	1	-1	0	0	-1	0	0	0	-1	-1	0	0	-1	-1	0	0	CNA-high
480	0	1	1	1	0	-1	0	-1	0	0	1	0	1	1	-1	1	-1	-1	1	-1	1	0	-1	0	-1	-1	-1	0	-1	0	1	1	-1	0	-1	0	0	0	-1	0	0	CNA-high

(Continued)

481	-1	1	0	0	-1	1	0	0	0	0	1	-1	0	1	0	0	1	1	-1	-1	0	1	1	1	0	1	-1	0	0	1	1	-1	-1	-1	-1	-1	1	-1	0	-1	1	CNA-high
482	0	1	1	0	-1	0	0	0	1	-1	1	0	1	0	-1	-1	-1	-1	1	-1	0	1	-1	0	0	0	-1	1	1	-1	1	1	1	-1	0	1	1	-1	-1	0	0	CNA-high
483	-1	1	0	0	-1	0	-1	-1	0	-1	1	0	1	1	-1	1	-1	-1	1	1	-1	0	1	0	0	0	1	0	0	-1	0	-1	-1	-1	-1	-1	1	-1	-1	-1	1	CNA-high
484	-1	1	1	1	-1	-1	-1	-1	1	1	-1	-1	1	1	1	1	-1	-1	-1	1	-1	-1	1	1	-1	-1	-1	1	-1	1	1	1	1	1	1	1	0	-1	1	0	0	CNA-high
485	-1	1	0	0	0	0	0	0	0	0	1	1	0	0	1	1	0	0	1	1	0	0	-1	-1	1	1	0	0	0	0	0	0	0	-1	-1	1	1	1	0	1	1	CNA-Low
486	-1	1	0	0	-1	-1	-1	-1	0	0	0	0	0	0	0	1	-1	-1	1	1	1	0	1	1	0	0	1	0	0	-1	0	0	0	0	0	1	0	-1	-1	0	0	CNA-Low
487	0	1	0	0	0	0	1	0	1	1	0	0	1	1	0	1	0	0	0	0	1	1	0	0	-1	0	0	1	1	0	0	0	-1	0	0	-1	1	1	0	1	1	CNA-Low
488	-1	1	1	0	-1	1	0	-1	1	-1	1	-1	1	1	-1	0	-1	-1	1	1	-1	0	0	1	-1	-1	0	0	0	0	0	1	-1	-1	0	1	1	0	-1	0	0	CNA-high
489	1	1	0	1	1	0	0	0	1	-1	-1	-1	1	1	-1	1	0	-1	0	0	-1	0	1	-1	1	0	-1	0	-1	-1	0	1	0	-1	1	-1	1	1	-1	-1	0	CNA-high
490	1	1	0	0	0	1	0	0	0	0	0	-1	0	0	0	0	0	0	-1	-1	1	1	0	0	0	0	0	0	0	-1	0	0	-1	0	0	0	0	-1	0	0	0	CNA-Low
491	0	1	0	0	-1	-1	0	0	1	-1	0	0	0	0	0	0	-1	-1	-1	-1	0	0	0	0	0	0	0	0	0	0	0	-1	-1	0	-1	0	0	0	0	-1	-1	CNA-Low
492	0	1	0	1	-1	1	-1	-1	1	1	1	1	0	1	1	1	0	-1	0	0	-1	-1	1	1	-1	1	-1	0	0	1	0	-1	-1	-1	1	1	1	0	-1	0	1	CNA-high
493	-1	-1	0	0	-1	1	-1	-1	0	-1	-1	0	-1	0	-1	0	0	0	0	0	0	-1	1	0	-1	-1	0	-1	-1	-1	0	0	0	0	0	0	0	-1	-1	-1	-1	CNA-high
494	0	1	0	0	0	0	1	0	1	1	0	0	1	1	0	0	0	0	0	0	0	1	0	0	0	0	1	1	0	0	0	0	0	0	0	1	1	0	0	1	1	CNA-Low
495	1	1	0	0	0	0	0	0	1	-1	0	0	1	1	0	0	0	0	0	0	0	0	1	1	0	0	-1	0	0	0	0	0	0	0	0	1	0	-1	0	0	0	CNA-Low
496	0	1	0	0	-1	0	-1	-1	1	-1	0	0	1	1	-1	1	0	0	1	-1	0	0	-1	-1	0	0	-1	0	-1	-1	0	0	0	-1	1	1	1	0	0	0	0	CNA-high
497	0	0	0	0	0	0	0	0	0	0	0	0	1	0	0	0	-1	-1	0	0	0	0	0	0	0	0	0	0	0	0	0	0	0	0	0	0	0	0	0	0	0	CNA-Low
498	0	1	1	0	0	0	-1	-1	1	-1	0	0	1	0	-1	1	0	0	0	0	0	-1	0	0	-1	0	-1	-1	-1	0	0	0	-1	-1	0	1	1	0	0	0	0	CNA-Low
499	1	1	0	0	-1	-1	0	0	1	-1	-1	-1	0	0	-1	1	-1	-1	1	1	0	0	-1	-1	-1	-1	0	0	-1	-1	0	1	-1	0	0	1	1	-1	0	0	0	CNA-high
500	-1	0	0	-1	-1	0	0	-1	0	0	0	0	1	-1	0	0	-1	-1	0	0	-1	-1	0	0	-1	-1	1	0	0	-1	0	-1	-1	-1	1	-1	-1	0	-1	-1	-1	CNA-high
501	0	0	0	0	-1	0	0	0	0	0	0	0	-1	0	-1	0	-1	0	0	0	-1	0	-1	0	0	0	0	0	-1	0	0	0	0	-1	-1	-1	0	0	-1	0	0	CNA-Low
502	-1	0	0	0	0	-1	0	0	1	-1	0	0	1	0	-1	1	0	0	0	-1	-1	0	-1	0	-1	1	-1	-1	0	-1	1	0	0	-1	-1	1	0	-1	-1	0	1	CNA-high
503	-1	1	1	0	-1	-1	0	0	1	0	1	-1	0	0	0	0	0	-1	-1	-1	-1	-1	0	0	0	1	-1	-1	-1	-1	1	0	0	-1	0	0	1	-1	-1	0	-1	CNA-high
504	0	0	0	0	1	0	0	0	0	1	0	-1	0	0	-1	1	1	0	-1	1	1	1	1	0	0	1	0	0	0	0	0	1	-1	0	0	1	1	-1	0	1	0	CNA-Low
505	-1	1	0	0	0	0	0	-1	1	0	0	0	0	0	-1	0	0	0	0	-1	-1	-1	-1	-1	0	-1	-1	0	-1	-1	0	0	-1	-1	1	0	0	0	-1	-1	-1	CNA-Low
506	0	1	0	0	-1	0	0	0	-1	-1	1	-1	1	-1	1	1	0	-1	-1	-1	0	1	0	1	-1	0	-1	-1	0	0	0	0	1	-1	1	0	0	-1	-1	0	0	CNA-high
507	1	1	0	0	-1	0	0	0	1	0	-1	-1	1	1	0	0	0	0	0	0	-1	0	0	0	-1	-1	0	0	0	0	0	-1	-1	0	1	-1	1	-1	-1	0	0	CNA-Low
508	0	0	0	0	-1	1	-1	0	0	0	0	-1	0	0	-1	0	0	0	0	0	0	0	0	0	0	0	0	0	0	-1	0	-1	-1	0	0	0	0	0	0	-1	0	CNA-Low
509	0	0	1	1	-1	-1	1	0	1	-1	0	0	-1	0	0	0	0	-1	0	0	0	0	0	0	-1	-1	-1	-1	-1	0	0	0	0	-1	0	0	0	0	0	0	0	CNA-Low
510	1	1	1	-1	-1	1	0	1	1	0	0	0	0	0	-1	1	0	0	-1	-1	0	0	0	0	-1	0	0	0	0	-1	0	1	-1	-1	-1	0	0	1	-1	0	0	CNA-Low
511	0	1	0	0	0	0	-1	1	1	0	0	-1	1	0	-1	0	-1	-1	1	-1	0	0	-1	-1	-1	1	-1	0	0	-1	1	0	0	-1	-1	-1	1	1	-1	0	0	CNA-high
512	0	0	0	0	-1	1	0	0	1	-1	0	-1	1	1	0	0	0	0	-1	-1	0	0	-1	0	-1	1	0	-1	-1	0	0	-1	-1	0	0	1	1	0	-1	0	0	CNA-Low

Chromosomal arm-level copy number status is shown as follows: 0, diploid; 1, chromosomal gain; -1, chromosomal deletion.

Supplementary Table S3. Chromosomal arm-level copy number status in 498 LUSC cases.

Case No.	1p	1q	2p	2q	3p	3q	4p	4q	5p	5q	6p	6q	7p	7q	8p	8q	9p	9q	10p	10q	11p	11q	12p	12q	13q	14q	15q	16p	16q	17p	17q	18p	18q	19p	19q	20p	20q	21q	22q	Xp	Xq	CNA status
1	-1	1	0	0	-1	1	-1	-1	1	-1	0	0	1	0	-1	0	-1	0	0	-1	0	0	0	0	0	1	-1	-1	-1	0	0	0	0	-1	-1	1	0	0	0	-1	-1	CNA-low
2	0	0	0	0	0	1	0	0	0	0	0	0	0	0	-1	1	0	0	-1	0	0	0	0	0	0	0	0	1	1	0	0	0	0	0	0	0	0	0	0	-1	-1	CNA-low
3	0	0	0	0	-1	1	0	0	1	-1	0	0	0	0	-1	0	0	0	0	0	-1	0	1	0	0	0	0	0	0	-1	0	0	-1	0	0	0	0	0	0	0	0	CNA-low
4	0	1	0	0	0	1	0	0	1	-1	0	0	1	0	0	1	0	0	0	0	0	0	0	0	0	-1	0	0	0	0	0	1	1	0	0	1	1	0	1	0	0	CNA-low
5	-1	1	1	-1	-1	1	-1	-1	1	-1	1	-1	-1	0	-1	1	-1	-1	-1	1	-1	1	1	1	0	1	0	-1	1	-1	1	1	-1	-1	1	1	1	-1	1	0	0	CNA-high
6	0	1	0	-1	-1	1	-1	-1	1	0	1	-1	-1	1	-1	1	-1	0	1	1	0	-1	-1	1	-1	0	-1	1	0	-1	1	-1	-1	-1	1	-1	1	-1	-1	0	0	CNA-high
7	-1	1	1	0	-1	0	-1	-1	1	-1	0	0	1	1	-1	0	-1	0	-1	0	-1	0	1	1	-1	1	-1	0	0	0	1	1	0	0	0	1	0	-1	0	0	-1	CNA-high
8	0	0	0	0	-1	1	-1	-1	1	-1	0	0	1	1	0	1	-1	0	0	0	0	0	0	0	-1	0	1	0	0	-1	-1	0	0	0	0	1	1	0	0	0	0	CNA-low
9	-1	1	1	0	-1	0	-1	-1	1	-1	-1	0	1	1	-1	1	0	1	1	-1	-1	-1	1	0	-1	-1	0	0	-1	-1	1	0	-1	0	0	1	0	-1	1	-1	0	CNA-high
10	-1	1	1	0	-1	0	-1	-1	1	-1	0	0	0	0	0	1	1	-1	-1	-1	0	0	0	0	0	-1	1	0	0	1	1	0	0	1	0	0	0	-1	0	0	0	CNA-low
11	-1	0	1	-1	-1	1	0	0	1	-1	-1	1	1	1	-1	1	-1	1	-1	-1	-1	-1	1	0	-1	-1	0	0	0	-1	1	0	0	1	1	1	0	-1	1	-1	1	CNA-high
12	0	0	1	0	-1	0	1	1	1	-1	-1	-1	1	1	-1	1	-1	-1	-1	-1	-1	-1	-1	-1	-1	0	0	-1	-1	-1	0	-1	1	-1	0	1	1	-1	0	0	0	CNA-high
13	0	1	1	0	-1	1	0	0	0	0	0	0	0	0	-1	0	0	0	0	0	0	0	1	1	0	-1	-1	-1	-1	-1	0	1	1	0	0	1	1	-1	0	-1	1	CNA-low
14	0	0	0	0	-1	0	-1	-1	1	-1	0	1	1	0	1	1	-1	1	-1	-1	0	0	1	0	-1	0	1	-1	0	0	0	-1	0	0	0	1	0	-1	1	0	0	CNA-low
15	0	1	0	0	-1	0	-1	-1	1	-1	0	0	0	0	-1	1	-1	0	0	-1	0	0	0	0	-1	0	0	-1	0	0	0	0	0	0	1	1	1	0	0	-1	-1	CNA-low
16	0	1	1	1	0	1	-1	-1	0	0	0	0	0	0	1	1	0	0	0	1	0	0	1	0	0	0	0	0	-1	-1	0	1	1	0	1	0	0	0	0	0	0	CNA-low
17	0	0	1	0	-1	1	-1	-1	0	-1	-1	0	1	0	-1	0	-1	0	-1	-1	0	1	1	1	-1	-1	0	0	0	-1	1	1	1	0	0	1	0	-1	1	0	-1	CNA-high
18	0	0	0	0	-1	1	0	0	1	0	0	-1	-1	1	-1	0	0	0	0	0	0	0	0	0	-1	0	0	0	0	-1	0	0	0	-1	0	0	0	-1	-1	0	0	CNA-low
19	0	0	0	0	0	0	0	0	0	0	0	0	1	0	0	0	0	0	0	0	0	0	0	0	0	0	0	0	0	0	0	0	0	0	0	0	0	0	0	0	0	CNA-low
20	-1	0	-1	0	-1	1	-1	-1	1	-1	-1	1	1	-1	0	1	-1	0	0	-1	0	1	0	1	-1	1	-1	0	0	-1	0	0	0	-1	1	1	1	-1	0	0	1	CNA-high
21	0	0	1	1	0	0	0	0	1	-1	0	0	1	-1	-1	1	-1	1	1	-1	0	1	-1	-1	-1	0	-1	1	-1	-1	1	1	-1	0	1	0	1	-1	0	0	0	CNA-high
22	0	0	0	1	-1	1	0	0	-1	-1	0	0	0	0	-1	1	-1	-1	0	0	0	0	0	0	1	-1	0	0	0	-1	0	-1	-1	-1	-1	0	0	-1	0	0	0	CNA-low
23	-1	1	1	1	-1	1	-1	-1	1	-1	1	0	-1	1	-1	0	-1	-1	-1	-1	0	1	1	-1	-1	1	0	0	0	-1	-1	1	1	-1	1	1	1	-1	1	0	0	CNA-high
24	-1	0	0	0	0	1	1	1	1	-1	-1	0	1	1	0	0	-1	-1	-1	-1	-1	0	1	1	-1	0	1	1	1	-1	1	-1	-1	0	1	-1	1	-1	1	-1	1	CNA-high
25	-1	1	1	1	-1	1	-1	-1	1	-1	0	1	0	1	-1	1	-1	-1	-1	-1	-1	0	0	0	-1	-1	1	0	0	-1	1	1	1	1	1	1	1	-1	-1	-1	1	CNA-high
26	0	0	0	0	0	0	-1	-1	1	-1	0	0	0	0	0	1	-1	-1	1	0	-1	1	1	1	0	1	0	-1	-1	0	0	0	-1	-1	1	0	0	0	-1	0	0	CNA-low
27	-1	1	1	0	-1	0	0	0	-1	-1	0	-1	1	1	-1	1	-1	-1	0	0	-1	1	1	1	-1	0	1	0	-1	-1	1	1	-1	1	1	1	1	1	1	0	0	CNA-high
28	0	0	0	0	-1	1	0	0	0	0	0	0	0	0	1	1	-1	0	0	0	-1	-1	0	0	-1	0	0	0	0	0	0	0	0	0	0	0	0	0	0	0	0	CNA-low
29	0	0	1	1	-1	1	-1	-1	1	-1	0	0	1	1	-1	0	0	0	-1	-1	-1	-1	0	0	0	0	0	-1	-1	0	0	1	0	-1	0	0	1	-1	1	0	0	CNA-low
30	0	1	0	0	-1	0	0	0	1	-1	0	0	0	0	1	1	1	1	0	0	1	0	1	1	0	0	0	-1	-1	0	0	1	1	1	1	1	1	0	1	0	0	CNA-low
31	0	0	0	0	-1	1	-1	-1	0	-1	0	0	0	0	0	0	-1	-1	-1	-1	0	0	0	0	-1	-1	0	0	-1	0	0	0	0	0	0	0	0	-1	0	-1	1	CNA-low
32	0	1	1	0	-1	1	-1	-1	1	0	1	-1	1	1	-1	1	0	-1	1	1	-1	0	1	0	-1	-1	0	-1	1	0	0	0	0	-1	1	1	1	0	1	1	1	CNA-high
33	0	0	0	0	-1	1	-1	-1	1	-1	0	0	0	0	0	0	-1	0	0	-1	-1	0	0	0	-1	-1	1	0	-1	0	0	0	0	-1	-1	0	0	0	0	-1	-1	CNA-low
34	0	0	1	1	-1	1	-1	-1	0	0	1	1	1	1	-1	1	0	-1	-1	-1	-1	-1	1	1	0	0	0	0	-1	0	1	1	1	0	1	1	1	-1	1	1	1	CNA-high
35	1	1	0	0	-1	1	0	0	1	0	1	0	0	0	0	1	0	0	0	0	-1	-1	0	0	-1	0	0	0	0	0	0	0	0	0	0	0	0	0	0	0	0	CNA-low
36	0	1	0	0	-1	1	0	0	1	-1	0	0	1	0	-1	0	0	-1	0	0	0	0	0	0	0	-1	0	0	-1	-1	0	0	0	0	0	0	0	0	0	0	0	CNA-low
37	-1	0	1	0	-1	1	-1	-1	0	-1	-1	-1	0	1	-1	0	-1	0	-1	-1	-1	-1	0	0	0	0	0	-1	-1	-1	1	1	-1	0	0	0	0	-1	0	-1	-1	CNA-high
38	0	1	1	0	-1	1	-1	-1	1	1	-1	-1	0	0	-1	1	1	-1	-1	-1	-1	1	-1	-1	0	1	0	0	1	-1	0	0	1	-1	1	1	1	1	0	0	0	CNA-high
39	0	0	0	0	0	0	0	0	1	-1	0	0	1	1	-1	1	0	-1	0	0	0	0	0	0	-1	0	0	0	0	0	0	-1	0	0	0	0	0	0	1	0	0	CNA-low
40	0	0	0	0	-1	0	0	0	0	-1	1	1	0	-1	-1	0	-1	0	-1	-1	0	0	1	0	-1	-1	0	0	0	0	0	0	0	0	-1	0	0	0	1	-1	0	CNA-low

(Continued)

41	0	0	0	0	1	1	-1	-1	0	0	0	0	1	-1	-1	-1	-1	-1	0	0	1	0	1	1	0	1	0	0	0	0	0	0	0	0	0	-1	1	-1	-1	0	0	CNA-low
42	0	1	1	0	0	0	0	0	1	-1	0	-1	0	1	-1	0	1	0	1	-1	-1	0	1	1	-1	1	0	0	0	-1	1	0	0	-1	1	1	-1	-1	1	0	0	CNA-high
43	-1	-1	0	0	-1	1	-1	-1	1	-1	1	1	-1	-1	0	0	0	-1	0	0	1	-1	1	0	0	-1	-1	0	-1	-1	1	1	1	1	-1	1	1	-1	0	0	0	CNA-high
44	-1	1	0	0	-1	0	-1	-1	1	0	1	-1	0	0	0	1	-1	-1	0	0	0	1	1	0	-1	1	0	-1	1	0	0	-1	-1	0	1	1	1	0	1	0	0	CNA-high
45	0	0	1	1	0	0	0	0	1	-1	1	1	-1	1	0	1	-1	-1	0	0	0	0	1	1	-1	1	0	0	0	0	0	0	0	-1	1	0	1	-1	-1	0	0	CNA-low
46	0	0	0	0	-1	1	-1	-1	0	-1	0	0	1	1	0	0	0	0	-1	-1	0	0	0	0	0	-1	0	0	0	0	0	0	0	0	1	0	0	0	0	0	0	CNA-low
47	0	0	1	0	0	0	0	0	1	-1	1	-1	1	1	-1	1	0	0	1	1	-1	0	0	0	-1	-1	0	-1	-1	-1	0	0	0	-1	0	0	0	-1	-1	0	0	CNA-low
48	0	0	1	0	-1	1	-1	-1	0	0	0	0	1	1	-1	0	0	0	0	-1	0	0	1	0	0	-1	-1	1	1	-1	0	1	-1	0	1	1	1	1	0	0	0	CNA-low
49	-1	1	1	0	-1	0	-1	-1	1	-1	0	-1	0	1	-1	0	-1	1	-1	-1	-1	1	0	1	0	1	0	-1	1	1	1	1	-1	0	-1	1	-1	-1	0	0	1	CNA-high
50	-1	-1	1	1	-1	0	0	0	1	1	1	0	1	0	-1	1	0	0	0	0	0	0	0	0	-1	1	-1	1	1	0	0	-1	-1	0	1	1	1	0	1	-1	1	CNA-high
51	1	1	1	1	-1	1	-1	-1	1	-1	1	1	-1	-1	-1	1	-1	-1	1	-1	1	0	1	-1	0	-1	-1	1	1	-1	1	-1	-1	-1	-1	-1	1	0	-1	0	0	CNA-high
52	-1	0	0	0	-1	1	0	0	-1	-1	0	0	1	1	-1	1	1	0	1	-1	-1	0	0	0	-1	1	1	0	-1	0	0	1	-1	1	-1	0	1	-1	1	-1	1	CNA-high
53	-1	1	1	0	-1	1	-1	-1	0	-1	-1	-1	1	1	1	1	0	0	-1	-1	-1	0	0	0	0	0	0	0	0	0	0	0	0	0	0	0	0	-1	1	0	0	CNA-low
54	1	1	0	-1	-1	0	0	0	1	0	0	0	1	-1	-1	1	1	0	1	0	-1	0	0	0	-1	-1	0	0	-1	-1	0	0	1	0	1	0	0	0	1	0	0	CNA-low
55	0	1	1	0	-1	1	1	1	1	-1	0	0	-1	1	-1	-1	-1	-1	0	-1	-1	0	1	0	-1	0	0	0	0	-1	1	-1	-1	-1	1	1	1	0	1	0	0	CNA-high
56	-1	1	0	1	-1	1	-1	-1	1	-1	0	1	0	1	1	0	1	1	1	0	0	0	1	1	-1	0	0	1	1	0	0	-1	0	1	-1	1	0	-1	0	-1	0	CNA-high
57	0	0	0	0	0	0	-1	-1	0	-1	0	0	1	1	0	0	0	0	0	0	0	0	0	0	0	0	0	0	0	0	0	0	0	0	0	1	0	0	0	0	0	CNA-low
58	0	0	0	0	-1	1	-1	-1	1	-1	-1	-1	1	0	0	1	-1	-1	-1	-1	1	1	1	0	-1	1	1	1	1	-1	-1	1	1	-1	1	1	1	-1	1	0	0	CNA-high
59	0	0	0	0	-1	1	0	0	0	0	-1	0	1	1	0	0	0	0	0	0	0	0	0	0	-1	0	0	0	0	-1	0	0	0	0	0	0	0	-1	0	0	0	CNA-low
60	0	0	0	0	-1	1	0	0	1	-1	0	0	0	0	0	0	0	-1	0	-1	0	0	0	0	-1	1	-1	0	0	0	0	0	0	1	1	0	0	0	0	0	0	CNA-low
61	-1	0	1	0	-1	1	-1	-1	-1	-1	0	0	0	0	-1	1	0	0	0	0	0	0	1	0	0	-1	0	0	-1	-1	0	1	0	0	-1	0	0	-1	1	-1	-1	CNA-low
62	0	0	1	1	-1	1	0	0	1	-1	0	0	0	0	-1	0	1	-1	-1	-1	1	1	1	1	-1	-1	-1	-1	-1	-1	1	-1	-1	0	1	1	0	-1	1	-1	1	CNA-high
63	0	1	0	0	-1	1	-1	-1	0	0	0	0	0	0	-1	0	-1	-1	0	0	0	0	0	0	0	-1	0	0	0	0	0	0	0	0	1	0	0	0	0	0	0	CNA-low
64	0	0	0	0	-1	1	0	0	1	-1	0	0	0	0	-1	1	-1	-1	0	0	-1	0	0	0	-1	0	0	0	0	-1	0	0	0	0	0	1	0	1	0	0	0	CNA-low
65	0	1	1	0	-1	1	0	0	1	-1	0	0	1	1	-1	0	-1	-1	-1	0	1	1	0	0	0	1	0	0	1	-1	1	1	0	-1	-1	1	1	0	1	0	1	CNA-high
66	-1	0	0	0	-1	0	0	0	0	-1	-1	0	0	0	-1	0	-1	0	0	-1	0	0	0	0	0	1	0	0	-1	0	0	0	0	0	0	1	-1	-1	0	-1	-1	CNA-low
67	0	1	1	0	-1	1	-1	-1	1	-1	1	1	1	1	-1	1	0	0	-1	-1	0	-1	-1	-1	0	-1	1	-1	-1	0	0	1	-1	1	0	0	0	0	-1	0	0	CNA-high
68	0	0	0	0	0	0	0	0	0	0	0	0	0	0	0	0	0	0	0	0	0	0	0	0	0	0	0	0	0	0	0	0	0	0	0	0	0	0	0	0	0	CNA-low
69	0	0	0	0	-1	1	0	0	0	-1	0	0	0	0	0	0	-1	-1	-1	0	0	0	0	0	0	0	0	0	0	-1	0	0	-1	0	0	0	0	0	1	0	0	CNA-low
70	-1	0	0	0	-1	1	-1	-1	1	-1	0	0	1	1	0	0	0	-1	0	-1	0	-1	0	0	0	0	0	0	-1	0	0	-1	-1	0	-1	0	0	1	1	-1	0	CNA-low
71	0	0	1	1	-1	0	-1	-1	1	-1	0	0	0	0	-1	0	-1	-1	1	-1	-1	-1	1	0	-1	0	1	1	-1	0	1	1	-1	-1	-1	1	1	-1	-1	0	-1	CNA-high
72	-1	1	1	1	-1	1	-1	-1	0	0	0	0	1	1	-1	0	-1	-1	-1	-1	0	-1	1	0	1	0	0	1	1	-1	1	1	-1	-1	0	1	1	-1	0	-1	0	CNA-high
73	0	0	1	0	-1	-1	1	1	0	-1	-1	0	1	1	-1	0	-1	-1	0	0	-1	0	-1	0	-1	0	0	0	0	-1	0	0	0	-1	-1	1	1	-1	1	0	0	CNA-low
74	-1	0	0	0	-1	1	0	0	1	-1	0	0	-1	-1	-1	0	-1	-1	-1	-1	-1	-1	0	0	0	1	-1	-1	-1	-1	-1	1	1	1	1	1	-1	-1	1	0	0	CNA-high
75	-1	1	1	0	-1	1	-1	-1	1	-1	-1	-1	1	1	0	0	-1	1	-1	-1	0	0	1	1	-1	-1	1	0	0	-1	1	0	0	1	1	1	1	-1	1	0	0	CNA-high
76	0	0	1	0	-1	1	0	0	1	-1	1	1	-1	0	-1	1	-1	-1	-1	0	0	0	1	-1	-1	0	-1	0	0	-1	1	0	0	0	0	1	1	-1	1	1	0	CNA-high
77	0	0	0	0	-1	1	0	0	1	0	0	0	1	1	0	1	1	1	-1	-1	0	0	1	0	-1	-1	0	0	0	0	0	0	0	1	1	1	0	0	1	0	0	CNA-low
78	0	1	1	0	-1	1	0	0	1	1	1	0	1	0	-1	0	0	0	-1	0	1	1	-1	-1	0	1	0	1	0	0	1	0	0	-1	1	1	1	0	-1	-1	0	CNA-high
79	-1	1	0	0	-1	1	-1	-1	1	-1	0	0	0	0	-1	1	0	0	-1	-1	-1	-1	1	0	-1	-1	-1	-1	-1	-1	1	0	0	1	0	1	1	1	1	0	0	CNA-high
80	0	1	1	0	0	1	0	0	0	0	0	0	1	1	0	1	0	1	0	1	0	0	1	1	0	0	1	0	0	1	0	1	0	0	1	1	1	0	1	0	1	CNA-low

(Continued)

81	-1	1	1	0	-1	1	-1	-1	1	-1	1	-1	0	1	-1	1	0	0	0	0	-1	1	1	0	-1	-1	1	0	0	-1	1	0	0	0	0	1	1	-1	1	0	0	CNA-high
82	0	0	1	1	-1	1	-1	-1	1	0	1	1	-1	0	0	0	0	0	1	1	1	1	1	0	0	1	0	-1	-1	0	1	0	-1	1	0	1	-1	0	0	-1	0	CNA-high
83	0	0	1	0	-1	1	0	0	0	0	0	0	0	0	1	0	-1	1	0	0	0	0	0	0	-1	0	0	0	0	-1	0	1	0	0	1	0	0	-1	0	1	-1	CNA-low
84	0	1	0	0	-1	1	0	0	1	-1	0	0	-1	0	-1	1	-1	0	0	0	0	0	1	0	-1	1	-1	0	0	-1	0	0	-1	0	0	-1	1	0	-1	0	0	CNA-low
85	0	1	0	0	-1	1	0	0	1	-1	0	0	1	0	-1	1	-1	0	0	0	-1	-1	0	0	-1	-1	0	0	0	-1	0	0	0	-1	-1	1	1	0	1	0	1	CNA-low
86	0	0	0	0	-1	1	-1	-1	1	-1	0	0	1	1	-1	1	-1	-1	1	-1	0	1	1	0	-1	1	1	0	-1	0	0	1	1	-1	-1	-1	1	-1	1	0	0	CNA-high
87	-1	1	1	-1	-1	1	-1	-1	1	-1	1	1	1	-1	-1	1	-1	1	-1	-1	-1	0	1	1	-1	-1	1	1	1	-1	1	1	1	1	1	-1	1	1	1	0	0	CNA-high
88	0	0	0	0	0	0	-1	-1	1	-1	0	0	0	0	1	1	-1	-1	0	0	0	0	1	1	-1	1	0	0	0	1	1	0	0	1	1	0	0	1	0	0	0	CNA-low
89	-1	1	1	0	-1	0	0	0	1	0	-1	-1	1	1	-1	1	-1	0	0	-1	0	0	1	0	-1	1	1	1	1	-1	1	-1	-1	0	-1	1	1	-1	1	1	0	CNA-high
90	0	0	0	0	-1	1	0	0	1	-1	-1	0	0	0	-1	0	0	-1	0	0	-1	0	0	0	-1	0	0	-1	0	-1	0	0	0	0	0	-1	0	0	0	-1	-1	CNA-low
91	-1	0	1	1	-1	0	0	0	1	-1	0	0	1	1	-1	0	0	0	1	-1	0	-1	0	0	-1	-1	1	0	0	-1	1	1	1	1	-1	1	1	1	1	0	0	CNA-high
92	0	0	1	0	0	0	-1	-1	0	0	1	1	0	0	0	0	0	0	0	0	0	0	0	0	-1	-1	0	0	0	0	0	0	0	0	0	0	0	0	-1	0	0	CNA-low
93	0	0	1	0	-1	1	-1	-1	1	-1	1	1	1	1	-1	0	0	0	0	0	0	0	0	0	-1	0	1	0	0	-1	0	0	0	-1	0	1	1	0	1	0	0	CNA-low
94	-1	0	0	0	-1	1	0	0	1	-1	-1	0	0	0	-1	1	0	0	-1	-1	0	0	0	0	0	0	0	0	0	-1	0	0	0	0	0	1	0	0	0	0	0	CNA-low
95	0	1	0	1	-1	1	-1	-1	1	-1	1	1	0	0	1	1	1	0	0	-1	-1	-1	1	0	0	-1	-1	1	1	-1	-1	0	0	-1	0	1	1	1	-1	1	-1	CNA-high
96	1	1	1	1	0	1	-1	-1	0	0	0	0	0	0	0	1	0	0	0	0	0	0	1	0	-1	-1	-1	-1	-1	-1	1	1	1	-1	1	0	0	0	-1	0	0	CNA-low
97	0	0	1	1	-1	1	0	0	1	0	1	0	1	0	1	1	-1	0	1	1	-1	1	1	0	0	-1	0	0	0	-1	0	1	0	-1	1	1	1	0	1	1	1	CNA-high
98	0	0	0	0	-1	0	0	0	1	-1	0	0	0	0	-1	0	-1	-1	0	0	0	0	0	0	-1	-1	0	-1	0	-1	0	-1	-1	-1	0	1	1	-1	0	-1	-1	CNA-low
99	-1	1	0	-1	-1	1	-1	-1	1	0	0	0	1	1	-1	1	-1	-1	0	0	-1	0	0	0	1	-1	0	-1	-1	1	1	1	1	0	-1	1	0	0	1	0	1	CNA-high
100	1	0	1	0	-1	1	-1	-1	1	-1	-1	0	0	-1	0	0	1	1	0	-1	1	1	1	0	-1	-1	0	0	-1	-1	0	1	1	0	0	1	1	0	-1	1	0	CNA-high
101	-1	1	0	0	-1	0	0	0	1	-1	-1	0	0	0	-1	0	1	-1	-1	-1	0	0	0	0	-1	1	0	-1	0	-1	-1	0	0	0	0	1	-1	-1	0	0	0	CNA-low
102	0	1	1	1	-1	1	-1	-1	1	-1	0	0	1	0	-1	0	0	1	-1	-1	0	0	1	1	0	-1	1	-1	-1	0	0	1	0	-1	-1	1	1	-1	1	0	0	CNA-high
103	0	1	1	0	0	1	0	0	1	-1	1	1	0	1	-1	1	0	-1	-1	-1	0	1	1	0	1	0	0	-1	-1	-1	-1	-1	-1	1	-1	-1	1	-1	1	1	-1	CNA-high
104	-1	0	1	1	-1	0	-1	-1	1	-1	0	1	0	0	0	1	0	0	1	1	0	0	1	0	-1	1	0	1	1	0	0	1	1	-1	0	1	1	0	1	0	0	CNA-high
105	0	0	0	0	-1	0	0	0	0	0	0	-1	0	1	-1	1	0	0	0	0	0	0	1	0	-1	0	0	0	0	-1	0	0	-1	0	0	0	0	0	-1	0	0	CNA-low
106	-1	1	1	0	-1	1	-1	-1	0	-1	1	0	0	0	1	-1	-1	0	-1	-1	1	0	1	0	-1	1	0	0	-1	1	1	1	0	-1	0	1	0	-1	1	-1	0	CNA-high
107	0	0	0	0	-1	1	0	0	1	-1	0	0	0	0	0	1	-1	-1	0	0	0	0	0	0	0	0	0	0	0	0	0	0	0	0	0	0	0	0	1	0	0	CNA-low
108	-1	-1	0	0	-1	1	0	0	1	-1	-1	1	0	0	0	1	-1	-1	0	0	-1	0	1	-1	0	0	-1	-1	-1	-1	-1	0	0	-1	-1	1	1	-1	1	0	0	CNA-high
109	0	0	0	0	0	0	0	0	1	0	0	0	0	0	0	0	0	0	0	0	0	0	0	0	0	0	0	0	0	0	0	0	0	0	0	0	0	0	0	0	0	CNA-low
110	-1	-1	1	0	-1	1	-1	-1	0	-1	-1	-1	1	0	-1	0	1	0	-1	1	0	1	1	1	-1	1	1	0	0	0	1	1	1	0	-1	0	0	-1	0	1	-1	CNA-high
111	0	0	1	1	-1	1	-1	-1	1	-1	-1	-1	0	0	0	0	0	0	-1	-1	0	0	1	1	-1	0	0	-1	-1	0	0	0	0	-1	-1	1	1	-1	0	-1	1	CNA-high
112	-1	1	1	-1	1	1	-1	-1	1	0	-1	-1	1	1	-1	0	0	1	-1	-1	-1	-1	1	-1	1	0	0	0	0	-1	1	0	0	1	-1	1	1	-1	1	0	0	CNA-high
113	0	0	0	0	-1	1	0	0	0	-1	0	0	1	0	-1	0	-1	0	0	0	0	0	1	0	-1	0	0	0	0	-1	-1	-1	-1	-1	-1	0	0	0	1	0	0	CNA-low
114	-1	0	0	0	-1	1	0	0	1	-1	-1	-1	0	0	-1	0	1	1	0	-1	1	0	0	0	-1	1	0	-1	-1	0	0	0	-1	0	0	1	0	-1	1	-1	0	CNA-low
115	1	1	0	-1	-1	0	-1	-1	1	-1	1	1	-1	0	1	1	0	0	-1	-1	-1	-1	1	0	-1	0	0	-1	-1	0	0	0	0	0	1	0	0	0	0	-1	-1	CNA-high
116	0	0	0	0	-1	1	0	0	1	-1	0	-1	0	0	-1	0	-1	0	0	0	0	0	0	0	0	0	0	0	0	-1	0	0	0	0	0	0	0	0	0	1	0	CNA-low
117	0	-1	1	-1	0	1	0	0	1	-1	1	1	-1	1	-1	0	0	-1	1	0	-1	1	0	0	0	-1	-1	-1	1	-1	1	1	-1	1	-1	1	1	-1	0	0	0	CNA-high
118	1	1	1	1	-1	1	0	0	1	0	0	0	1	0	-1	1	-1	1	-1	-1	0	0	0	0	-1	-1	1	0	-1	-1	1	1	-1	0	0	0	1	1	0	0	0	CNA-high
119	0	0	0	0	-1	1	0	0	1	-1	0	0	0	0	-1	0	-1	-1	0	0	0	0	0	0	-1	-1	0	0	0	-1	0	0	-1	0	0	0	0	-1	0	-1	-1	CNA-low
120	0	1	1	0	-1	1	0	0	1	-1	-1	0	-1	-1	-1	1	0	-1	1	-1	1	0	1	1	-1	-1	-1	-1	-1	-1	1	-1	0	0	0	1	1	0	1	0	0	CNA-high

(Continued)

121	0	0	1	0	0	0	-1	-1	1	-1	0	0	1	1	1	0	0	1	-1	0	0	0	1	1	-1	-1	1	0	0	1	1	1	-1	1	1	1	1	-1	1	0	0	CNA-high
122	1	1	1	1	-1	1	-1	-1	1	-1	0	0	1	0	-1	1	-1	-1	-1	-1	0	0	1	1	-1	-1	0	-1	-1	1	-1	-1	-1	-1	0	1	1	-1	0	0	0	CNA-high
123	0	0	1	0	-1	1	0	0	1	0	0	-1	1	1	-1	1	-1	0	1	-1	1	-1	0	0	-1	0	0	0	1	-1	0	-1	0	-1	1	1	0	0	-1	0	0	CNA-low
124	0	0	0	-1	0	0	-1	-1	1	-1	1	-1	0	1	-1	1	1	-1	0	0	-1	1	0	-1	-1	1	-1	0	0	0	1	-1	0	0	0	1	1	0	0	0	0	CNA-low
125	0	1	0	0	-1	1	0	0	1	-1	0	0	1	0	-1	0	-1	-1	0	0	-1	-1	-1	-1	0	-1	0	0	0	-1	-1	0	0	-1	1	1	0	-1	1	0	0	CNA-low
126	-1	1	1	1	-1	1	-1	-1	1	0	-1	-1	1	1	-1	0	0	0	-1	-1	-1	-1	1	0	0	0	-1	1	1	-1	1	1	0	1	1	1	0	-1	0	-1	1	CNA-high
127	1	0	0	0	-1	1	0	0	1	-1	-1	1	1	1	-1	1	-1	0	-1	-1	-1	1	1	1	-1	-1	1	-1	-1	1	-1	-1	1	1	-1	1	-1	1	0	0	0	CNA-high
128	1	1	0	0	-1	1	-1	-1	1	-1	0	0	0	0	0	0	-1	-1	0	0	-1	1	0	0	-1	0	0	0	0	-1	0	0	0	-1	1	0	0	0	0	0	0	CNA-low
129	-1	1	1	0	-1	1	-1	-1	1	-1	0	1	0	0	-1	1	-1	-1	1	-1	1	1	1	1	1	-1	1	0	0	1	0	-1	-1	-1	0	1	1	-1	1	0	0	CNA-high
130	0	0	1	-1	-1	0	-1	-1	0	0	0	-1	0	0	-1	0	-1	-1	0	-1	0	0	1	0	0	0	0	0	0	-1	0	-1	1	0	0	0	0	0	0	0	0	CNA-low
131	0	0	0	0	-1	0	0	0	1	-1	0	0	0	0	-1	0	-1	-1	0	0	0	0	1	1	-1	-1	0	-1	0	-1	0	0	0	0	0	0	0	-1	0	-1	0	CNA-low
132	-1	0	0	0	-1	0	-1	-1	0	-1	0	0	0	0	0	0	-1	0	0	-1	0	0	0	0	-1	1	0	0	-1	-1	0	0	-1	-1	-1	0	0	0	1	-1	-1	CNA-low
133	0	0	0	0	-1	1	0	0	0	-1	0	1	-1	0	-1	0	-1	0	0	-1	0	0	0	0	-1	-1	1	-1	-1	0	0	0	0	-1	-1	0	1	-1	0	0	0	CNA-low
134	0	0	0	0	-1	0	0	0	1	-1	0	0	0	-1	-1	0	0	0	0	0	0	0	0	0	0	0	0	0	0	-1	0	0	0	-1	1	0	0	-1	1	-1	-1	CNA-low
135	0	0	1	0	-1	1	-1	-1	1	0	0	0	1	0	-1	1	0	-1	1	1	0	0	1	1	1	0	0	-1	-1	1	1	0	0	0	0	1	1	0	0	0	0	CNA-low
136	-1	1	0	0	-1	0	-1	-1	1	-1	-1	0	1	1	-1	1	0	1	0	0	0	0	1	1	0	0	-1	0	0	-1	1	1	1	0	-1	0	0	0	1	0	0	CNA-low
137	0	0	0	1	-1	0	-1	-1	1	-1	1	1	1	1	-1	1	-1	-1	-1	1	-1	-1	1	-1	-1	1	-1	-1	0	-1	-1	1	-1	0	1	1	1	1	1	0	0	CNA-high
138	0	0	0	0	0	0	0	0	0	0	-1	0	0	0	0	1	0	0	0	0	0	0	0	-1	-1	0	0	0	0	-1	-1	-1	-1	0	0	0	0	0	1	0	0	CNA-low
139	-1	1	1	0	-1	1	-1	-1	1	-1	0	0	1	1	0	1	-1	1	-1	-1	-1	-1	1	1	0	0	1	0	0	-1	1	0	0	0	0	0	1	0	1	0	0	CNA-high
140	0	1	0	-1	-1	1	-1	-1	1	-1	0	0	1	-1	0	1	1	-1	-1	-1	0	0	0	0	0	1	1	-1	-1	-1	1	-1	-1	-1	1	1	1	0	0	0	-1	CNA-high
141	0	0	0	0	-1	1	-1	-1	1	-1	0	0	0	0	-1	1	-1	-1	-1	-1	-1	-1	1	0	-1	0	0	0	0	-1	-1	0	-1	-1	1	0	0	0	0	0	0	CNA-low
142	0	0	1	0	-1	1	0	0	1	-1	1	0	1	-1	-1	1	0	-1	-1	-1	1	1	-1	-1	-1	1	0	1	-1	-1	1	-1	-1	0	0	0	0	1	-1	0	0	CNA-high
143	0	0	0	0	-1	1	0	0	1	-1	0	0	0	0	-1	1	-1	-1	-1	-1	-1	0	0	0	0	-1	0	-1	0	-1	0	0	-1	0	0	0	0	0	1	-1	-1	CNA-low
144	0	0	0	0	-1	1	0	0	1	-1	0	0	-1	1	-1	1	-1	-1	1	-1	0	0	1	1	-1	1	0	-1	-1	0	0	1	1	0	-1	1	1	1	1	0	0	CNA-high
145	0	0	0	0	-1	1	-1	-1	0	0	0	0	0	-1	-1	0	-1	1	-1	-1	0	1	0	-1	-1	0	0	0	0	0	0	-1	-1	-1	-1	0	1	0	0	-1	-1	CNA-low
146	-1	1	0	1	-1	1	0	0	1	-1	0	0	0	0	-1	1	-1	0	0	0	0	0	0	0	0	0	-1	0	0	-1	0	0	0	-1	1	0	1	-1	1	0	0	CNA-low
147	-1	1	1	0	-1	1	-1	-1	1	-1	0	0	1	1	-1	1	-1	0	1	0	-1	-1	1	1	-1	0	0	0	0	-1	0	0	-1	1	-1	0	1	-1	0	0	0	CNA-high
148	-1	-1	1	0	-1	1	-1	-1	1	-1	-1	-1	1	1	1	1	1	1	1	-1	-1	-1	-1	1	-1	1	0	1	-1	-1	-1	1	1	1	1	0	0	-1	0	0	0	CNA-high
149	0	0	0	0	-1	0	-1	-1	1	-1	0	0	0	0	-1	0	-1	-1	0	0	-1	0	0	1	-1	0	0	-1	0	-1	0	0	0	0	0	0	0	-1	0	-1	-1	CNA-low
150	0	1	1	0	-1	0	0	0	1	-1	1	0	1	1	0	0	0	0	-1	-1	-1	0	1	0	0	-1	-1	-1	-1	1	1	1	0	0	-1	1	-1	-1	1	1	1	CNA-high
151	0	1	0	0	-1	0	-1	-1	1	0	0	0	0	-1	-1	0	-1	-1	0	0	0	0	1	0	0	-1	0	0	0	0	0	0	-1	0	0	0	0	-1	0	0	0	CNA-low
152	-1	1	0	0	-1	1	-1	-1	0	0	-1	0	1	1	0	0	0	0	0	-1	0	0	1	0	-1	-1	1	0	0	0	0	1	-1	0	0	1	1	-1	-1	0	0	CNA-low
153	0	1	1	0	-1	1	-1	-1	0	-1	1	-1	1	1	-1	1	0	0	-1	-1	0	0	1	1	0	-1	1	-1	0	-1	0	0	0	0	0	1	1	0	1	0	0	CNA-high
154	0	1	1	1	-1	1	0	0	1	-1	-1	-1	-1	1	-1	0	0	0	-1	-1	0	0	1	1	-1	-1	1	-1	0	-1	1	1	1	-1	-1	1	1	-1	1	0	0	CNA-high
155	0	0	0	0	-1	0	0	0	1	-1	0	0	0	0	0	0	0	0	-1	0	0	0	0	0	-1	0	0	0	-1	0	0	1	-1	0	0	0	-1	-1	0	-1	-1	CNA-low
156	-1	1	1	0	-1	1	0	0	1	-1	1	-1	-1	1	0	0	1	1	0	-1	0	-1	0	0	0	-1	-1	0	1	0	1	0	-1	-1	-1	0	0	0	-1	1	-1	CNA-high
157	0	0	0	0	-1	1	-1	-1	0	-1	0	0	1	0	-1	0	-1	0	-1	0	0	0	0	0	-1	0	0	0	0	0	-1	0	-1	-1	1	0	0	0	-1	-1	-1	CNA-low
158	-1	1	0	-1	-1	1	-1	-1	1	-1	-1	0	1	0	0	0	1	0	-1	-1	1	0	1	0	-1	0	-1	-1	-1	0	0	1	0	1	1	1	1	-1	0	0	0	CNA-high
159	0	0	0	0	-1	1	-1	-1	0	0	-1	0	1	0	0	0	-1	0	0	0	0	0	0	0	0	0	-1	0	-1	-1	-1	0	0	0	0	-1	0	0	-1	0	0	CNA-low
160	0	0	0	0	-1	1	-1	-1	1	-1	0	0	0	0	0	0	-1	-1	-1	-1	-1	0	0	0	-1	0	0	-1	-1	0	0	0	0	0	0	0	0	0	0	-1	-1	CNA-low

(Continued)

161	0	0	0	0	0	1	0	0	1	-1	0	0	0	0	-1	1	-1	-1	-1	0	-1	0	0	0	0	1	1	1	1	1	0	0	-1	0	1	1	1	0	1	-1	0	CNA-low
162	-1	0	1	0	-1	1	-1	-1	0	-1	0	0	0	0	-1	0	-1	-1	-1	-1	0	0	0	0	-1	-1	0	0	0	-1	-1	1	0	0	0	0	0	-1	0	0	0	CNA-low
163	0	1	0	0	-1	1	-1	-1	1	-1	1	1	0	-1	-1	1	0	-1	1	0	0	0	0	0	-1	-1	0	1	1	-1	0	0	0	0	1	1	1	-1	1	1	-1	CNA-high
164	-1	1	0	0	0	0	0	0	1	-1	0	0	1	1	0	1	-1	-1	0	0	0	0	0	1	-1	-1	0	0	0	0	1	-1	-1	0	0	0	0	-1	0	0	0	CNA-low
165	-1	1	1	1	-1	1	0	0	1	-1	0	0	1	-1	0	1	0	-1	-1	-1	1	1	1	1	0	-1	-1	0	1	0	0	-1	-1	-1	-1	1	1	-1	1	0	0	CNA-high
166	0	0	-1	-1	-1	1	0	0	0	0	0	0	0	0	0	0	0	-1	-1	-1	0	0	0	0	-1	-1	0	0	-1	-1	0	-1	-1	0	0	-1	-1	0	-1	1	0	CNA-low
167	-1	0	0	0	-1	1	-1	-1	1	-1	1	0	0	1	0	1	0	0	0	0	-1	0	1	0	-1	0	1	0	-1	-1	1	0	0	0	0	1	-1	1	1	0	0	CNA-low
168	0	0	0	0	-1	1	0	0	1	-1	0	0	0	0	0	0	-1	0	0	0	0	0	0	0	-1	-1	-1	0	-1	-1	0	1	-1	0	0	0	0	0	-1	0	0	CNA-low
169	0	0	0	0	-1	1	0	0	1	-1	-1	1	-1	1	0	1	0	1	1	-1	0	0	-1	0	-1	0	-1	-1	0	-1	0	1	-1	1	-1	1	1	-1	1	1	1	CNA-high
170	0	0	0	0	0	1	0	0	1	-1	1	0	0	0	-1	1	0	0	0	0	-1	0	1	0	0	-1	0	0	-1	-1	0	0	0	0	0	0	0	-1	1	0	0	CNA-low
171	-1	0	0	0	-1	1	-1	-1	1	-1	0	0	0	0	0	0	-1	-1	0	-1	-1	0	0	0	-1	-1	0	0	0	-1	0	-1	-1	0	0	0	0	0	0	0	0	CNA-low
172	0	0	1	0	-1	1	0	0	1	-1	1	0	1	1	-1	1	1	-1	-1	0	-1	-1	1	0	-1	-1	-1	0	0	1	0	-1	0	-1	0	1	1	-1	-1	0	0	CNA-high
173	1	1	0	0	-1	1	0	0	1	-1	1	1	0	0	-1	1	-1	-1	-1	-1	0	0	1	1	-1	-1	0	-1	-1	-1	1	0	0	1	1	1	1	-1	0	0	0	CNA-high
174	0	0	1	0	-1	1	-1	-1	1	-1	1	1	0	1	-1	0	0	0	0	0	-1	-1	0	0	0	0	1	1	1	-1	0	1	-1	-1	1	0	1	-1	1	0	0	CNA-high
175	-1	1	1	0	-1	1	-1	-1	1	-1	-1	-1	-1	1	-1	1	1	1	-1	-1	1	-1	1	0	-1	-1	1	-1	1	1	1	1	1	1	0	1	1	1	1	0	0	CNA-high
176	-1	0	0	1	0	1	-1	-1	1	-1	1	1	0	0	0	1	-1	-1	-1	0	0	0	1	0	-1	-1	-1	1	1	-1	0	-1	-1	-1	-1	1	1	-1	0	0	0	CNA-high
177	0	0	0	-1	-1	1	0	0	1	-1	0	0	0	0	0	0	-1	-1	0	0	-1	0	0	0	-1	-1	0	0	0	-1	0	0	0	0	0	0	-1	0	0	-1	-1	CNA-low
178	0	0	1	0	-1	1	-1	-1	1	0	0	0	0	0	-1	1	-1	-1	1	0	0	0	1	1	0	0	0	1	1	0	1	0	0	0	1	1	1	-1	1	1	1	CNA-high
179	0	1	0	0	-1	1	0	0	1	-1	0	0	0	0	0	0	-1	-1	-1	-1	-1	0	0	0	-1	0	1	-1	0	0	0	0	0	-1	1	0	1	-1	0	0	0	CNA-low
180	-1	1	0	0	-1	1	-1	-1	0	-1	1	1	1	-1	-1	1	1	1	-1	-1	-1	0	-1	0	-1	1	0	-1	-1	1	1	1	-1	1	-1	1	1	0	0	0	0	CNA-high
181	0	0	0	0	-1	1	0	0	-1	-1	0	0	0	1	0	0	0	0	0	0	0	0	0	0	0	1	0	-1	-1	0	0	0	0	0	-1	0	0	-1	0	0	0	CNA-low
182	0	1	1	0	-1	1	0	0	1	-1	0	0	0	1	-1	1	-1	-1	0	0	0	0	0	0	-1	0	0	-1	-1	0	0	0	-1	-1	0	-1	0	0	1	0	0	CNA-low
183	0	0	0	0	-1	1	0	0	1	0	0	0	0	0	-1	1	0	-1	-1	-1	0	0	0	0	0	0	0	0	0	-1	0	-1	-1	0	0	0	0	0	1	0	0	CNA-low
184	0	1	0	0	-1	1	-1	-1	1	-1	-1	0	1	1	0	1	1	-1	1	1	-1	1	1	1	0	0	-1	-1	-1	-1	-1	-1	-1	-1	1	1	1	-1	0	0	0	CNA-high
185	-1	1	1	0	-1	1	-1	-1	1	1	0	0	0	0	0	0	-1	0	0	-1	-1	0	0	1	-1	-1	-1	1	1	-1	1	-1	-1	-1	1	1	1	0	-1	0	0	CNA-high
186	-1	1	1	1	0	0	-1	-1	1	-1	1	-1	1	0	1	1	-1	1	-1	-1	0	1	1	0	-1	0	-1	1	1	1	1	0	0	0	0	0	0	-1	1	0	-1	CNA-high
187	-1	1	0	0	-1	0	-1	-1	1	-1	0	0	1	1	-1	1	0	-1	-1	0	0	0	0	0	-1	0	0	0	0	-1	1	1	1	0	0	0	0	-1	1	0	0	CNA-low
188	0	0	1	0	0	1	-1	-1	0	-1	-1	1	1	1	-1	0	0	1	-1	-1	-1	-1	0	0	-1	1	0	-1	-1	0	0	0	0	0	1	1	1	-1	1	0	0	CNA-high
189	-1	1	1	0	-1	1	0	0	1	0	1	0	0	1	-1	0	0	0	-1	-1	-1	0	1	1	0	0	1	0	0	-1	1	0	0	-1	-1	1	1	1	1	0	0	CNA-high
190	0	0	0	0	0	0	0	0	0	0	0	0	0	0	0	0	0	0	0	0	0	0	0	0	0	0	0	0	0	0	0	0	0	0	0	-1	1	0	0	0	0	CNA-low
191	0	0	1	0	-1	0	-1	-1	1	-1	1	0	1	0	0	1	0	0	-1	0	1	1	1	0	1	1	1	0	1	0	0	1	-1	0	0	0	1	-1	1	0	0	CNA-low
192	0	0	0	0	-1	1	-1	-1	1	0	0	0	0	0	-1	0	0	0	-1	0	-1	-1	0	0	-1	0	-1	-1	-1	-1	0	0	-1	0	0	1	0	0	-1	-1	-1	CNA-low
193	1	1	1	1	0	1	0	0	1	0	1	0	1	1	0	1	0	0	0	0	0	0	0	0	1	0	0	1	0	0	0	0	0	-1	1	1	1	1	0	1	1	CNA-low
194	1	1	0	-1	-1	1	-1	-1	1	-1	1	1	1	-1	1	1	-1	-1	0	-1	-1	0	0	0	0	1	1	-1	0	-1	0	0	1	0	0	1	1	1	1	-1	-1	CNA-high
195	0	0	1	0	-1	1	-1	-1	1	1	0	-1	-1	1	-1	1	-1	-1	1	-1	-1	1	1	1	-1	-1	0	1	-1	-1	1	0	0	-1	0	1	1	-1	1	-1	-1	CNA-high
196	-1	1	0	0	-1	1	0	0	1	-1	-1	-1	0	0	0	0	0	1	-1	-1	0	0	0	0	-1	-1	0	-1	0	0	0	0	0	-1	1	0	0	0	-1	-1	-1	CNA-low
197	-1	0	0	0	-1	0	-1	-1	1	-1	0	0	1	0	0	0	0	1	1	-1	0	0	0	0	0	0	-1	0	0	-1	0	1	0	0	-1	0	0	-1	0	0	0	CNA-low
198	0	0	0	0	-1	1	-1	-1	0	0	0	0	0	0	-1	0	0	0	-1	-1	0	0	0	0	-1	0	-1	0	0	-1	0	-1	-1	0	0	0	0	0	0	0	0	CNA-low
199	0	0	0	0	-1	1	0	0	1	-1	1	0	0	0	-1	0	-1	-1	0	0	0	0	0	0	-1	0	0	-1	-1	-1	0	0	0	-1	0	0	0	-1	1	0	0	CNA-low
200	0	0	0	0	0	0	0	0	0	0	0	0	0	0	0	0	-1	-1	0	-1	0	0	0	0	0	0	0	0	0	0	0	0	0	-1	0	0	0	0	0	0	0	CNA-low

(Continued)

201	1	1	0	-1	0	0	-1	-1	1	-1	0	0	1	-1	1	1	-1	-1	-1	-1	0	0	0	0	-1	0	0	1	1	-1	-1	0	0	1	1	1	1	1	1	0	0	CNA-high
202	0	1	0	0	-1	1	-1	-1	1	-1	-1	1	1	1	0	1	-1	0	0	1	1	0	1	0	0	-1	-1	-1	-1	-1	1	-1	1	1	0	0	1	-1	1	0	1	CNA-high
203	0	1	0	0	-1	1	0	0	1	-1	0	0	-1	-1	-1	1	-1	-1	-1	0	0	0	0	1	-1	-1	-1	0	0	-1	-1	-1	-1	0	0	1	1	-1	-1	0	0	CNA-high
204	-1	1	0	0	0	0	0	0	0	0	0	0	1	-1	-1	1	0	-1	1	0	-1	0	0	0	-1	0	-1	0	1	0	1	-1	-1	0	-1	0	0	-1	0	0	0	CNA-low
205	-1	0	0	1	-1	-1	0	0	1	-1	1	1	-1	0	-1	1	-1	-1	1	1	0	1	1	0	-1	1	0	0	1	-1	1	-1	-1	-1	1	1	1	0	1	0	0	CNA-high
206	0	0	1	0	-1	0	0	0	0	-1	0	0	0	0	-1	1	-1	0	0	0	0	0	0	0	0	0	0	0	0	-1	0	0	-1	0	0	0	0	0	0	-1	1	CNA-low
207	1	1	1	1	-1	1	0	0	1	-1	-1	-1	0	0	-1	1	-1	-1	0	0	0	1	1	1	0	1	1	0	0	-1	1	-1	-1	0	0	1	1	-1	1	0	0	CNA-high
208	-1	0	1	0	-1	0	1	1	1	-1	0	0	1	1	0	0	-1	-1	-1	-1	1	1	1	0	-1	1	-1	1	1	-1	0	-1	1	-1	1	1	1	-1	1	-1	1	CNA-high
209	0	0	1	-1	-1	1	-1	-1	1	-1	0	0	1	0	1	1	0	-1	1	-1	0	0	1	0	-1	1	-1	1	0	-1	1	1	-1	1	1	0	0	0	0	0	0	CNA-high
210	0	1	0	0	-1	1	0	0	1	-1	0	0	0	0	0	0	0	0	0	-1	0	0	0	0	0	-1	0	0	-1	-1	0	0	0	-1	0	0	0	-1	0	0	-1	CNA-low
211	0	0	0	0	-1	1	-1	-1	1	-1	0	0	-1	0	-1	0	-1	-1	0	0	-1	0	0	0	-1	-1	0	0	-1	-1	0	0	0	-1	0	0	0	-1	1	-1	-1	CNA-low
212	0	0	1	1	-1	0	0	0	1	-1	0	0	1	0	0	0	0	1	-1	-1	0	0	0	-1	-1	0	1	1	-1	0	1	1	0	0	0	1	0	0	0	-1	0	CNA-low
213	-1	1	1	-1	-1	1	-1	-1	1	-1	0	0	1	1	-1	0	0	0	-1	-1	0	0	0	0	0	0	0	-1	-1	0	0	1	1	0	0	0	-1	-1	0	-1	-1	CNA-high
214	-1	1	0	0	-1	1	-1	-1	0	0	1	-1	1	1	-1	1	-1	-1	-1	-1	0	0	0	0	-1	0	1	-1	-1	-1	0	0	0	1	1	1	1	0	0	0	0	CNA-high
215	-1	1	0	1	0	1	-1	-1	1	-1	0	1	0	1	-1	1	-1	-1	1	1	-1	-1	1	0	-1	-1	1	-1	1	1	0	1	-1	-1	0	0	1	-1	0	-1	0	CNA-high
216	0	0	1	1	0	0	1	1	0	-1	0	-1	1	1	-1	0	-1	0	0	-1	0	0	1	0	-1	0	1	0	0	1	1	1	-1	0	1	0	1	-1	1	-1	1	CNA-high
217	0	1	0	0	-1	0	-1	-1	1	0	0	-1	0	0	0	0	-1	0	-1	-1	-1	-1	0	0	0	0	-1	0	0	-1	-1	0	0	0	0	0	0	0	0	1	-1	CNA-low
218	0	0	0	0	-1	0	0	0	1	-1	0	0	0	0	-1	1	0	0	1	0	0	0	1	0	0	0	0	0	1	-1	0	-1	-1	0	0	1	1	-1	1	0	0	CNA-low
219	-1	0	1	0	-1	1	0	0	0	-1	0	0	0	0	0	0	0	0	0	0	-1	0	0	0	0	0	0	0	0	-1	0	0	0	0	-1	0	0	0	0	0	-1	CNA-low
220	0	0	1	0	-1	1	0	0	1	-1	0	-1	1	0	-1	-1	0	-1	0	0	0	0	0	0	-1	0	-1	0	0	-1	-1	0	0	0	0	0	0	-1	0	-1	0	CNA-low
221	0	0	1	0	-1	1	0	0	1	-1	-1	0	1	0	-1	1	-1	1	-1	0	1	0	1	1	0	1	0	0	1	0	0	1	0	0	0	1	1	-1	1	-1	1	CNA-high
222	0	0	1	0	-1	1	0	0	1	-1	0	0	0	0	0	1	-1	-1	-1	1	-1	0	0	0	0	-1	0	-1	-1	-1	-1	-1	-1	0	0	0	0	-1	1	0	0	CNA-low
223	1	1	1	1	-1	1	0	0	1	0	0	0	1	1	-1	1	0	0	0	0	1	-1	1	0	0	1	0	0	1	1	1	0	0	0	0	1	1	1	1	1	1	CNA-high
224	0	1	0	0	-1	0	0	0	1	-1	0	0	0	0	0	1	-1	-1	0	-1	0	0	0	0	0	0	0	0	0	-1	0	0	0	0	0	1	0	0	1	0	0	CNA-low
225	0	0	0	0	0	0	0	0	0	0	0	0	0	0	0	0	0	0	0	0	0	0	0	0	0	0	0	0	0	0	0	0	0	0	0	0	0	0	0	0	0	CNA-low
226	0	0	0	0	-1	1	0	0	1	1	-1	-1	1	0	0	0	-1	-1	0	0	-1	-1	-1	-1	-1	1	0	0	0	-1	-1	-1	-1	-1	0	1	1	0	0	-1	1	CNA-high
227	0	0	0	0	0	1	-1	-1	0	-1	0	-1	0	0	0	0	-1	0	0	-1	0	0	0	0	-1	0	0	0	0	-1	0	0	0	0	0	0	0	0	0	1	-1	CNA-low
228	0	0	0	0	-1	1	0	0	1	-1	0	0	1	0	-1	1	0	-1	-1	0	0	0	0	0	1	0	0	0	-1	-1	0	-1	-1	0	0	0	1	0	1	0	0	CNA-low
229	0	0	1	0	-1	1	0	0	1	-1	0	0	0	0	-1	1	-1	-1	1	-1	-1	0	0	0	-1	0	0	0	0	-1	-1	0	0	0	0	0	0	0	0	0	0	CNA-low
230	0	0	1	0	-1	1	0	0	1	-1	1	0	0	-1	-1	0	-1	-1	-1	-1	-1	-1	1	0	-1	1	0	-1	0	-1	1	-1	-1	-1	1	0	1	-1	1	0	1	CNA-high
231	0	0	0	0	-1	0	-1	-1	1	-1	0	0	0	1	0	0	-1	1	-1	-1	-1	0	-1	-1	-1	1	-1	0	-1	0	0	1	1	0	-1	0	-1	-1	1	1	-1	CNA-high
232	-1	0	0	0	-1	1	-1	-1	1	-1	0	0	0	0	1	1	-1	1	-1	-1	0	0	0	0	-1	-1	0	-1	-1	0	0	0	0	0	0	0	0	0	1	-1	0	CNA-low
233	0	0	0	0	1	1	0	0	1	-1	1	0	0	0	-1	1	0	0	1	0	0	0	0	0	0	0	0	0	0	0	0	0	0	0	1	0	1	0	0	0	0	CNA-low
234	0	0	0	0	0	0	0	0	0	0	1	0	0	0	-1	1	0	0	0	0	0	0	0	0	0	0	0	0	0	-1	0	-1	-1	-1	0	0	0	0	-1	0	0	CNA-low
235	0	1	1	-1	-1	1	-1	-1	1	-1	1	1	1	0	-1	1	-1	-1	0	0	0	0	1	1	-1	0	1	-1	-1	1	1	1	1	0	1	1	1	-1	-1	0	0	CNA-high
236	0	1	1	0	-1	0	-1	-1	1	-1	-1	1	1	1	0	1	-1	-1	0	-1	0	0	1	0	-1	0	-1	-1	-1	-1	1	1	-1	0	0	1	1	-1	-1	0	0	CNA-high
237	-1	1	0	0	-1	0	-1	-1	1	-1	-1	0	1	0	-1	0	-1	0	0	-1	1	-1	1	0	-1	0	0	0	0	-1	0	1	0	-1	-1	1	0	0	1	0	0	CNA-low
238	0	1	0	0	0	0	0	0	0	0	-1	0	1	0	0	1	1	1	0	0	0	0	1	1	0	-1	1	0	1	-1	1	1	1	0	1	0	0	1	1	1	1	CNA-low
239	1	-1	0	0	-1	1	-1	-1	1	-1	1	0	1	1	-1	1	0	-1	-1	1	0	1	1	-1	-1	1	-1	-1	-1	-1	0	1	1	-1	1	1	-1	-1	1	0	0	CNA-high
240	0	0	1	0	-1	1	0	0	1	0	0	0	1	-1	-1	1	-1	0	0	0	-1	0	1	0	0	0	-1	1	1	0	0	0	-1	0	0	1	1	-1	1	0	-1	CNA-low

(Continued)

241	-1	1	1	0	-1	0	-1	-1	1	-1	0	0	1	1	-1	1	-1	1	1	-1	1	0	1	0	1	0	0	0	-1	-1	1	0	1	1	-1	0	0	-1	1	0	0	CNA-high
242	0	0	0	0	-1	1	0	0	1	-1	0	-1	0	0	-1	1	0	0	-1	-1	0	0	0	0	-1	-1	0	-1	-1	-1	0	1	-1	0	1	0	0	1	0	0	0	CNA-low
243	0	0	0	0	0	1	-1	-1	1	-1	0	0	0	0	0	1	0	-1	0	-1	0	0	0	0	-1	0	0	-1	-1	0	0	0	0	0	0	0	0	0	0	0	0	CNA-low
244	-1	1	1	1	-1	1	-1	-1	1	-1	1	0	0	1	-1	-1	0	0	-1	-1	0	0	1	1	-1	-1	1	-1	1	1	1	0	-1	0	1	1	1	-1	0	0	0	CNA-high
245	-1	1	1	0	0	0	-1	-1	0	-1	0	0	0	0	-1	0	0	0	1	0	0	0	0	0	0	0	0	-1	0	-1	0	0	0	0	-1	0	0	0	0	0	0	CNA-low
246	-1	1	1	1	0	1	-1	-1	1	0	0	-1	1	1	-1	1	1	0	1	-1	0	0	0	0	0	0	0	0	-1	-1	1	-1	-1	0	1	1	0	0	1	0	1	CNA-high
247	0	1	1	1	0	1	0	0	1	0	1	0	1	1	0	0	0	1	0	0	0	0	1	0	0	1	0	0	0	0	1	1	1	1	1	0	0	0	1	1	1	CNA-low
248	-1	1	1	1	-1	0	-1	-1	1	-1	-1	1	1	1	-1	0	-1	1	-1	-1	-1	1	1	1	-1	-1	1	-1	-1	-1	1	1	1	1	1	1	-1	-1	1	-1	0	CNA-high
249	-1	1	1	-1	-1	1	0	0	1	-1	-1	-1	-1	1	-1	1	0	0	-1	-1	0	0	1	1	0	0	0	0	1	0	1	1	1	0	1	1	1	0	1	0	1	CNA-high
250	0	1	0	0	-1	1	0	0	1	-1	-1	-1	0	0	-1	1	-1	-1	-1	-1	-1	-1	0	0	0	-1	1	-1	-1	-1	1	-1	-1	-1	-1	1	1	-1	1	0	0	CNA-high
251	0	0	0	0	0	0	0	0	0	0	0	0	0	0	0	0	0	0	0	0	0	0	0	0	0	0	0	0	0	0	0	0	0	0	0	0	0	0	0	0	0	CNA-low
252	-1	0	1	1	-1	1	-1	-1	1	-1	1	-1	1	1	-1	1	-1	-1	-1	1	-1	1	1	0	-1	0	0	0	-1	-1	1	-1	0	0	0	-1	1	-1	1	-1	0	CNA-high
253	0	1	0	0	-1	-1	-1	-1	0	0	0	0	1	1	-1	-1	0	0	0	0	0	0	0	-1	0	-1	-1	0	-1	-1	0	-1	-1	-1	-1	1	1	-1	-1	0	0	CNA-low
254	-1	1	1	-1	-1	1	-1	-1	0	-1	1	1	1	1	1	1	-1	-1	-1	1	1	0	1	1	-1	-1	-1	-1	-1	1	1	1	1	1	1	1	1	-1	-1	0	0	CNA-high
255	0	1	1	0	-1	1	0	0	1	-1	0	1	0	0	-1	1	-1	-1	1	1	-1	0	1	-1	0	0	0	1	0	-1	1	1	0	0	1	0	1	-1	1	1	0	CNA-high
256	-1	1	1	0	-1	1	-1	-1	1	-1	-1	1	1	1	-1	1	0	1	-1	-1	1	1	1	0	-1	1	-1	-1	-1	0	1	1	-1	1	-1	1	-1	-1	1	0	1	CNA-high
257	0	1	0	0	-1	0	0	0	0	0	0	0	1	1	0	0	-1	1	0	0	0	0	0	0	0	-1	0	0	-1	-1	0	0	0	0	0	0	0	0	0	0	0	CNA-low
258	0	0	0	0	-1	1	0	0	1	-1	0	0	1	1	1	0	1	-1	0	-1	0	0	0	0	-1	1	0	-1	0	-1	-1	1	1	0	1	1	1	-1	0	0	0	CNA-low
259	-1	0	0	0	-1	1	0	0	1	-1	1	1	0	1	-1	0	0	0	-1	-1	0	0	1	0	0	-1	1	0	1	0	0	0	0	1	0	1	1	-1	1	0	1	CNA-low
260	0	1	1	0	-1	1	0	0	1	-1	0	0	1	0	-1	0	0	0	0	0	0	0	0	0	-1	-1	0	0	0	0	0	0	0	0	0	0	0	0	0	1	-1	CNA-low
261	0	0	0	0	-1	1	-1	-1	1	-1	0	0	0	0	0	0	-1	-1	0	0	-1	0	0	0	0	0	0	0	0	-1	-1	0	0	-1	0	1	1	-1	1	0	0	CNA-low
262	0	0	0	0	-1	1	0	0	0	-1	-1	-1	0	0	-1	1	0	0	-1	-1	-1	-1	-1	0	0	0	0	0	0	0	0	-1	-1	0	0	1	1	0	0	0	0	CNA-low
263	1	1	1	0	-1	0	-1	-1	1	0	-1	-1	0	0	-1	1	-1	-1	0	0	0	0	1	0	-1	0	-1	0	0	0	0	0	0	-1	0	1	1	0	-1	0	0	CNA-low
264	1	0	1	0	-1	1	0	0	1	-1	1	1	1	0	-1	1	0	-1	1	-1	0	1	1	-1	0	1	0	1	-1	-1	-1	1	1	-1	1	1	1	1	1	0	0	CNA-high
265	1	1	0	0	-1	1	-1	-1	1	0	-1	-1	1	-1	-1	1	0	0	-1	-1	-1	0	0	0	0	1	-1	0	0	-1	-1	0	-1	0	0	1	1	0	1	0	0	CNA-high
266	1	1	0	1	0	0	0	0	1	-1	0	-1	1	1	-1	-1	-1	-1	1	-1	0	0	-1	-1	-1	1	-1	0	0	0	0	1	1	0	1	1	1	0	1	-1	1	CNA-high
267	0	0	0	0	-1	1	0	0	1	0	0	-1	0	0	-1	0	-1	-1	0	0	0	0	0	0	-1	0	1	0	0	-1	0	0	-1	0	0	-1	0	-1	0	0	0	CNA-low
268	-1	1	1	0	-1	1	0	0	1	-1	0	0	0	0	-1	1	0	0	0	0	0	0	1	0	0	-1	-1	0	0	0	0	-1	-1	0	0	0	1	-1	0	0	0	CNA-low
269	0	1	1	1	-1	1	-1	-1	1	-1	1	-1	-1	1	-1	1	-1	-1	1	-1	-1	-1	1	-1	-1	1	1	0	-1	-1	0	0	0	0	0	-1	1	-1	1	1	-1	CNA-high
270	-1	-1	0	0	-1	1	-1	-1	1	-1	0	0	0	0	-1	1	0	-1	0	-1	0	0	0	0	-1	1	0	-1	-1	0	0	-1	-1	-1	-1	1	1	-1	1	0	0	CNA-high
271	0	0	0	0	-1	1	0	0	0	-1	0	0	0	0	-1	1	-1	0	-1	-1	-1	0	0	0	-1	-1	0	-1	-1	-1	0	-1	0	-1	0	0	0	0	0	-1	-1	CNA-low
272	0	0	0	0	-1	1	-1	-1	1	-1	1	1	0	0	-1	1	-1	-1	1	1	-1	1	1	1	-1	0	1	0	1	-1	0	1	-1	-1	1	1	1	0	-1	0	1	CNA-high
273	0	0	0	0	0	0	0	0	0	0	0	0	0	0	0	0	0	0	0	0	0	0	0	0	0	0	0	0	0	0	0	0	0	0	0	0	0	0	0	0	0	CNA-low
274	0	0	0	0	0	0	0	0	0	0	0	0	0	0	0	0	0	0	0	0	0	0	0	0	0	0	0	0	0	0	0	0	0	0	0	0	0	0	0	0	0	CNA-low
275	0	0	0	0	0	0	0	0	0	0	0	0	0	0	0	0	0	0	0	0	0	0	0	0	0	0	0	0	0	0	0	0	0	0	0	0	0	0	0	0	0	CNA-low
276	-1	1	1	1	-1	1	-1	-1	1	-1	-1	-1	1	1	0	0	0	1	1	-1	0	0	1	0	-1	0	0	-1	-1	0	1	1	1	1	1	1	1	-1	0	0	0	CNA-high
277	-1	1	1	0	0	1	-1	-1	1	-1	0	-1	0	1	-1	1	0	1	-1	-1	0	0	1	1	-1	1	1	-1	0	-1	1	1	-1	1	0	1	1	0	0	0	0	CNA-high
278	-1	1	1	1	-1	1	-1	-1	1	-1	1	1	1	1	-1	0	-1	0	-1	1	-1	-1	1	-1	1	0	-1	0	0	1	1	-1	1	-1	-1	1	1	0	-1	0	0	CNA-high
279	-1	1	0	1	-1	1	-1	-1	-1	-1	-1	0	0	0	-1	1	0	0	-1	0	1	0	0	0	-1	0	0	0	0	-1	0	-1	0	0	0	1	1	-1	0	-1	-1	CNA-low
280	-1	0	1	0	-1	1	-1	-1	0	0	-1	0	0	0	-1	0	-1	-1	0	-1	-1	-1	1	-1	-1	0	0	0	0	-1	0	0	0	0	0	0	0	-1	0	0	0	CNA-low

(Continued)

281	0	0	1	1	-1	1	0	0	1	-1	0	0	-1	1	0	1	-1	-1	-1	-1	-1	0	1	0	-1	0	0	0	0	1	-1	-1	-1	-1	0	1	1	0	-1	0	0	CNA-high
282	-1	0	1	0	-1	1	-1	-1	1	-1	-1	-1	1	1	1	1	-1	1	0	0	0	0	1	0	1	0	0	1	-1	-1	1	1	-1	1	-1	1	1	-1	-1	0	-1	CNA-high
283	-1	1	1	-1	-1	1	-1	-1	-1	-1	-1	0	1	1	-1	0	0	0	-1	-1	-1	0	0	0	1	-1	1	1	0	-1	1	0	0	-1	-1	1	1	-1	1	0	1	CNA-high
284	0	0	0	0	0	0	-1	-1	0	-1	0	0	0	0	0	0	0	0	-1	0	0	0	0	0	0	0	0	-1	-1	-1	-1	0	0	0	0	0	0	0	0	0	0	CNA-low
285	-1	1	0	0	-1	1	-1	-1	1	-1	-1	0	1	1	-1	1	0	-1	-1	-1	-1	-1	1	-1	1	1	-1	1	1	-1	0	1	0	-1	1	1	1	-1	1	0	0	CNA-high
286	-1	1	1	0	-1	1	-1	-1	1	-1	0	0	0	-1	-1	0	1	-1	-1	-1	-1	-1	1	0	-1	0	0	1	1	-1	1	0	0	0	1	1	1	0	1	-1	1	CNA-high
287	-1	1	1	0	-1	0	-1	-1	1	-1	0	-1	1	1	0	0	0	0	-1	-1	-1	0	1	0	-1	-1	0	0	-1	-1	1	1	-1	1	1	1	1	-1	1	0	0	CNA-high
288	0	0	0	0	-1	0	0	0	0	0	0	0	0	0	-1	0	0	0	-1	0	0	0	0	0	0	0	0	0	0	0	0	0	0	0	0	1	1	0	0	0	0	CNA-low
289	-1	0	1	0	-1	-1	0	0	1	-1	0	0	0	0	-1	-1	-1	1	-1	0	0	0	-1	0	0	-1	1	-1	-1	0	0	-1	-1	-1	-1	0	0	-1	0	-1	1	CNA-high
290	1	1	1	1	-1	1	-1	-1	1	-1	1	1	1	0	-1	-1	-1	-1	0	0	0	0	1	0	-1	0	0	0	0	0	0	0	-1	-1	-1	1	1	-1	1	0	0	CNA-high
291	0	0	0	0	-1	1	-1	-1	-1	-1	1	1	1	0	-1	0	0	0	0	0	0	0	0	0	0	0	0	0	0	-1	0	-1	-1	0	0	0	0	0	1	-1	1	CNA-low
292	0	1	1	1	-1	1	0	0	1	-1	0	0	1	1	-1	1	1	0	0	0	0	0	0	0	-1	1	0	0	0	1	1	1	-1	0	0	0	0	0	0	0	1	CNA-low
293	0	0	1	0	-1	1	1	1	1	-1	0	0	0	0	-1	0	1	-1	0	0	0	1	0	0	0	0	1	0	1	-1	1	0	0	-1	1	1	1	-1	1	0	-1	CNA-low
294	0	0	0	0	-1	1	-1	-1	1	-1	1	0	1	0	-1	1	-1	-1	0	0	0	0	1	0	-1	0	-1	0	0	-1	1	1	-1	0	0	1	1	-1	1	1	-1	CNA-high
295	0	0	0	0	-1	1	0	0	1	-1	0	0	0	0	-1	1	0	-1	0	0	-1	0	0	0	-1	0	0	0	-1	-1	0	0	0	0	0	0	1	-1	0	-1	-1	CNA-low
296	-1	1	-1	0	0	0	-1	-1	1	0	0	0	1	0	-1	0	0	1	-1	-1	-1	-1	1	0	0	-1	0	0	-1	-1	0	1	-1	1	1	-1	1	0	1	0	0	CNA-high
297	-1	1	0	0	-1	1	0	0	0	0	-1	0	0	0	0	-1	0	0	0	-1	0	0	0	0	0	-1	0	0	0	-1	1	0	0	-1	0	0	0	-1	0	0	0	CNA-low
298	0	0	1	1	-1	1	-1	-1	1	-1	-1	-1	-1	-1	1	1	-1	-1	1	-1	1	1	1	1	-1	0	1	1	1	-1	1	1	-1	-1	1	1	1	-1	-1	0	0	CNA-high
299	-1	0	0	0	-1	0	-1	-1	0	-1	-1	0	-1	1	0	0	-1	0	0	0	-1	0	1	0	-1	-1	0	0	-1	-1	0	0	-1	0	-1	0	0	0	0	-1	-1	CNA-low
300	0	0	0	0	0	0	0	0	0	0	0	0	1	-1	-1	1	-1	-1	0	0	0	0	-1	0	0	0	0	0	0	0	0	0	0	0	1	0	0	0	0	0	0	CNA-low
301	-1	1	0	0	-1	1	-1	-1	0	0	0	0	1	0	-1	1	-1	-1	1	0	0	0	1	0	-1	0	-1	-1	1	-1	0	-1	-1	-1	0	1	1	-1	-1	0	0	CNA-high
302	-1	1	1	0	-1	1	1	1	0	-1	-1	-1	0	1	-1	1	-1	1	-1	0	-1	-1	-1	1	-1	1	-1	-1	-1	1	0	1	1	1	1	1	1	-1	1	0	0	CNA-high
303	0	1	0	0	-1	1	-1	-1	0	-1	0	1	1	1	0	0	-1	1	0	0	-1	-1	1	1	1	-1	1	1	1	-1	1	1	1	0	1	0	0	0	-1	0	0	CNA-high
304	-1	0	1	1	-1	0	0	0	-1	-1	-1	1	0	1	-1	1	-1	1	1	-1	0	0	1	0	-1	-1	1	1	-1	-1	1	1	-1	0	-1	1	1	-1	1	0	1	CNA-high
305	-1	1	1	1	-1	1	-1	-1	1	1	-1	1	1	1	-1	-1	-1	1	-1	-1	-1	-1	1	-1	-1	-1	0	1	1	-1	1	-1	-1	-1	0	1	1	-1	1	0	0	CNA-high
306	1	1	1	1	-1	1	-1	-1	1	-1	-1	1	0	1	-1	0	-1	-1	-1	-1	-1	1	1	1	-1	-1	-1	-1	0	-1	0	-1	-1	-1	-1	1	1	-1	1	0	0	CNA-high
307	-1	1	0	0	-1	1	-1	-1	0	0	1	1	1	1	-1	-1	0	0	0	0	-1	-1	0	0	-1	-1	1	0	0	0	0	-1	-1	0	0	0	0	-1	-1	0	0	CNA-low
308	0	0	0	0	-1	0	-1	-1	1	-1	0	0	0	0	0	1	-1	0	0	0	-1	0	0	0	-1	0	-1	0	0	-1	-1	0	0	0	0	1	1	0	0	-1	-1	CNA-low
309	-1	1	1	1	-1	1	0	0	1	-1	1	-1	1	1	-1	1	-1	-1	1	1	-1	-1	1	1	-1	1	-1	0	-1	-1	0	-1	-1	0	-1	-1	1	1	1	-1	0	CNA-high
310	0	1	1	0	0	1	0	0	1	0	-1	0	0	0	0	1	0	0	0	0	0	0	1	1	0	0	0	0	0	1	0	0	0	0	0	1	1	-1	0	1	-1	CNA-low
311	0	0	0	0	-1	1	-1	-1	0	-1	0	0	1	1	-1	-1	0	-1	1	-1	-1	0	0	0	-1	1	-1	0	0	-1	0	1	-1	-1	-1	0	0	1	1	-1	-1	CNA-high
312	1	0	0	0	-1	0	0	0	1	-1	0	0	0	0	-1	1	0	0	0	0	-1	-1	1	1	-1	-1	0	-1	-1	0	0	0	0	-1	-1	1	1	-1	-1	0	0	CNA-low
313	0	0	1	0	0	1	-1	-1	1	-1	1	0	-1	0	0	1	0	0	0	0	0	0	-1	1	-1	-1	-1	-1	0	-1	-1	0	-1	-1	-1	1	0	-1	0	0	-1	CNA-high
314	-1	-1	1	0	-1	0	-1	-1	-1	-1	-1	0	1	1	-1	0	1	1	1	1	0	0	0	0	-1	-1	-1	0	-1	-1	1	1	1	0	-1	1	-1	0	0	-1	1	CNA-high
315	0	0	1	0	-1	1	-1	-1	1	-1	1	1	1	0	-1	1	1	-1	-1	-1	1	1	1	1	-1	1	-1	1	1	-1	0	-1	-1	-1	0	-1	1	-1	-1	0	0	CNA-high
316	-1	1	1	0	-1	1	0	0	1	-1	1	-1	1	0	0	0	1	-1	1	-1	-1	0	0	0	0	0	0	-1	0	-1	0	0	-1	-1	0	0	-1	0	1	-1	1	CNA-high
317	-1	0	0	0	-1	0	0	0	0	0	0	0	-1	0	0	0	-1	1	-1	-1	0	0	1	0	-1	0	-1	0	-1	-1	0	-1	-1	0	0	0	0	0	-1	-1	-1	CNA-low
318	-1	1	1	0	-1	1	-1	-1	0	-1	0	0	1	1	-1	1	0	-1	0	1	0	0	0	0	-1	1	-1	0	0	-1	0	-1	-1	-1	-1	1	1	-1	1	0	0	CNA-high
319	-1	0	1	0	-1	1	-1	-1	1	0	-1	-1	0	0	-1	1	-1	-1	1	1	-1	1	1	1	-1	1	-1	1	-1	-1	1	1	-1	-1	1	1	1	-1	-1	0	0	CNA-high
320	-1	1	1	-1	-1	1	-1	-1	-1	-1	1	-1	1	1	-1	0	1	1	-1	-1	-1	1	1	1	0	1	1	1	-1	-1	1	-1	-1	1	-1	0	1	1	0	0	0	CNA-high

(Continued)

321	0	0	0	0	0	0	0	0	1	-1	1	0	-1	0	-1	1	0	-1	1	-1	-1	0	0	0	-1	0	0	-1	-1	0	0	0	0	0	0	0	0	1	0	0	0	CNA-low
322	0	1	1	0	-1	0	-1	-1	1	-1	-1	1	1	1	-1	1	-1	1	1	-1	-1	-1	1	0	-1	0	-1	1	-1	-1	1	0	0	0	0	1	1	-1	1	0	0	CNA-high
323	0	0	0	0	-1	0	-1	-1	1	-1	0	0	0	0	0	0	-1	-1	-1	0	-1	-1	0	0	-1	-1	0	-1	-1	-1	-1	-1	0	-1	1	0	0	-1	-1	-1	-1	CNA-high
324	0	0	0	0	-1	1	-1	-1	1	-1	0	0	0	0	1	1	0	-1	1	1	0	0	0	0	-1	1	-1	1	1	-1	-1	1	1	-1	1	1	1	-1	-1	0	0	CNA-high
325	0	0	0	0	-1	1	-1	-1	0	-1	0	-1	0	0	0	0	-1	0	-1	-1	-1	0	0	0	-1	0	0	0	0	-1	0	-1	-1	-1	-1	-1	1	-1	-1	0	-1	CNA-low
326	0	1	1	0	0	1	1	1	0	0	-1	0	1	0	-1	1	0	1	-1	0	1	-1	1	0	0	0	-1	-1	-1	-1	1	1	0	-1	1	1	1	0	1	0	0	CNA-high
327	0	0	0	0	-1	0	0	0	0	0	0	0	1	1	-1	1	-1	-1	-1	0	-1	0	-1	1	-1	1	-1	0	0	-1	0	-1	-1	-1	-1	1	0	-1	0	0	0	CNA-low
328	0	0	1	1	-1	1	-1	-1	1	-1	1	1	1	0	-1	1	-1	-1	1	-1	-1	-1	1	-1	0	1	-1	-1	-1	0	0	0	0	1	1	1	1	-1	1	0	0	CNA-high
329	0	1	1	0	-1	0	0	0	1	-1	0	0	1	1	-1	1	-1	-1	0	0	-1	0	0	0	-1	1	0	0	0	-1	0	0	-1	-1	1	1	1	0	1	0	1	CNA-low
330	0	0	0	0	-1	1	0	0	0	-1	0	0	0	0	-1	0	0	0	-1	-1	0	0	0	0	-1	-1	0	0	0	-1	0	-1	-1	0	0	0	0	0	0	-1	-1	CNA-low
331	1	1	1	1	-1	1	0	0	1	-1	0	0	1	1	1	0	-1	-1	-1	-1	0	0	-1	-1	0	-1	0	1	-1	-1	-1	-1	-1	1	1	1	1	0	1	0	0	CNA-high
332	0	0	0	0	0	1	-1	-1	1	-1	0	1	1	1	-1	1	0	0	1	1	-1	1	1	1	0	0	1	0	0	-1	0	1	-1	-1	1	1	1	0	1	-1	0	CNA-high
333	0	0	1	1	-1	1	0	0	1	-1	0	0	0	0	-1	1	-1	-1	0	0	0	0	0	0	0	0	0	0	0	0	0	1	0	0	0	0	0	0	1	0	0	CNA-low
334	0	0	0	0	0	0	-1	-1	0	0	0	0	1	0	1	1	-1	-1	1	-1	0	0	0	0	-1	1	0	0	0	-1	-1	0	0	1	1	1	1	1	1	0	0	CNA-low
335	1	1	1	-1	-1	1	0	0	1	-1	1	1	1	-1	1	1	-1	-1	-1	-1	-1	-1	1	1	0	-1	0	-1	-1	-1	0	-1	-1	1	1	-1	-1	1	1	0	0	CNA-high
336	0	0	1	0	-1	0	0	0	1	-1	1	0	1	0	-1	1	-1	-1	1	-1	-1	1	1	0	-1	1	1	1	-1	-1	0	1	0	-1	1	0	0	-1	1	-1	1	CNA-high
337	-1	1	1	1	-1	1	-1	-1	1	-1	-1	0	0	1	-1	1	0	1	-1	-1	-1	-1	1	1	-1	0	-1	0	0	-1	1	0	-1	0	0	1	1	1	0	0	0	CNA-high
338	-1	1	1	0	-1	1	-1	-1	1	-1	0	0	1	1	-1	1	1	1	-1	-1	-1	-1	1	0	-1	0	0	-1	-1	1	1	1	1	1	-1	1	1	-1	1	1	1	CNA-high
339	0	0	0	0	0	0	0	0	1	1	0	0	0	0	-1	0	0	0	0	0	0	0	0	0	-1	0	0	0	0	0	0	0	0	0	0	0	0	-1	-1	0	0	CNA-low
340	-1	1	1	-1	-1	1	-1	-1	0	0	-1	-1	1	0	1	1	-1	0	0	0	0	0	1	1	-1	0	0	0	0	0	0	-1	-1	-1	1	0	0	1	0	0	0	CNA-low
341	0	0	0	0	0	0	0	0	0	0	0	0	0	0	0	0	0	0	0	0	0	0	0	0	0	0	0	0	0	0	0	0	0	0	0	0	0	0	0	0	0	CNA-low
342	0	0	1	1	-1	1	0	0	1	0	0	0	0	0	0	0	-1	-1	1	0	1	1	1	0	-1	0	1	1	1	-1	-1	-1	-1	-1	1	0	0	0	1	0	0	CNA-low
343	-1	1	0	0	-1	1	0	0	0	-1	-1	0	1	1	0	0	0	1	0	-1	1	0	1	1	-1	1	0	0	0	-1	1	1	-1	1	0	0	1	-1	0	-1	1	CNA-high
344	0	0	0	0	-1	1	-1	-1	1	-1	0	1	1	0	-1	1	-1	-1	-1	0	1	0	1	0	-1	0	-1	0	0	-1	0	0	1	0	1	1	1	-1	0	0	0	CNA-high
345	0	0	0	0	-1	1	-1	-1	1	-1	0	0	1	0	0	1	-1	-1	1	-1	0	1	1	1	-1	-1	0	-1	0	-1	1	-1	0	1	1	0	1	-1	1	0	0	CNA-high
346	-1	1	1	0	-1	1	-1	-1	1	-1	-1	-1	1	1	-1	1	-1	-1	1	0	-1	0	1	0	-1	1	0	0	0	-1	0	-1	-1	1	-1	1	0	0	0	0	0	CNA-high
347	0	1	1	1	-1	1	-1	-1	1	0	0	0	1	1	-1	1	0	0	0	0	-1	0	1	1	-1	-1	0	1	1	1	1	0	0	0	0	1	1	1	1	-1	1	CNA-high
348	1	1	0	0	-1	1	-1	-1	0	0	0	0	1	-1	0	0	0	0	-1	-1	1	1	0	-1	0	0	0	0	0	-1	-1	0	0	0	0	1	1	0	1	0	0	CNA-low
349	-1	1	1	0	-1	1	-1	-1	1	-1	0	0	1	0	-1	0	1	1	-1	-1	-1	0	1	1	-1	-1	1	0	1	1	1	1	1	1	0	1	1	-1	1	-1	1	CNA-high
350	0	1	1	1	-1	1	-1	-1	1	-1	0	0	1	0	-1	1	-1	-1	0	0	0	0	1	0	0	-1	-1	-1	-1	-1	-1	-1	0	0	0	1	1	-1	1	0	0	CNA-high
351	0	1	1	0	-1	1	-1	-1	1	0	0	0	1	1	-1	1	-1	-1	-1	-1	0	-1	1	-1	-1	-1	1	0	0	-1	-1	-1	-1	-1	1	1	1	-1	1	-1	1	CNA-high
352	0	0	0	0	-1	1	0	0	0	0	0	1	-1	0	0	0	0	-1	1	-1	0	0	1	0	0	0	-1	0	0	-1	0	1	1	0	0	0	0	0	0	-1	-1	CNA-low
353	-1	1	1	0	-1	1	-1	-1	1	-1	-1	-1	1	1	-1	0	-1	1	-1	-1	0	-1	1	1	-1	-1	1	-1	-1	1	1	1	1	1	1	1	0	1	1	1	1	CNA-high
354	-1	1	1	1	-1	1	-1	-1	1	-1	0	0	0	1	-1	0	0	-1	0	0	-1	-1	0	0	0	-1	0	0	0	-1	-1	0	0	0	1	1	0	0	1	-1	0	CNA-low
355	-1	1	0	0	-1	1	0	0	1	-1	0	0	0	0	0	0	-1	-1	0	0	0	0	0	0	-1	0	0	0	0	-1	0	-1	-1	-1	1	1	1	-1	1	0	0	CNA-low
356	0	0	1	0	-1	1	-1	-1	1	-1	0	0	1	0	-1	0	0	-1	-1	-1	0	0	0	0	0	0	0	1	0	-1	1	1	-1	-1	0	1	1	-1	0	0	0	CNA-low
357	0	0	0	0	-1	1	-1	-1	0	-1	1	1	0	1	-1	1	0	0	-1	1	-1	1	0	0	-1	1	-1	1	-1	1	0	1	-1	-1	0	0	1	0	-1	0	0	CNA-high
358	-1	1	1	1	-1	0	0	0	1	-1	0	-1	1	-1	-1	1	0	0	0	0	-1	-1	1	-1	-1	0	-1	0	0	-1	0	0	0	0	0	0	0	0	-1	0	0	CNA-low
359	1	1	0	0	-1	1	-1	-1	1	-1	0	0	-1	-1	1	1	1	1	-1	-1	-1	-1	1	1	0	-1	1	-1	-1	-1	-1	0	1	1	1	1	1	0	1	1	1	CNA-high
360	0	0	0	0	-1	0	-1	-1	0	-1	0	0	0	0	0	0	-1	-1	0	0	0	0	0	0	-1	0	1	0	0	-1	0	0	-1	-1	-1	0	0	0	0	-1	-1	CNA-low

(Continued)

361	-1	1	1	0	-1	0	-1	-1	0	0	-1	0	0	0	-1	1	-1	0	1	-1	-1	0	1	0	1	1	-1	0	0	-1	0	1	-1	0	-1	1	-1	-1	0	0	0	CNA-high
362	-1	0	0	0	-1	1	0	0	0	-1	1	0	1	1	-1	1	-1	-1	0	0	0	0	1	1	1	1	1	1	1	-1	1	0	-1	-1	1	-1	1	1	1	0	0	CNA-high
363	-1	1	0	0	-1	1	-1	-1	1	-1	-1	-1	0	0	0	1	-1	0	0	0	0	0	0	0	0	-1	1	0	0	0	-1	1	-1	0	-1	-1	1	1	-1	1	-1	0	CNA-high
364	-1	1	1	1	-1	1	-1	-1	-1	-1	0	0	1	1	-1	1	-1	-1	-1	-1	-1	-1	1	1	-1	1	-1	-1	1	-1	1	-1	-1	1	1	1	1	-1	1	0	1	CNA-high
365	0	0	0	0	-1	1	0	0	1	-1	0	0	0	0	-1	0	-1	1	-1	-1	0	0	0	0	-1	-1	1	0	-1	-1	0	0	0	0	0	0	0	0	0	-1	0	CNA-low
366	0	0	1	0	-1	1	0	0	1	-1	0	0	1	1	-1	0	-1	1	-1	0	0	0	1	0	-1	0	1	0	-1	-1	1	1	1	-1	0	1	1	-1	1	-1	0	CNA-high
367	0	0	1	1	-1	1	-1	-1	1	1	0	0	1	0	-1	1	1	-1	1	-1	-1	-1	0	1	-1	-1	-1	0	0	-1	0	1	1	1	1	-1	-1	-1	0	0	-1	CNA-high
368	0	0	0	-1	-1	1	-1	-1	1	-1	0	0	1	0	-1	1	-1	0	0	0	0	0	0	0	-1	-1	0	0	-1	-1	0	0	0	0	0	0	0	0	0	-1	-1	CNA-low
369	-1	-1	1	0	0	0	-1	-1	1	-1	1	1	1	1	-1	1	1	-1	0	0	-1	1	0	0	0	-1	-1	0	-1	-1	-1	0	-1	-1	-1	-1	1	1	1	0	0	CNA-high
370	0	1	0	0	0	1	1	1	0	0	1	0	0	0	0	0	0	0	0	-1	0	0	0	0	0	0	0	0	0	0	0	0	0	0	0	1	1	-1	0	0	0	CNA-low
371	0	0	0	0	0	0	0	0	0	0	0	0	0	0	0	0	0	0	0	0	0	0	0	0	0	0	0	0	0	0	0	0	0	0	0	0	0	0	0	0	0	CNA-low
372	1	-1	1	0	-1	0	-1	-1	1	-1	1	1	1	1	-1	1	0	-1	-1	-1	1	1	1	1	-1	0	1	-1	-1	-1	-1	0	0	1	-1	1	1	-1	1	1	-1	CNA-high
373	0	0	0	0	0	0	-1	-1	1	-1	0	0	0	0	1	1	-1	-1	0	1	-1	1	0	0	-1	0	-1	-1	-1	-1	-1	-1	-1	0	0	1	1	0	1	0	0	CNA-low
374	0	1	0	0	-1	1	0	0	1	-1	0	0	0	0	-1	0	-1	-1	0	0	-1	0	0	0	-1	0	0	0	0	-1	0	0	0	0	0	0	0	-1	0	1	-1	CNA-low
375	-1	1	0	-1	-1	1	0	0	-1	-1	1	-1	1	1	1	1	0	1	-1	-1	1	0	-1	-1	-1	1	1	1	1	-1	1	1	1	-1	1	1	1	-1	1	0	0	CNA-high
376	1	1	1	0	-1	1	-1	-1	1	-1	0	0	1	1	-1	1	-1	-1	-1	-1	-1	-1	1	-1	-1	-1	-1	-1	-1	-1	0	1	0	-1	0	1	-1	-1	1	0	0	CNA-high
377	1	0	1	0	-1	1	-1	-1	1	-1	0	0	1	0	-1	1	-1	-1	1	1	-1	0	1	0	-1	1	1	-1	0	0	0	0	0	0	0	0	0	1	0	1	1	CNA-high
378	-1	1	0	1	-1	1	-1	-1	1	0	0	-1	1	0	-1	1	-1	-1	1	1	-1	0	1	1	-1	-1	-1	-1	1	-1	1	1	-1	0	-1	1	1	-1	1	1	-1	CNA-high
379	-1	1	0	0	-1	1	0	0	1	-1	0	0	1	1	-1	1	-1	0	-1	0	0	0	0	0	-1	-1	0	-1	-1	0	0	0	0	0	0	-1	-1	-1	1	0	0	CNA-low
380	0	1	0	0	-1	1	0	0	1	-1	0	0	0	0	-1	1	0	0	1	-1	1	0	1	1	-1	1	-1	1	1	0	1	0	0	-1	1	1	1	1	0	1	1	CNA-high
381	0	0	0	0	-1	1	-1	-1	1	-1	1	1	-1	0	-1	1	0	1	-1	-1	0	0	0	0	0	-1	0	-1	-1	-1	0	0	0	0	0	0	1	0	-1	-1	-1	CNA-low
382	-1	1	1	0	-1	1	-1	-1	1	0	-1	-1	-1	1	0	1	1	0	0	-1	-1	-1	1	0	-1	-1	0	-1	0	0	1	1	0	0	1	1	0	-1	1	0	1	CNA-high
383	0	1	0	1	-1	1	0	0	1	-1	0	1	1	0	-1	0	0	1	-1	-1	-1	1	0	0	-1	0	0	0	-1	-1	0	1	1	-1	0	1	1	-1	-1	0	0	CNA-high
384	0	1	1	-1	-1	1	-1	-1	0	-1	0	-1	1	1	-1	1	1	-1	1	-1	-1	1	0	0	-1	1	0	0	-1	-1	0	0	-1	0	1	1	1	0	0	0	0	CNA-high
385	0	0	0	0	-1	1	-1	-1	0	0	1	1	0	0	0	0	0	0	0	0	1	0	0	0	0	1	0	0	0	0	0	0	-1	0	0	0	0	-1	0	0	0	CNA-low
386	-1	0	0	0	-1	1	0	0	1	-1	1	0	-1	1	-1	0	-1	0	0	-1	1	0	0	0	0	0	0	0	0	-1	0	-1	-1	0	-1	-1	0	-1	0	1	0	CNA-low
387	-1	0	1	-1	-1	1	0	0	0	0	0	0	1	1	1	1	0	1	0	0	1	-1	1	1	-1	0	0	1	-1	-1	1	1	0	0	0	0	0	-1	0	0	0	CNA-low
388	0	0	0	0	-1	0	0	0	1	0	0	0	0	0	-1	0	-1	0	0	0	0	0	0	0	-1	0	0	0	0	-1	0	0	-1	0	0	-1	0	0	0	0	0	CNA-low
389	0	0	0	0	-1	0	0	0	1	-1	1	1	0	1	-1	1	-1	-1	0	0	0	0	1	0	0	-1	1	0	-1	0	0	1	1	-1	0	1	1	0	1	0	1	CNA-low
390	-1	1	1	1	-1	1	0	0	1	0	0	0	1	1	1	1	0	1	-1	-1	1	0	0	0	0	-1	1	1	-1	1	1	1	1	1	1	1	1	1	0	0	0	CNA-high
391	0	0	0	0	-1	1	-1	-1	1	-1	0	0	0	0	0	0	-1	-1	1	0	0	0	0	0	-1	0	0	0	-1	0	0	0	0	0	0	0	0	0	-1	0	0	CNA-low
392	0	0	0	0	-1	1	-1	-1	1	-1	0	-1	0	1	-1	0	-1	-1	-1	0	-1	0	0	0	-1	0	0	0	0	-1	-1	-1	-1	-1	0	1	1	-1	0	0	-1	CNA-high
393	-1	1	1	0	-1	1	0	0	0	-1	1	1	0	0	-1	0	-1	-1	0	1	-1	1	1	0	-1	0	0	-1	1	-1	1	1	-1	-1	-1	1	1	-1	1	0	1	CNA-high
394	1	1	0	0	-1	1	0	0	1	-1	-1	-1	1	1	-1	1	-1	-1	1	-1	-1	0	0	0	-1	1	0	0	-1	-1	-1	0	0	-1	1	1	1	-1	-1	-1	1	CNA-high
395	0	1	0	0	-1	0	-1	-1	1	-1	1	0	1	1	-1	0	-1	-1	0	-1	0	0	1	0	-1	0	0	0	0	-1	-1	-1	-1	0	0	0	1	-1	1	0	0	CNA-low
396	0	0	0	0	-1	1	-1	-1	1	-1	-1	1	0	0	0	0	-1	0	-1	0	0	1	0	0	-1	-1	0	-1	0	-1	-1	-1	1	-1	0	0	0	-1	0	-1	-1	CNA-low
397	-1	0	0	0	-1	0	0	0	0	0	0	1	0	-1	-1	0	0	-1	-1	-1	-1	-1	0	0	0	0	-1	0	0	-1	0	0	0	0	0	0	0	0	0	0	-1	CNA-low
398	0	0	1	0	-1	1	-1	-1	0	0	0	0	1	1	1	1	1	1	1	-1	1	0	-1	-1	1	0	0	0	0	-1	1	1	1	1	1	1	1	0	-1	-1	1	CNA-high
399	-1	1	0	0	-1	1	0	0	0	-1	-1	-1	1	-1	0	0	0	0	0	0	0	0	0	0	0	1	0	0	0	0	0	0	0	0	0	0	0	-1	1	-1	0	CNA-low
400	0	0	1	1	-1	1	1	1	-1	-1	1	0	1	1	1	1	-1	-1	0	0	-1	-1	0	-1	-1	0	0	0	0	0	0	0	0	-1	-1	1	1	0	1	0	0	CNA-high

(Continued)

401	0	0	0	0	-1	1	1	1	1	-1	0	0	0	0	1	1	0	0	0	0	0	1	1	1	-1	-1	1	-1	-1	-1	1	0	0	1	1	1	0	1	0	0	0	CNA-low
402	-1	1	1	1	-1	0	-1	-1	1	-1	0	-1	1	1	-1	1	-1	0	-1	0	0	0	0	0	0	1	0	0	0	-1	1	1	1	-1	1	1	-1	-1	1	0	0	CNA-high
403	-1	0	1	1	-1	0	-1	-1	1	-1	-1	-1	1	1	-1	1	-1	1	1	-1	-1	-1	1	1	0	0	-1	1	1	-1	1	1	-1	0	-1	1	1	-1	1	-1	0	CNA-high
404	-1	1	0	0	-1	1	0	0	1	-1	0	0	1	1	1	1	-1	0	0	0	-1	-1	0	0	0	0	1	0	1	-1	0	-1	-1	0	0	1	1	-1	1	0	0	CNA-low
405	-1	1	1	0	-1	0	-1	-1	1	-1	0	-1	0	0	-1	0	-1	0	0	-1	0	0	0	0	-1	1	0	0	-1	-1	0	-1	0	0	0	1	-1	-1	0	0	1	CNA-low
406	0	1	0	0	-1	0	1	1	0	-1	-1	0	0	1	-1	1	0	1	0	-1	-1	0	0	0	-1	0	-1	1	-1	-1	0	1	-1	0	-1	-1	-1	-1	0	-1	-1	CNA-high
407	0	0	1	0	0	1	0	0	0	0	0	0	-1	1	-1	1	-1	-1	0	0	0	0	0	0	-1	0	0	0	-1	-1	0	0	0	0	0	0	1	0	0	0	0	CNA-low
408	0	0	1	0	0	1	0	0	1	0	0	0	1	0	0	1	0	-1	-1	1	-1	0	1	0	-1	1	1	0	0	-1	0	-1	0	1	1	0	0	0	1	1	1	CNA-low
409	0	0	0	0	-1	1	0	0	0	0	0	0	1	-1	-1	1	-1	0	-1	0	0	1	0	0	0	0	0	0	0	-1	0	0	-1	0	0	0	0	-1	0	0	0	CNA-low
410	-1	1	1	1	0	0	0	0	1	0	0	0	1	0	-1	-1	1	0	-1	-1	0	0	1	1	-1	1	1	0	0	-1	1	1	-1	-1	1	-1	1	-1	-1	0	0	CNA-high
411	-1	0	1	0	-1	1	-1	-1	1	-1	1	0	1	1	-1	0	-1	1	1	-1	0	0	1	1	0	-1	1	-1	-1	-1	1	-1	-1	-1	-1	-1	-1	0	1	0	0	CNA-high
412	0	1	1	1	-1	1	-1	-1	1	-1	0	1	1	1	-1	1	-1	-1	0	1	-1	-1	1	0	-1	0	0	0	-1	0	0	0	1	0	1	1	1	-1	1	1	0	CNA-high
413	0	0	1	0	0	1	-1	-1	1	-1	0	-1	1	-1	-1	0	0	0	0	-1	0	0	0	0	-1	0	0	-1	-1	-1	0	0	0	0	0	0	0	0	1	0	0	CNA-low
414	0	0	0	0	-1	1	0	0	1	-1	0	0	0	0	-1	1	-1	-1	0	0	1	0	0	0	-1	0	0	0	0	0	0	0	0	0	0	0	0	-1	1	0	0	CNA-low
415	0	0	1	0	-1	1	0	0	1	0	0	0	1	0	0	1	-1	0	0	0	0	0	1	0	0	0	0	0	0	0	0	0	0	0	0	1	1	0	0	0	0	CNA-low
416	-1	1	1	1	-1	1	-1	-1	1	-1	-1	1	1	1	-1	1	0	1	1	-1	-1	-1	1	1	0	-1	-1	0	0	0	1	0	-1	1	1	0	1	0	1	0	0	CNA-high
417	1	1	1	0	0	1	0	0	1	-1	1	1	1	1	-1	1	-1	0	-1	0	-1	0	1	0	-1	0	0	0	1	0	0	0	0	0	-1	0	1	-1	0	-1	1	CNA-high
418	0	1	0	0	-1	1	-1	-1	1	-1	0	1	0	0	-1	0	-1	0	-1	-1	0	0	1	0	-1	-1	0	-1	-1	-1	0	1	0	-1	0	1	0	0	-1	0	-1	CNA-high
419	0	1	0	0	-1	1	-1	-1	1	-1	1	1	1	1	-1	1	-1	-1	0	0	1	1	0	0	-1	0	1	-1	-1	-1	0	1	-1	0	0	0	0	-1	1	0	0	CNA-high
420	-1	1	1	0	-1	0	-1	-1	1	-1	0	0	0	1	-1	0	-1	0	-1	-1	0	0	1	0	1	0	-1	1	1	0	0	0	-1	0	0	1	1	0	1	1	0	CNA-high
421	0	1	0	0	-1	1	-1	-1	1	-1	0	0	1	1	1	1	-1	-1	0	1	-1	1	0	0	0	0	-1	0	0	-1	1	0	0	0	0	1	1	-1	1	0	0	CNA-high
422	0	0	0	0	0	1	0	0	1	0	0	0	1	0	-1	1	1	1	0	0	0	0	1	0	0	1	0	0	0	0	0	0	-1	0	0	0	1	0	0	0	0	CNA-low
423	0	0	0	0	0	1	-1	-1	1	-1	0	0	0	0	-1	1	-1	-1	-1	-1	0	0	0	0	-1	-1	0	0	-1	-1	0	-1	-1	-1	-1	0	1	-1	-1	-1	1	CNA-high
424	0	0	0	0	-1	1	-1	-1	1	0	1	0	0	-1	-1	0	0	0	-1	-1	0	0	0	1	-1	-1	0	0	0	-1	0	0	0	-1	0	0	0	-1	-1	0	0	CNA-low
425	1	0	1	0	-1	1	-1	-1	1	-1	-1	0	0	1	0	0	-1	-1	0	0	-1	0	0	0	-1	1	1	0	0	-1	0	0	-1	0	0	0	0	-1	-1	0	0	CNA-low
426	-1	1	1	0	-1	1	-1	-1	1	-1	0	0	-1	1	-1	0	0	0	-1	-1	0	0	1	0	-1	-1	0	-1	-1	1	1	1	1	1	1	1	1	-1	0	0	0	CNA-high
427	0	0	0	0	-1	1	0	0	1	-1	0	0	-1	-1	1	1	-1	-1	-1	0	0	0	0	0	-1	1	0	0	0	-1	0	0	0	-1	1	1	1	-1	1	0	0	CNA-low
428	1	0	1	0	0	1	0	0	1	-1	-1	1	0	0	-1	1	-1	-1	-1	-1	-1	0	0	0	0	-1	0	0	-1	-1	0	0	0	-1	0	0	0	0	-1	0	0	CNA-low
429	-1	0	0	0	-1	1	0	0	0	0	0	0	0	0	-1	1	-1	-1	0	1	0	0	0	0	-1	0	-1	0	-1	0	0	0	-1	-1	0	-1	0	0	-1	-1	-1	CNA-low
430	-1	1	0	0	-1	1	-1	-1	0	-1	1	0	1	1	-1	0	0	1	-1	-1	0	1	0	0	0	0	0	1	1	-1	1	-1	-1	0	1	1	-1	-1	0	0	0	CNA-high
431	-1	0	1	0	-1	0	-1	-1	1	-1	-1	-1	-1	1	-1	1	0	1	1	-1	-1	0	0	0	-1	1	0	-1	-1	1	1	-1	-1	0	0	1	0	-1	1	-1	1	CNA-high
432	-1	1	1	0	-1	1	-1	-1	1	0	0	-1	1	0	-1	0	0	0	0	0	-1	0	0	0	0	0	0	0	0	-1	1	-1	-1	-1	0	0	0	-1	0	0	0	CNA-low
433	-1	0	0	0	-1	0	-1	-1	1	-1	0	1	0	0	-1	0	0	0	-1	-1	0	0	0	0	-1	-1	-1	0	0	-1	0	0	0	0	0	0	0	-1	-1	-1	-1	CNA-low
434	-1	1	0	0	-1	1	0	0	1	-1	0	-1	0	0	1	1	0	0	1	-1	-1	0	-1	0	0	0	0	-1	0	-1	0	0	0	-1	-1	-1	-1	0	0	0	0	CNA-low
435	0	1	1	1	-1	1	1	1	0	-1	-1	0	0	1	-1	1	-1	0	-1	-1	0	1	1	0	0	1	-1	0	1	1	0	1	-1	-1	1	1	1	0	1	0	-1	CNA-high
436	-1	1	0	0	-1	1	1	1	1	-1	1	-1	1	1	-1	1	-1	-1	1	1	-1	0	1	0	-1	1	-1	-1	-1	0	1	1	-1	-1	1	1	1	-1	0	1	-1	CNA-high
437	0	0	1	0	-1	0	-1	-1	1	-1	0	0	-1	0	0	0	-1	1	0	-1	0	0	1	0	0	0	-1	-1	0	-1	1	0	0	1	0	1	-1	0	1	-1	1	CNA-low
438	-1	0	0	0	-1	1	-1	-1	0	0	0	-1	-1	1	-1	0	-1	0	0	-1	0	0	0	0	0	0	0	-1	-1	-1	0	0	-1	0	0	0	0	-1	0	-1	-1	CNA-low
439	-1	-1	0	-1	-1	1	0	0	0	0	0	0	1	1	1	1	0	0	-1	-1	-1	0	0	0	-1	1	-1	0	0	-1	0	-1	-1	-1	-1	1	1	-1	-1	0	0	CNA-high
440	0	0	0	0	-1	0	-1	-1	1	-1	1	-1	1	1	-1	1	-1	0	-1	0	0	0	1	0	-1	0	0	-1	-1	0	0	1	-1	0	0	1	1	0	0	0	0	CNA-low

(Continued)

441	0	1	0	0	0	0	0	0	1	-1	1	1	1	0	0	1	0	-1	-1	-1	-1	1	1	0	-1	1	0	-1	1	0	0	0	0	-1	1	1	1	1	0	0	0	CNA-low
442	0	1	1	0	0	1	0	0	1	0	0	0	1	0	0	1	0	0	1	0	0	0	1	0	0	0	1	0	0	0	1	0	0	0	1	0	1	0	0	1	1	CNA-low
443	0	0	1	0	-1	1	-1	-1	1	-1	-1	0	0	0	0	1	-1	0	0	-1	-1	0	1	0	-1	-1	0	0	-1	-1	0	0	0	-1	-1	0	0	0	1	0	0	CNA-low
444	0	0	0	0	-1	0	-1	-1	1	-1	-1	-1	1	1	-1	1	0	-1	-1	1	0	0	0	1	-1	0	1	0	-1	0	0	1	1	0	0	0	0	-1	-1	0	0	CNA-low
445	0	0	0	0	-1	1	-1	-1	0	-1	0	0	0	0	0	0	-1	0	0	0	0	0	1	0	0	-1	0	0	0	0	0	0	0	0	0	0	0	-1	0	-1	0	CNA-low
446	-1	-1	0	0	-1	-1	0	0	0	0	0	0	0	0	0	0	0	0	0	0	0	0	-1	-1	-1	0	0	0	0	0	0	0	0	-1	-1	0	0	0	-1	0	0	CNA-low
447	0	0	1	1	-1	1	0	0	1	-1	1	0	1	0	0	0	-1	-1	0	1	-1	-1	-1	1	1	-1	0	0	0	0	0	1	0	-1	0	1	1	-1	1	0	0	CNA-high
448	1	1	0	0	-1	1	0	0	0	-1	1	0	0	0	-1	1	0	0	0	0	-1	1	0	0	0	0	0	0	0	-1	0	-1	0	-1	0	0	0	0	-1	0	0	CNA-low
449	-1	1	1	0	-1	1	-1	-1	1	-1	1	-1	-1	1	0	1	1	1	1	0	0	0	0	1	0	-1	-1	0	1	-1	1	-1	-1	-1	-1	0	-1	-1	1	0	1	CNA-high
450	0	1	0	0	-1	1	-1	-1	1	-1	0	1	0	1	-1	-1	-1	-1	-1	-1	-1	-1	0	0	-1	-1	0	1	1	-1	1	0	0	-1	-1	-1	1	-1	0	-1	-1	CNA-high
451	1	1	1	-1	-1	1	-1	-1	1	0	1	1	1	1	-1	-1	-1	-1	1	1	-1	1	-1	-1	0	0	-1	0	1	-1	0	1	1	-1	-1	1	1	0	0	-1	-1	CNA-high
452	0	0	0	0	-1	0	-1	-1	0	-1	0	0	1	1	0	0	0	1	0	-1	0	-1	1	1	-1	-1	0	0	0	0	0	-1	-1	0	0	1	0	0	0	-1	1	CNA-low
453	-1	1	1	0	-1	1	0	0	0	0	0	0	0	0	-1	0	0	0	-1	-1	0	0	0	0	0	0	0	0	0	-1	1	0	0	0	0	0	0	-1	0	-1	-1	CNA-low
454	-1	0	1	-1	-1	1	-1	-1	1	-1	-1	-1	1	1	-1	0	-1	0	-1	-1	0	0	1	0	1	1	0	0	-1	0	0	1	1	-1	-1	1	0	-1	1	0	0	CNA-high
455	0	1	1	0	-1	1	0	0	1	-1	1	0	0	1	-1	0	0	-1	-1	0	1	1	1	0	-1	1	-1	0	0	-1	0	1	0	-1	0	0	1	-1	1	-1	1	CNA-high
456	0	1	0	1	-1	0	-1	-1	1	0	0	0	0	1	-1	1	-1	-1	0	-1	1	1	0	1	-1	0	-1	-1	-1	-1	0	-1	0	-1	-1	1	1	-1	-1	-1	0	CNA-high
457	1	0	1	0	-1	1	-1	-1	1	0	1	-1	1	-1	0	0	0	0	-1	-1	-1	1	1	-1	0	1	-1	-1	-1	-1	0	1	0	1	0	1	1	1	1	1	1	CNA-high
458	-1	1	0	0	0	0	0	0	1	1	-1	-1	1	1	-1	1	0	0	0	0	-1	0	0	0	-1	-1	-1	0	0	-1	-1	0	0	0	0	0	0	0	0	0	0	CNA-low
459	-1	0	1	-1	-1	0	-1	-1	0	-1	1	0	1	1	-1	1	1	0	0	0	0	0	1	1	-1	0	1	-1	-1	-1	1	1	1	1	0	1	0	-1	1	0	0	CNA-high
460	0	1	0	0	-1	1	-1	-1	0	-1	1	-1	0	0	0	0	-1	-1	0	0	-1	0	0	0	-1	0	0	0	0	-1	0	0	0	0	-1	0	0	0	0	-1	-1	CNA-low
461	0	0	1	1	-1	1	-1	-1	1	-1	0	0	0	0	-1	1	1	-1	0	0	-1	0	1	1	0	1	0	0	1	1	1	1	1	-1	-1	1	1	-1	1	-1	1	CNA-high
462	-1	1	0	0	0	0	-1	-1	1	-1	1	1	1	0	0	0	1	0	-1	-1	0	-1	0	-1	-1	1	-1	-1	-1	0	0	-1	-1	1	1	0	0	0	0	0	-1	CNA-high
463	-1	1	0	0	-1	1	-1	-1	1	-1	0	0	1	1	0	0	-1	0	0	-1	-1	0	1	1	-1	0	1	0	0	1	0	-1	-1	1	-1	1	1	-1	0	0	0	CNA-high
464	0	0	0	0	-1	1	0	0	1	-1	-1	1	0	0	-1	0	-1	-1	0	0	-1	0	0	0	0	0	0	0	0	-1	0	0	-1	0	0	0	0	-1	0	0	0	CNA-low
465	0	1	1	0	-1	1	0	0	1	0	0	0	1	1	-1	0	0	0	0	0	-1	0	0	0	0	0	-1	0	0	-1	0	0	0	0	0	0	1	0	0	0	0	CNA-low
466	0	1	0	0	-1	1	0	0	1	-1	-1	1	0	0	-1	1	0	-1	0	-1	0	0	1	1	-1	-1	1	-1	-1	-1	0	1	1	0	0	0	0	-1	1	0	0	CNA-high
467	-1	1	1	1	-1	1	-1	-1	1	-1	0	-1	0	-1	1	1	-1	-1	0	-1	-1	0	1	1	0	-1	1	-1	0	-1	1	-1	-1	0	0	1	0	0	1	-1	1	CNA-high
468	-1	1	1	0	-1	1	-1	-1	1	-1	0	0	1	0	0	0	0	0	-1	0	1	1	1	0	-1	0	0	0	0	-1	1	1	1	-1	0	1	1	-1	1	0	0	CNA-high
469	0	1	0	0	-1	1	0	0	0	0	0	-1	-1	0	-1	0	0	0	0	-1	0	0	0	0	-1	0	0	0	0	-1	0	1	0	-1	-1	1	0	1	1	0	0	CNA-low
470	0	1	1	-1	-1	1	-1	-1	-1	-1	1	1	-1	1	0	1	-1	-1	-1	-1	-1	0	1	1	0	1	-1	1	-1	-1	0	-1	0	-1	1	0	1	-1	-1	1	1	CNA-high
471	-1	0	0	0	0	0	0	0	1	-1	-1	0	0	1	0	1	1	0	0	0	0	0	1	1	0	0	0	0	0	0	0	1	1	1	-1	0	0	0	1	0	0	CNA-low
472	-1	0	1	-1	-1	1	-1	-1	1	-1	0	0	1	1	-1	1	0	0	-1	-1	-1	0	1	0	-1	1	-1	-1	-1	0	0	1	1	1	0	1	1	-1	1	-1	0	CNA-high
473	0	1	1	0	-1	1	0	0	1	-1	0	0	1	1	0	1	0	-1	1	0	-1	-1	1	1	0	0	1	1	0	1	1	0	-1	-1	1	1	1	-1	-1	-1	1	CNA-high
474	-1	0	0	0	-1	0	-1	-1	0	-1	-1	0	1	0	-1	0	0	0	0	-1	0	0	1	1	-1	0	0	0	0	-1	0	0	0	0	0	-1	0	-1	0	0	0	CNA-low
475	-1	1	1	0	-1	1	-1	-1	1	-1	-1	-1	1	1	-1	0	-1	1	-1	-1	0	0	1	0	1	0	-1	-1	-1	-1	1	0	-1	0	1	1	0	-1	0	0	0	CNA-high
476	0	0	1	0	-1	1	1	1	1	-1	1	-1	-1	0	-1	1	-1	0	0	-1	-1	-1	1	1	-1	0	0	0	0	1	1	0	-1	-1	1	0	0	0	1	0	0	CNA-high
477	0	0	1	0	-1	1	-1	-1	1	-1	0	0	0	0	-1	1	0	1	-1	-1	0	0	0	0	-1	-1	0	-1	0	-1	1	-1	0	0	-1	0	0	-1	0	0	-1	CNA-low
478	0	1	1	1	0	1	-1	-1	1	0	1	0	1	0	-1	1	1	1	1	1	0	0	1	0	0	1	1	0	0	0	0	1	-1	0	1	0	0	1	0	-1	1	CNA-high
479	0	1	1	0	-1	1	-1	-1	1	-1	0	-1	1	0	-1	0	0	-1	-1	-1	-1	1	1	1	-1	1	-1	0	0	-1	1	-1	1	-1	1	1	1	-1	1	1	-1	CNA-high
480	-1	1	0	1	-1	0	-1	-1	0	0	-1	-1	0	0	1	1	1	1	-1	-1	1	1	-1	1	-1	-1	1	1	-1	-1	-1	0	-1	0	0	-1	-1	1	-1	0	0	CNA-high

(Continued)

481	-1	1	0	0	-1	1	-1	-1	1	-1	0	0	1	1	-1	1	1	1	-1	-1	0	0	1	1	-1	-1	0	1	-1	-1	1	1	1	1	0	-1	-1	-1	1	0	0	CNA-high
482	0	-1	0	0	-1	1	-1	-1	0	0	0	1	1	0	-1	1	1	0	0	0	0	-1	0	0	-1	1	-1	0	0	0	0	-1	-1	0	0	1	1	-1	1	0	0	CNA-low
483	1	1	1	1	0	1	0	0	1	0	0	0	1	1	0	0	1	0	0	0	0	0	1	0	0	0	1	1	0	-1	0	1	0	1	1	1	1	1	1	1	1	CNA-low
484	0	0	1	-1	-1	1	-1	-1	1	-1	0	0	-1	1	-1	1	-1	-1	0	1	0	0	1	0	0	0	0	0	1	1	-1	0	-1	-1	0	0	0	-1	1	0	0	CNA-high
485	0	0	1	1	-1	0	0	0	1	-1	0	0	-1	1	-1	0	-1	1	0	-1	0	1	-1	1	-1	1	-1	-1	-1	1	0	-1	-1	-1	0	0	1	0	-1	0	0	CNA-high
486	-1	1	1	1	-1	1	0	0	1	-1	-1	-1	1	0	-1	1	-1	0	0	0	0	-1	0	0	0	-1	-1	-1	-1	-1	1	1	-1	1	0	1	1	-1	0	0	0	CNA-high
487	0	1	0	0	-1	1	-1	-1	1	-1	1	-1	1	0	-1	0	0	0	-1	-1	0	-1	1	0	1	-1	-1	-1	0	0	0	1	1	0	0	1	0	0	1	0	1	CNA-high
488	1	0	0	0	-1	1	0	0	0	0	0	0	0	0	-1	1	-1	-1	0	0	0	0	0	0	-1	0	-1	-1	-1	0	0	-1	-1	0	0	1	1	1	0	-1	1	CNA-low
489	0	-1	-1	0	-1	1	0	0	1	-1	0	0	-1	1	-1	1	0	0	-1	-1	1	1	0	0	-1	1	-1	0	0	-1	-1	1	0	-1	1	1	1	-1	1	0	0	CNA-high
490	0	0	0	0	-1	1	0	0	1	-1	0	0	0	0	0	0	-1	1	-1	-1	0	0	0	0	-1	-1	-1	0	-1	-1	0	0	0	0	0	0	0	0	0	0	0	CNA-low
491	0	0	0	0	-1	1	0	0	1	-1	0	0	1	1	-1	1	0	1	-1	0	-1	0	0	0	-1	1	-1	0	0	0	0	1	1	1	1	1	1	-1	0	0	0	CNA-low
492	0	0	0	0	-1	0	-1	-1	0	0	0	-1	0	-1	0	1	0	-1	-1	0	-1	1	1	0	1	0	-1	1	0	-1	0	0	0	-1	1	-1	-1	-1	1	0	0	CNA-low
493	0	1	1	1	-1	1	-1	-1	1	0	0	0	0	1	0	1	0	-1	0	0	-1	0	1	1	1	-1	0	0	0	-1	0	0	0	0	1	0	0	0	-1	0	0	CNA-low
494	-1	0	1	0	-1	1	-1	-1	1	-1	0	0	-1	0	0	0	-1	1	-1	-1	1	1	1	0	-1	0	0	1	1	0	0	-1	-1	-1	-1	1	1	-1	1	1	0	CNA-high
495	-1	1	1	0	-1	0	0	0	0	-1	-1	-1	1	0	-1	0	0	-1	-1	-1	-1	0	1	0	0	1	1	0	1	0	1	1	0	0	0	1	1	-1	1	0	1	CNA-high
496	0	0	0	0	0	0	0	0	1	-1	-1	-1	0	0	-1	0	0	0	-1	0	0	0	0	0	0	0	0	0	0	0	0	0	0	0	1	1	1	-1	1	0	0	CNA-low
497	-1	1	0	0	-1	1	-1	-1	1	-1	0	-1	1	1	0	1	-1	-1	-1	-1	0	0	1	0	-1	-1	1	-1	-1	0	0	1	0	1	1	1	-1	-1	1	0	0	CNA-high
498	0	1	0	0	-1	1	0	0	1	-1	0	0	0	1	-1	0	0	-1	0	0	0	0	1	0	0	-1	-1	-1	1	-1	1	1	0	0	-1	0	0	-1	1	0	0	CNA-low

Chromosomal arm-level copy number status is shown as follows: 0, diploid; 1, chromosomal gain; -1, chromosomal deletion.
